# Light-Powered Reversible Guest Release and Uptake from Zn_4_L_4_ Capsules

**DOI:** 10.1021/jacs.2c10084

**Published:** 2023-02-08

**Authors:** Amit Ghosh, Laura Slappendel, Bao-Nguyen T. Nguyen, Larissa K. S. von Krbek, Tanya K. Ronson, Ana M. Castilla, Jonathan R. Nitschke

**Affiliations:** Yusuf Hamied Department of Chemistry, University of Cambridge, Lensfield Road, Cambridge, CB2 1EW, United Kingdom

## Abstract

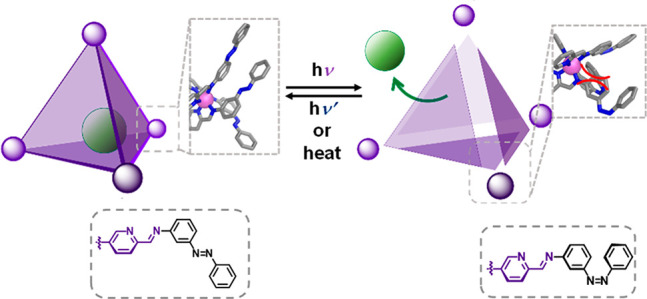

A strategy for light-powered guest release from a tetrahedral capsule has been developed by incorporating azobenzene units at its vertices. A new Zn_4_L_4_ tetrahedral capsule bearing 12 diazo moieties at its metal-ion vertices was prepared from a phenyldiazenyl-functionalized subcomponent and a central trialdehyde panel. Ultraviolet irradiation caused isomerization of the peripheral diazo groups from the thermodynamically preferred *trans* configuration to the *cis* form, thereby generating steric clash and resulting in cage disassembly and concomitant guest release. Visible-light irradiation drove cage re-assembly following re-isomerization of the diazo groups to the *trans* form, resulting in guest re-uptake. A detailed ^19^F NMR study elucidated how switching led to guest release: each metal vertex tolerated only one *cis*-azobenzene moiety, with further isomerization leading to cage disassembly.

Discrete supramolecular architectures^1^ and containers^2^ can undergo structural re-configuration upon receiving external stimuli, which can generate useful functions.^3^ Stimuli-responsive guest uptake and release by supramolecular capsules^4^ is one such function, potentially enabling new means of drug delivery,^5^ pesticide release,^6^ chemical separations,^7^ and purifications.^8^ Among stimuli,^9^ light is particularly useful, as it is easy to apply from cheap sources and does not result in the accumulation of waste products even after multiple cycles.^10^

Photoswitches have been used to control encapsulation and release processes.^11^ For example, Clever and co-workers recently prepared light-responsive coordination cages based upon dithienylethene^12^ and diazocine^13^ chromophores, which can undergo structure transformations that prompt guest uptake and release. The Fujita group reported photoisomerization of inward-facing azobenzene moieties in a spherical complex, where switching resulted in reversible guest uptake.^14^ In these systems, the photochromic moieties form integral parts of the ligand backbone, necessitating a re-design of the cage to target new guests. A more general and modular approach would decouple the photochrome from the cage framework, to render guest-binding orthogonal to the photoswitching process that governs guest uptake and release.

Subcomponent self-assembly provides a modular construction technique for metal–organic capsules that respond to different stimuli.^15^ Systems have thus been designed using acid and base as stimuli to release and exchange cargos between two capsules, through the selective disassembly of one.^16^ Systems containing two or three capsules have been reported, where selective disassembly of individual cages and the release of their guests were guided by the application of external signals.^17^ In each case, hosts disassemble following the addition of chemical signals, resulting in the accumulation of chemical waste products after each addition. The use of light or heat^18^ as a stimulus would avoid the formation of such byproducts, sidestepping problems associated with waste buildup.

Azobenzenes serve as key photochromic moieties within supramolecular assemblies, as they not only alter dipole moment^14^ but also change shape to alter properties in useful ways.^19^ Building upon reports of photoinduced guest uptake and release from multilayer films^20^ and nanotubes,^21^ herein we report a discrete and solution-based^22^ self-assembled Zn_4_L_4_ tetrahedral cage, *trans*-**1** (Figure 1), functionalized with 12 azobenzene units at its vertices. The photoswitching of these azobenzenes toward the *cis* configuration results in progressive buildup of steric strain, culminating in the release of an anionic guest bound within the cage cavity.

**Figure 1:**
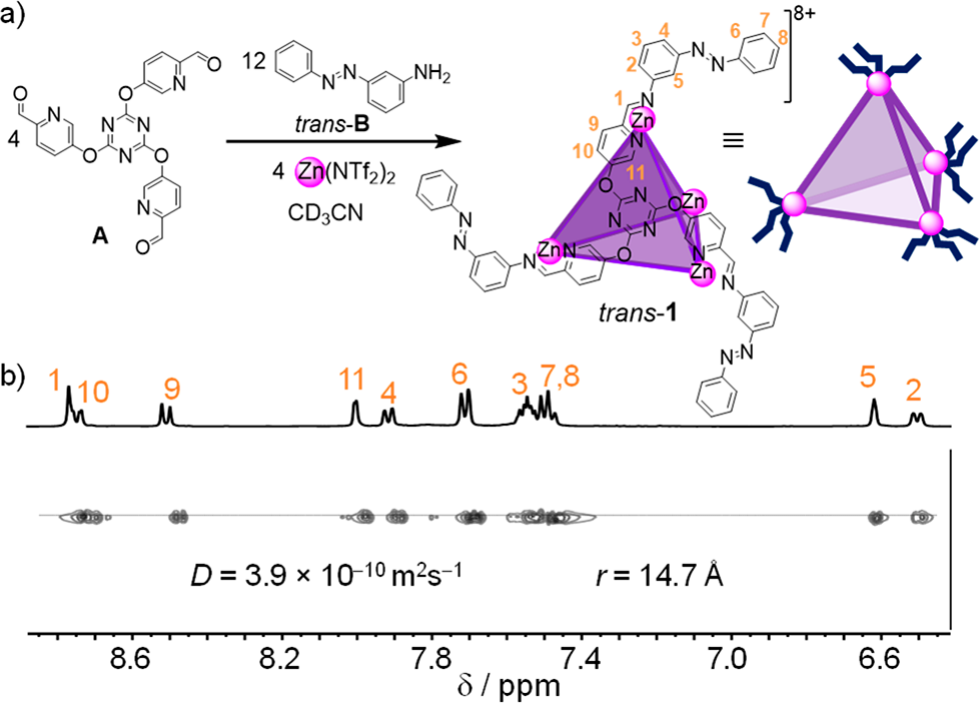
a) Assembly of subcomponents **A** and *trans*-**B** with Zn^II^ produced cage *trans*-**1**. b) ^1^H and DOSY NMR (400 MHz, CD_3_CN, 298 K) spectra of *trans*-**1**.

Photoswitchable azobenzene-functionalized subcomponent 3-(phenyldiazenyl)aniline **B** was synthesized following a literature procedure.^23^**B** forms as the *trans* isomer (*trans*-**B**), which is stable in the absence of light. Prior to investigating cage photoisomerization, we first tested the photoswitching ability of subcomponent *trans*-**B**. Irradiation at 350 nm generates the *cis* isomer (*cis*-**B**), while irradiation at 500 nm or heating reverses the process (Figure S20). Upon exposure to UV light, a new set of signals appeared in the ^1^H NMR spectrum, assigned to *cis*-**B**. The isomerization did not occur quantitatively. A photostationary state (PSS) was reached after 10 min and determined to consist of 71% *cis*-**B** by NMR integration (Figure S21). Upon irradiation at 500 nm, *trans*-**B** was fully regenerated within 30 min; complete conversion to *trans*-**B** also occurred after heating the PSS to 75 °C for 600 min, revealing a half-life of 46 min at that temperature (Figures S22 and S23).

Tritopic formylpyridine subcomponent **A** was prepared from 2,4,6-trichloro-1,3,5-triazine and 5-hydroxypicolinaldehyde (see Supporting Information Section S2). Subcomponents *trans*-**B** (12 equiv) and **A** (4 equiv) reacted with zinc(II) bis(trifluoromethanesulfonyl)imide (triflimide, Tf_2_N^–^, 4 equiv) in acetonitrile to give tetrahedral capsule *trans*-**1** (Figure 1a) as the uniquely observed product. *Trans*-**1** was characterized by ^1^H NMR, ^1^H–^1^H COSY, DOSY, and electrospray ionization mass spectroscopy (ESI-MS) (Figures S3–S10). The ^1^H NMR spectra of *trans*-**1** exhibited only one set of ligand signals, consistent with a *T*-symmetric tetrahedral product (Figure 1b).^24^ A ^1^H DOSY spectrum of the cage corroborates the presence of a single component with a diffusion coefficient (*D*) of 3.9 × 10^–10^ m^2^·s^–1^ (Figure 1b). We infer that the three *trans*-diazo moieties surrounding each Zn^II^ center of *trans*-**1** extend from each other without steric collisions (Figure 2b).

**Figure 2:**
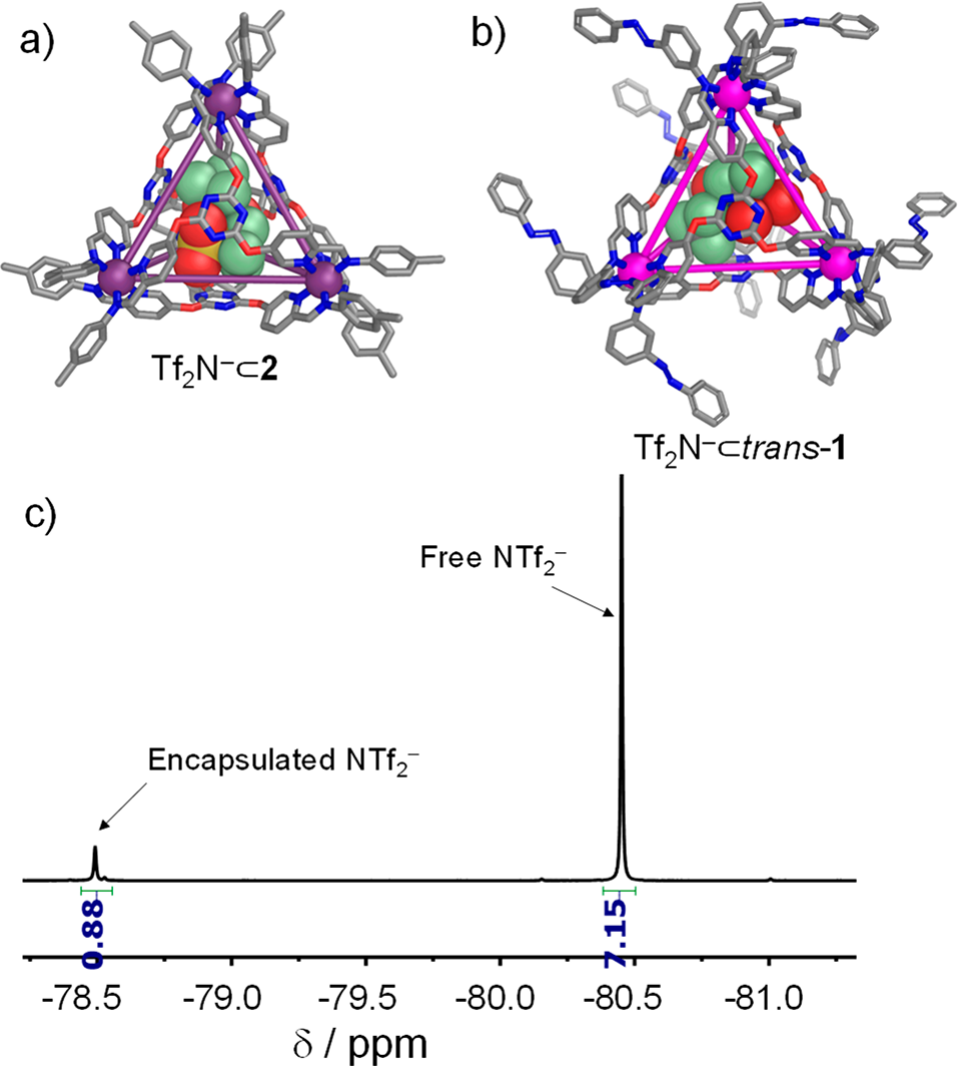
(a) Crystal structure of Tf_2_N^–^⊂**2**. Tf_2_N^–^ shown in space-filling mode: N, blue; S, yellow; O, red; C, gray; F, pale green. Disorder, unbound counterions, and solvent of crystallization are omitted for clarity. Attempts to obtain single crystals suitable for X-ray diffraction of an analog of cage **2** with Zn^II^ instead of Fe^II^ were unsuccessful. (b) MM2-optimized molecular model of *trans*-**1**, based on the structure of **2**. (c) ^19^F NMR (376 MHz, CD_3_CN, 298 K) spectrum of Tf_2_N^–^⊂*trans*-**1**.

Although multiple attempts to crystallize cage *trans*-**1** were unsuccessful, single crystals suitable for X-ray diffraction of analogous cage **2**, composed of subcomponent **A**, iron(II) triflimide, and *p*-toluidine in place of **B**, were obtained following slow diffusion of diethyl ether into a solution of **2** in acetonitrile. As shown in Figure 2a, Tf_2_N^–^ was found inside the 205 Å^3^ cavity of cage **2**. Furthermore, the ^1^H NMR and ^19^F NMR spectra of cage **2** show two sets of signals for the empty and triflimide-binding cage in slow exchange on both NMR time scales (Figures S15 and S16).

The ^19^F NMR spectrum (Figure 2c) of cage *trans*-**1** displayed two sets of signals for the Tf_2_N^–^ counter anion, in a 7:1 integral ratio. This observation is consistent with one anion being bound within the cavity of **1** (Figure 2b), as observed in the crystal structure of **2** (Figure 2a), with the other seven free in solution.

Since attempts to encapsulate neutral guests by *trans*-**1** provided no ^1^H NMR evidence of binding (Figure S57), subsequent host–guest studies were conducted with anions only. PF_6_^–^, TfO^–^, and BF_4_^–^ were observed to bind within *trans*-**1** (Figure S47). Progressive addition of the tetrabutyl ammonium salt of each anion to a solution of Tf_2_N^–^⊂*trans*-**1** showed evidence of triflimide displacement by ^1^H NMR (Figures S37, S39, and S43) and ^19^F NMR (Figures S38, S40, and S44). In each case, the ^19^F NMR resonances for the guest molecules were broadened and shifted, suggesting fast-exchange binding of anions within *trans*-**1** on the NMR time scale even at 233 K (Figures S51–S55). We attribute the observed differences in exchange properties to the smaller volumes of PF_6_^–^, TfO^–^, and BF_4_^–^, as compared to Tf_2_N^–^ (Table S1). Moreover, attempts to prepare the cage with Zn(OTf)_2_ and Zn(BF_4_)_2_ instead of Zn(NTf_2_)_2_ appeared successful by ^1^H NMR (Figures S11 and S13). Studies involving these anions were not pursued further because slow-exchanging triflimide greatly facilitated analysis by NMR (see below).

We then investigated light-driven guest release for Tf_2_N^–^⊂*trans*-**1** (Figure 3). Upon irradiation with 350 nm light, the *trans*-azobenzene subcomponents at the periphery of *trans*-**1** underwent photoisomerization to the *cis* isomer. This photochemical transformation was followed by ^1^H NMR and ^19^F NMR. The ^1^H NMR spectra became complex, with multiple sets of ligand signals, suggesting the formation of a mixture of cages containing both *cis*- and *trans*-diazo moieties (Figure S26).

**Figure 3:**
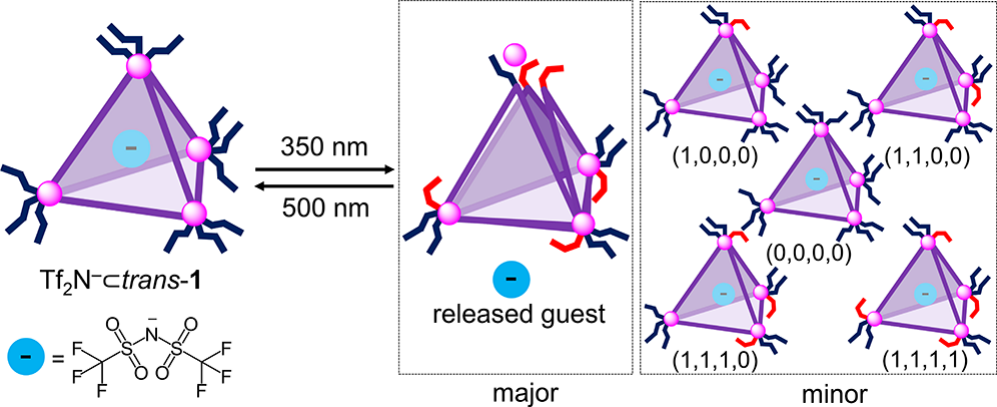
Cartoon of the photoswitching of cage Tf_2_N^–^⊂*trans*-**1**, illustrating the opening of the cage after a fifth azobenzene switches, along with the five observed guest-binding states of the cage, in which 0–4 azobenzene residues have switched to *cis*. **B** residues with the *trans* and *cis* configurations are colored blue and red, respectively.

Encouragingly, photoisomerization led to the partial disassembly of the cage, with appearance of ^1^H NMR signals for subcomponents **A** and *cis*-**B** (Figure S27). We infer this disassembly to have occurred as a result of *cis*-azobenzenes occupying more of the volume near the metal center than in the case of the *trans* isomer, leading to steric collisions between subcomponents at the metal-ion vertices of the cage. When the disassembled system was irradiated at 500 nm, the azo moieties converted back to the *trans* configuration and re-assembled to form Tf_2_N^–^⊂*trans*-**1**, as confirmed by ^1^H NMR (Figure S28).

The photoisomerization abilities of **B** were thus maintained following integration into the metallocage framework of **1**. Upon removal of the light source and subsequent heating to 75 °C, the disassembled system started transforming into Tf_2_N^–^⊂*trans*-**1** with time (Figure S29). The process was monitored by ^19^F NMR, revealing a half-life of 38 min (Figure S32), similar to that of free subcomponent photoisomerization. After 600 min at 75 °C, the spectrum was identical to that of freshly prepared Tf_2_N^–^⊂*trans*-**1**.

The ^19^F NMR spectra of Tf_2_N^–^⊂*trans*-**1** during irradiation were more straightforward to interpret than the corresponding ^1^H NMR spectra, allowing the course of cage isomerization to be followed. As noted above, prior to UV irradiation, only one ^19^F NMR signal was observed for the encapsulated Tf_2_N^–^ guest. After UV irradiation, however, five distinct bound Tf_2_N^–^ signals were observed (Figure 4). We infer that these species incorporated from 0 to 4 *cis*-diazo moieties per cage (Figure 3), with each cage tolerating a maximum of one *cis*-diazo per vertex before opening to release the guest (Figure S71). Evolution of the ^19^F NMR signals (Figure S30) is consistent with the most upfield peak corresponding to cages with 4 *cis*-diazo moieties per cage, whereas the most downfield peak corresponds to *trans*-**1**, i.e., a cage without *cis*-diazo moieties (Figure 4). Analysis of the extent of progressive photoisomerization in cage **1** is difficult to quantify because the triflimide ^19^F NMR signal reports only on intact cages, with no straightforward means available to quantify the *cis* vs *trans* population of the ligand that is no longer incorporated into a cage (Figure S34). Figure 5 shows the time course of the reverse of this process, as free Tf_2_N^–^ is encapsulated by cages that incorporate progressively more *trans* residues as the system thermally relaxes from a state in which most **B** residues are *cis*.

**Figure 4:**
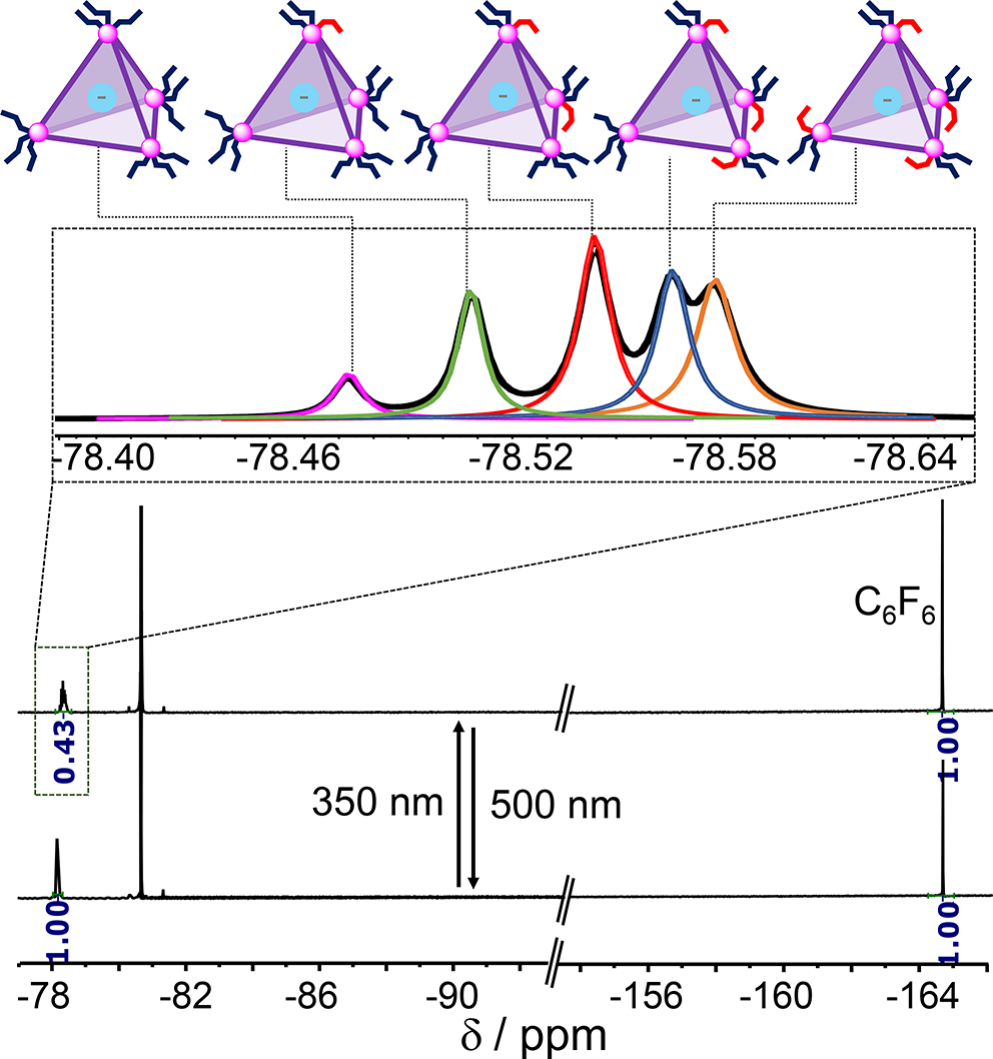
^19^F NMR spectra (CD_3_CN, 376 MHz, 298 K) show 43% encapsulated/57% released Tf_2_N^–^ guest after UV irradiation for 10 min; the encapsulated Tf_2_N^–^ is quantified by NMR integration with respect to hexafluorobenzene as an internal standard. The five distinct guest-encapsulating cages, incorporating 0–4 *cis*-azobenzene residues, gave rise to five ^19^F NMR signals, which were deconvoluted as shown.

**Figure 5:**
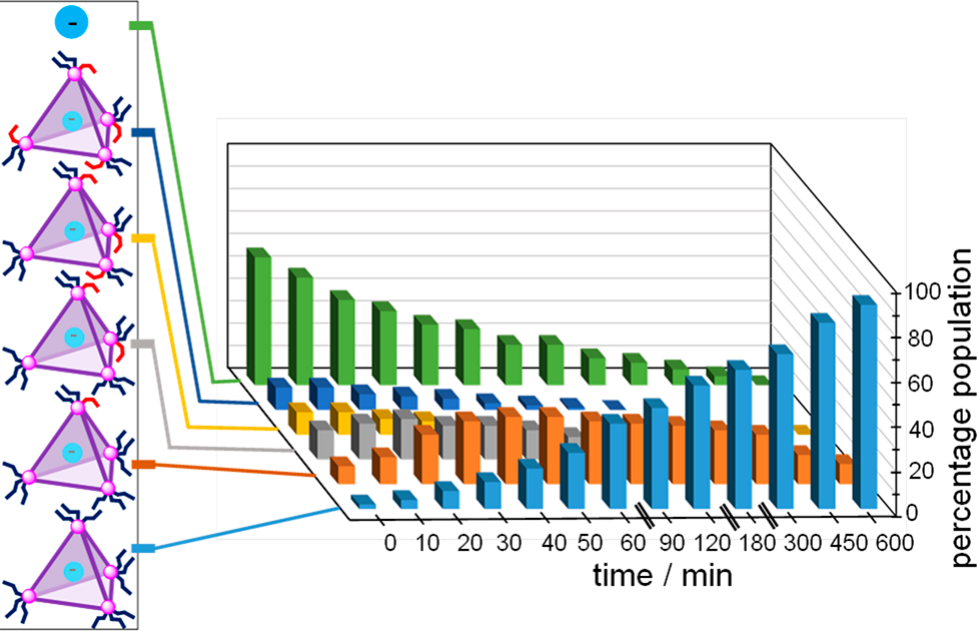
Re-formation of cages incorporating progressively more residues of *trans*-**B** during thermal relaxation while heating to 75 °C, derived from integration of ^19^F NMR spectra of the encapsulated triflimide guest (Supporting Information, Figure S30). The percentage population value shown is normalized to the maximum amount of anion that can be encapsulated.

We then quantified the release process by using hexafluorobenzene as an internal standard (Figure 4). The integration of ^19^F NMR signals revealed 57% release of the Tf_2_N^–^ guest 10 min after irradiation (Figure S33). The system showed no evidence of fatigue after 10 cycles of alternating irradiation with 350 and 500 nm light (Figure 6b). Thermal recovery at 75 °C (Figure 6c) also proved entirely reversible, although with a longer cycle time of 600 min (Figure S31) vs 30 min for the purely light-driven process of Figure 6b. The light-triggered release and uptake of the other anionic guests, PF_6_^–^, TfO^–^, and BF_4_^–^, was also explored and confirmed by ^1^H and ^19^F NMR analyses (Figures S60–S69).

**Figure 6:**
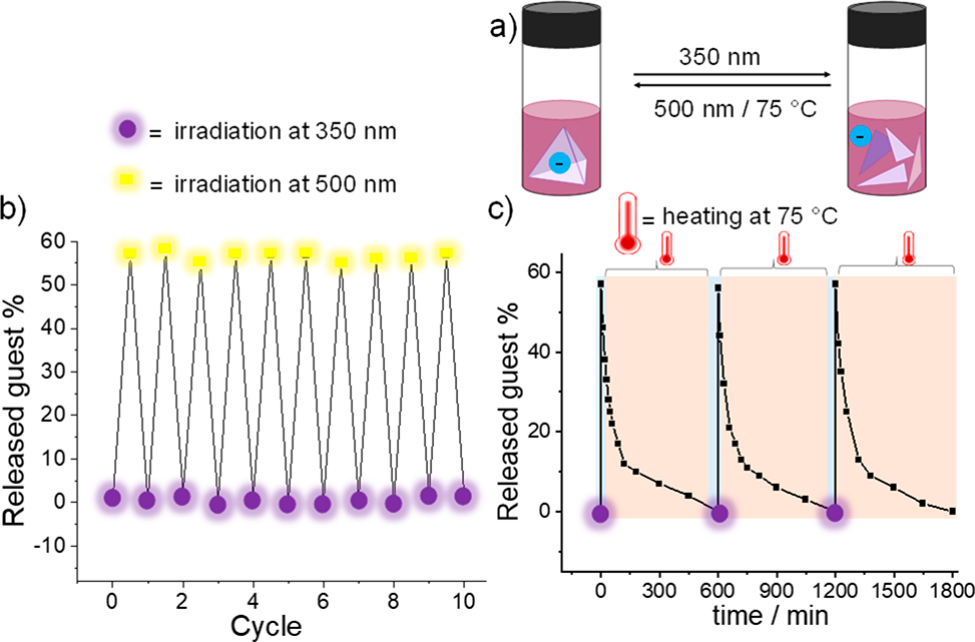
a) Cartoon representation of reversible release and uptake of Tf_2_N^–^ guest by UV light and visible light or heat. b) Ten cycles of guest release driven by irradiation at 350 and 500 nm in CD_3_CN in an alternating sequence. c) Three such cycles using light (350 nm) and temperature (75 °C) as stimuli; in both cases no evidence of fatigue was observed.

Although the cage **1** can only encapsulate non-biologically relevant anions, larger cages constructed from polytopic aldehyde subcomponents^25^ may enable this modular and straightforward means of light-powered guest release and uptake to find applications across different fields, potentially including switchable catalysis, drug delivery, and chemical purification.

In this latter application, a cargo molecule might be selectively bound and then released by light following flow to a different location. The ability to use light as a stimulus may also allow for stimulus transduction, where illumination releases guests as signals within more complex chemical systems.^26^

## Supporting Information Available

Complete experimental details and characterization data (PDF)
