# Supplementary material for: Light-Powered Reversible Guest Release and Uptake from Zn4L4 Capsules
Source: J Am Chem Soc. 2023 Feb 8;145(7):3828–32. doi: 10.1021/jacs.2c10084 (PMC9951218; doi:10.1021/jacs.2c10084)
Supplement: Supplementary file 1 — ja2c10084_si_001.pdf [file ja2c10084_si_001.pdf]

Light-Powered Reversible Guest Release and Uptake from Zn_4L_4 Capsules

Amit Ghosh, Laura Slappendel, Bao-Nguyen T. Nguyen, Larissa K. S. von Krbek, Tanya K. Ronson, Ana M. Castilla, Jonathan R. Nitschke*

Yusuf Hamied Department of Chemistry, University of Cambridge,

Cambridge CB2 1EW, UK.

jrn34@cam.ac.uk

Supporting Information

Table of Contents

S1 General Information........3

S2 Synthesis and Characterization of Subcomponent **A**........4

S3 Self-Assembly and Characterization of *trans*-**1**........6

S4 Self-Assembly and Characterization of $Tf_2N^{-}\subset$**2**........13

S5 Investigation of Photoswitching Properties of Subcomponent **B**........17

S6 Investigation of Photoswitching Properties of $Tf_2N^{-}\subset$*trans*-**1**........19

S6.1 Characterization of Disassembly and Assembly of $Tf_2N^{-}\subset$*trans*-**1** Using ^{1}H NMR........20

S6.2 Characterization of Guest (Tf_2N^{-}) Release and Uptake Using ^{19}F NMR........22

S6.3 Characterization of Photo-switching of *trans*-**1** Using UV/Vis Spectra........25

S7 Host-Guest Studies Using $Tf_2N^{-}\subset$*trans*-**1**........26

S7.1 Encapsulation of PF_6^-........27

S7.2 Encapsulation of Guest TfO^-........28

S7.3 Encapsulation of Guest BF_4^-........30

S7.4 Comparison of ^{1}H and ^{19}F NMR spectra of All of the Host-Guest complexes........32

S7.5 Competitive Binding Studies........32

S7.6 Variable Temperature Studies........35

S7.7 Investigation with Neutral Guests........37

S8 Investigation of Photoswitching Properties of *Trans*-**1** Encapsulating Different Guests 39

S8.1 Reversible photoswitching of cage $PF_6^{-}\subset$trans-**1**........40

S8.2 Reversible photoswitching of cage $TfO^{-}\subset$*trans*-**1** 41

S8.3 Reversible photoswitching of cage $BF_4^{-}\subset$*trans*-**1** 42

S8.4 Reversible Photoswitching of Cage *Trans*-**1**·$[OTf]_8$ 43

S8.5 Reversible Photoswitching of Cage *Trans*-**1**·$[BF_4]_8$ 44

S9 X-ray crystallography 45

S10 Volume Calculations 47

S11 MM3 Models and Calculated Energies 48

S12 Control Experiments 49

S13 References 52

S1 General Information

Unless otherwise specified, all reagents were purchased from commercial sources and used as received. Ligand **A** was prepared from commercially available cyanuric chloride and 5-Hydroxypicolinaldehyde. Compound **B** was synthesized according to reported procedures.[1] Self-assembly reactions were performed in CD_3CN.

NMR spectra were recorded using the following NMR spectrometers: Bruker 400 MHz Avance III HD smart probe (^1H, ^{13}C, ^{19}F, 2D NMR), Bruker Avance 500 MHz DCH cryoprobe (^1H, ^{19}F), Bruker Avance 500 MHz TCI cryoprobe (^1H, ^1H-DOSY). Chemical shifts of the NMR spectra are reported relative to $CDCl_3$ (^1H NMR: δ = 7.26 ppm, ^{13}C NMR: δ = 77.0 ppm), and CD_3CN (^1H NMR: δ = 1.94 ppm, ^{13}C NMR: δ = 118.3 and 1.8 ppm). Data for ^1H NMR spectra were reported as follows: chemical shift (ppm), peak shape (s = singlet, d = doublet, dd = doublet of doublets, ddd= doublet of doublets of doublets, t = triplet, m = multiplet), coupling constant (Hz), and integration. Data for ^{13}C NMR and ^{19}F NMR are reported in terms of chemical shift (ppm).

Deconvolution of peak clusters with calculated Lorentzian peaks gave rise to the speciation profiles. Baseline corrections were performed using the MestReNova Bernstein polynomial algorithm prior to this analysis. All processing operations were manually checked to ensure that the processed spectra accurately represented the raw data.

High resolution electrospray ionisation mass spectra (ESI–HRMS) were recorded on a Waters Synapt G2-Si instrument.

The irradiations at 350 nm and 500 nm were performed in-situ placing the NMR tubes or the cuvettes inside a Rayonet photochemical chamber reactor (40 cm deep, 25 cm diameter, 16 × 14 W light sources, operating temperature 32 °C).

S2 Synthesis and Characterization of Subcomponent A

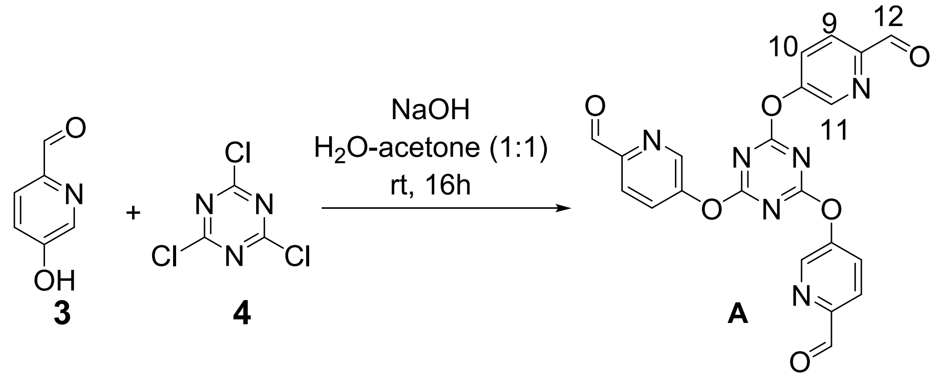

2,4,6-trichloro-1,3,5-triazine (316 mg, 1.71 mmol, 1.0 eq.) in acetone (100 mL) was added dropwise to 5-hydroxypicolinaldehyde (631 mg, 5.13 mmol, 3.0 eq.) and NaOH (205 mg, 5.13 mmol, 3.0 eq.) in water (100 mL). The solution was stirred at room temperature for 16 h. The acetone was removed *in vacuo* then the rest was extracted in $CHCl_3$-^{i}PrOH (3 : 1), and wash with water. The organic layer was dried over Na_2SO_4, concentrated *in vacuo*. The residue was purified by column chromatography (SiO_2; Hexane : EtOAc, 2 : 1, R_f = 0.35) yielding a white solid (450 mg, 1.01 mmol, 59%).

^{1}H NMR (400 MHz, $CDCl_3$): δ 10.05 (d, J = 0.8 Hz, 3H, 12-H), 8.72 (dd, J = 2.6, 0.6 Hz, 3H, 11-H), 8.06 (dd, J = 8.4, 0.6 Hz, 3H, 10-H), 7.86 (ddd, J = 8.4, 2.6, 0.8 Hz, 3H, 9-H) ppm. **^{13}C NMR** (100 MHz, CD_3CN): δ 191.7, 173.2, 150.9, 150.8, 143.9, 130.0, 122.8 ppm. HR-ESI-MS [POS] $[M+H]^+$: m/z = 445.0905 (calc. for $C_{21}H_{13}N_6O_6$: m/z 445.0897).

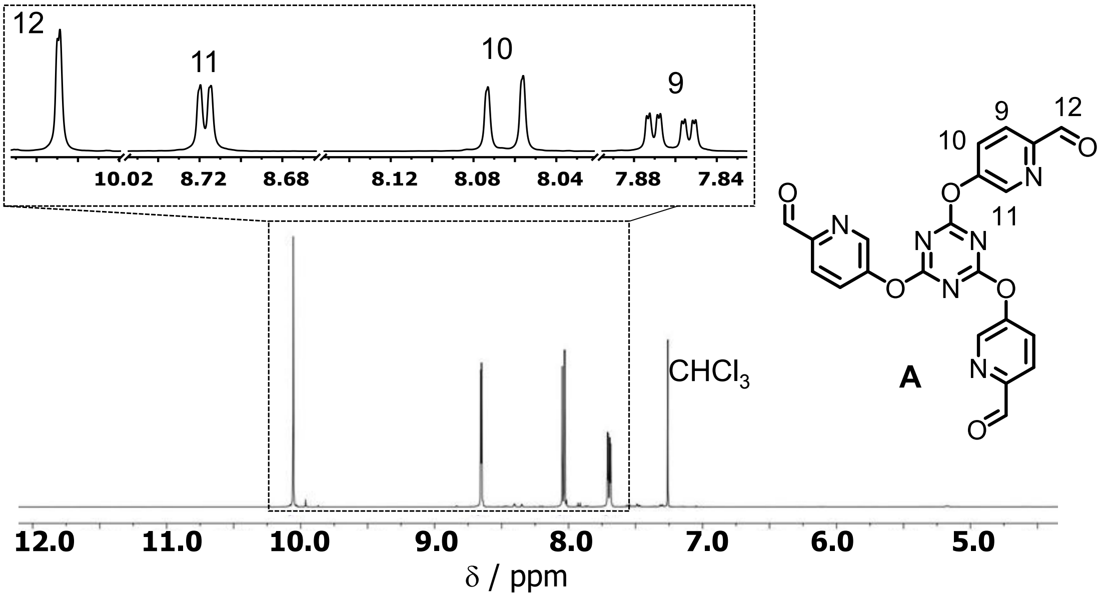

Figure S1. ^{1}H NMR spectrum of compound **A** (400 MHz, $CDCl_3$, 25 °C).

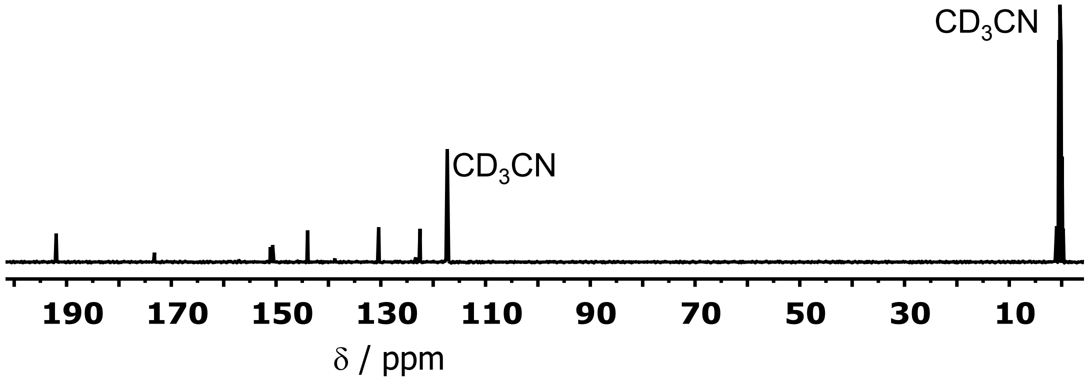

Figure S2. ^{13}C NMR spectrum of compound **A** (100 MHz, CD_3CN, 25 °C).

S3 Self-Assembly and Characterization of *trans*-1

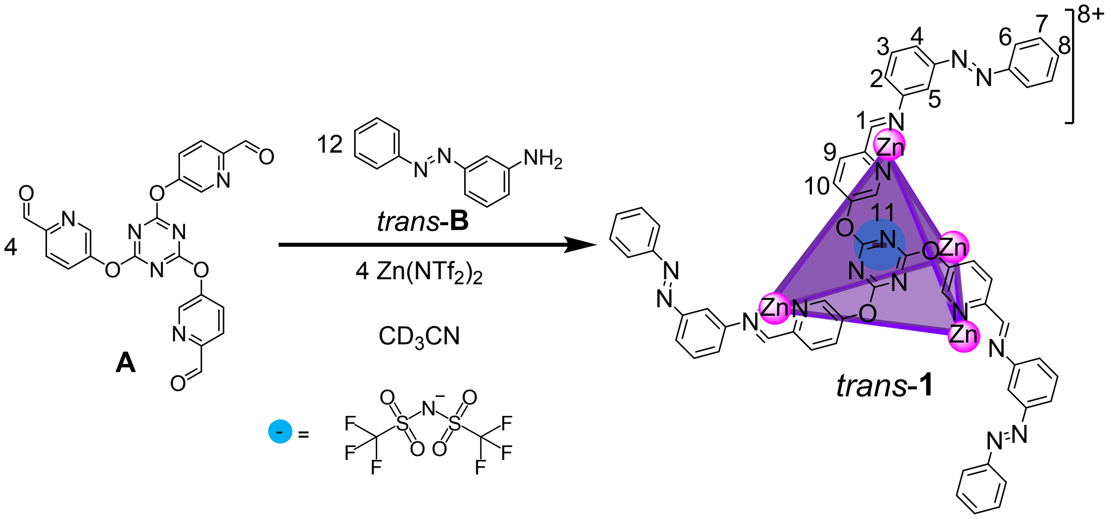

In a NMR tube, ligand **A** (8.80 mg, 19.8 µmol, 4.0 equiv) in 500 µL of CD_3CN was combined with ligand **B** (11.7 mg, 59.4 µmol, 12.0 equiv). To this mixture, $Zn(NTf_2)_2$ (12.4 mg, 19.8 µmol, 4.0 equiv) was added and subsequently the NMR was recorded. *Trans*-**1** was the uniquely-observed product.

^{1}H NMR (400 MHz, CD_3CN): δ 8.77 (s, 12H, 1-H), 8.75 (dd, J = 8.4, 0.6 Hz, 12H, 10-H), 8.51 (dd, J = 8.4, 2.6 Hz, 12H, 9-H), 8.00 (dd, J = 2.6, 0.6 Hz, 12H, 11-H), 7.91 (ddd, J = 8.0, 2.4, 1.2 Hz, 12H, 4-H), 7.71 (dd, J = 8.0, 1.2 Hz, 24H, 6-H) 7.58-7.45 (m, 48H, 3-,7-,8-H), 6.62 (t, J = 2.4 Hz, 12H, 5-H), 6.50 (ddd, J = 8.0, 2.4, 1.2 Hz, 12H, 2-H) ppm. **^{13}C NMR** (100 MHz, CD_3CN): δ 173.4, 166.2, 154.2, 153.4, 149.0, 145.8, 144.4, 138.2, 133.4, 133.0, 132.5, 130.8, 126.5, 125.0, 124.4, 123.0, 114.7 ppm. **^{19}F NMR** (CD_3CN, 376 MHz, referenced to C_6F_6): δ -78.5 (s, *endo* Tf_2N^-), -80.5 (s, *exo* Tf_2N^-) ppm. **ESI-MS**: m/z = 791.5 [**1**-2(NTf$_2$)]$^{6+}$, 1005.8 [**1**-3(NTf$_2$)]$^{5+}$, 1327.2 [**1**-4(NTf$_2$)]$^{4+}$, 1862.9 [**1**-5(NTf$_2$)]$^{3+}$.

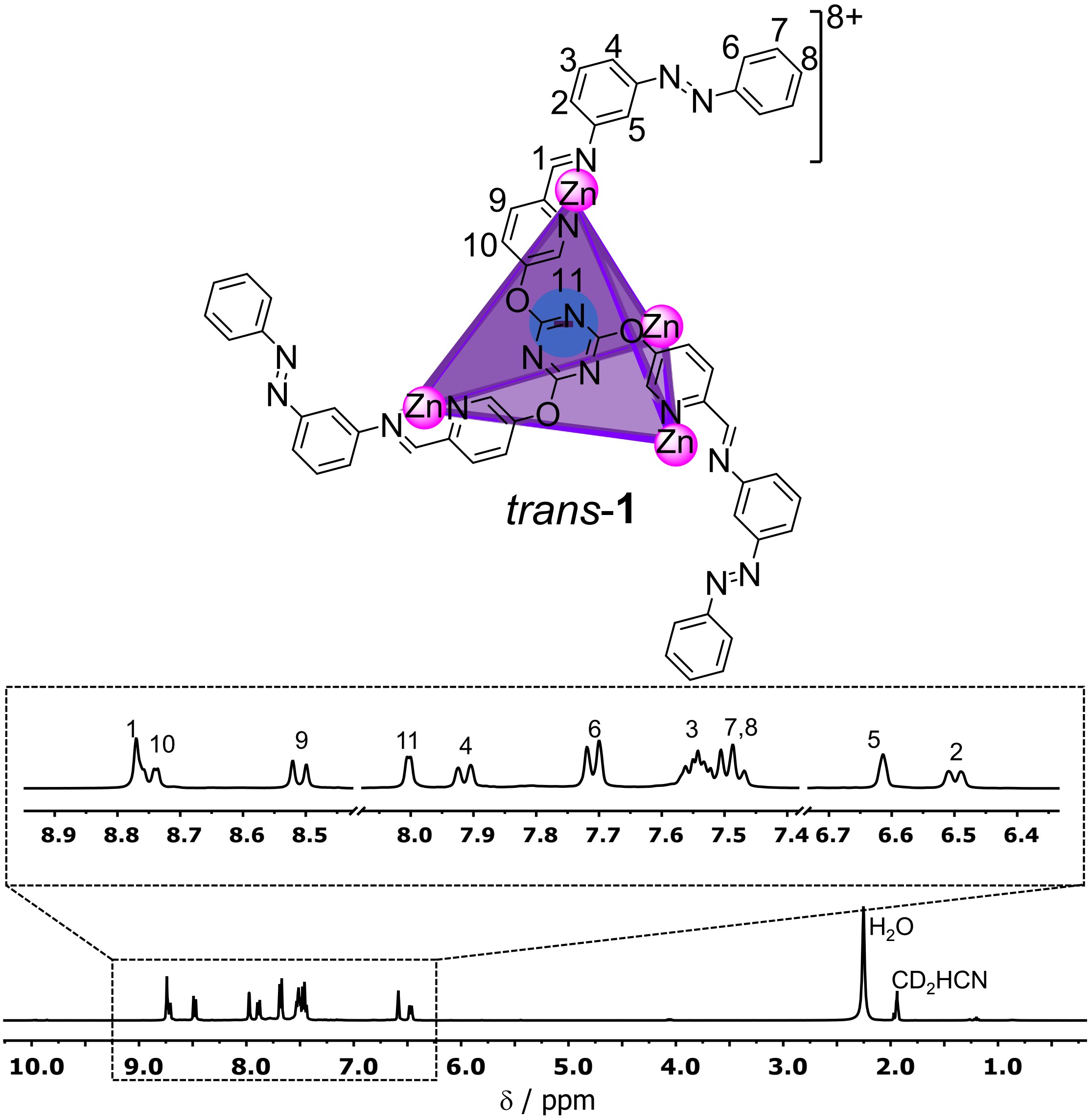

Figure S3. ^{1}H NMR spectrum of Tf_2N^{-}⊂*trans*-**1** (400 MHz, CD_3CN, 25 °C).

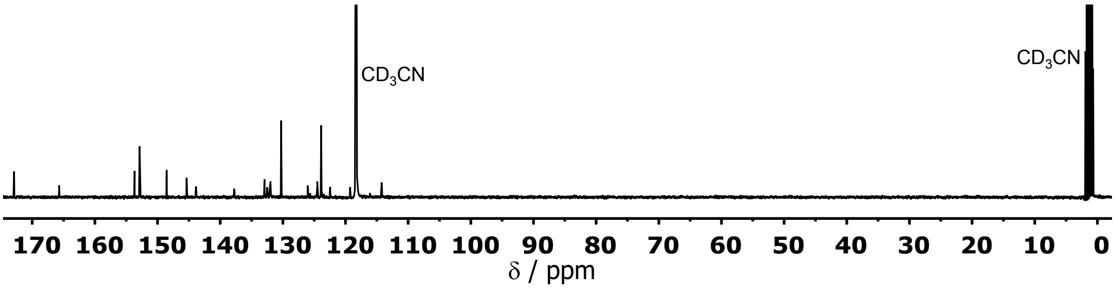

Figure S4. ^{13}C NMR spectrum of Tf_2N^{-}⊂*trans*-**1** (100 MHz, CD_3CN, 25 °C).

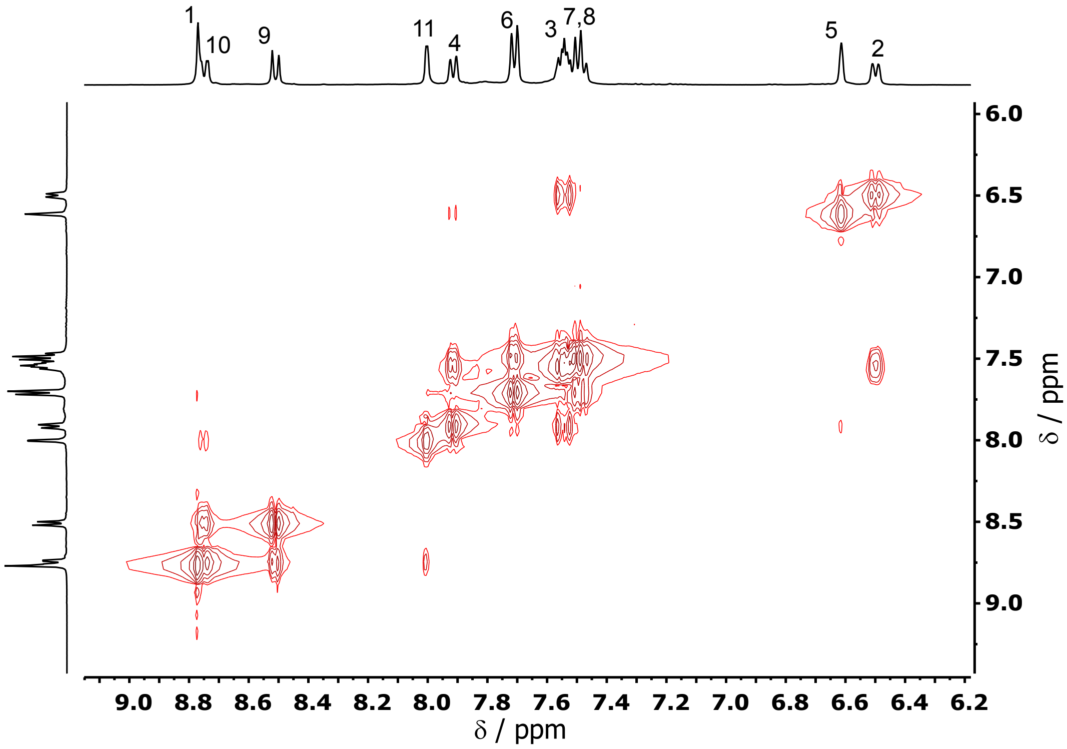

Figure S5. ^{1}H-^{1}H COSY NMR spectrum of $Tf_2N^{-}\subset$*trans*-**1** (400 MHz, CD_3CN, 25 °C).

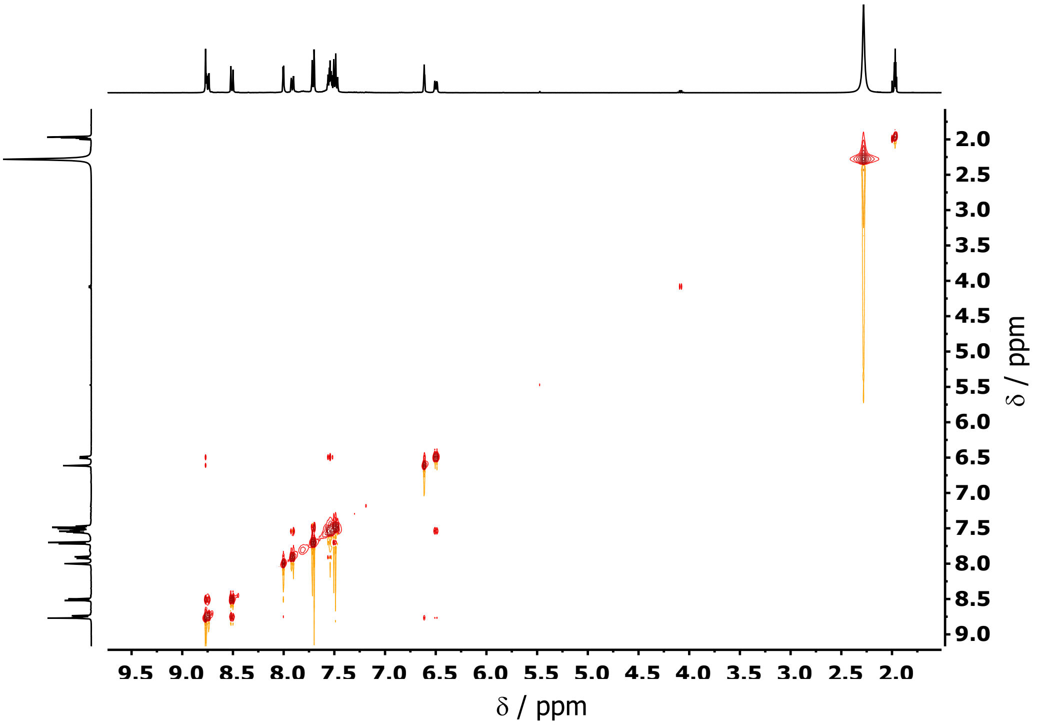

Figure S6. ^{1}H-^{1}H NOESY NMR spectrum of $Tf_2N^{-}\subset$*trans*-**1** (400 MHz, CD_3CN, 25 °C).

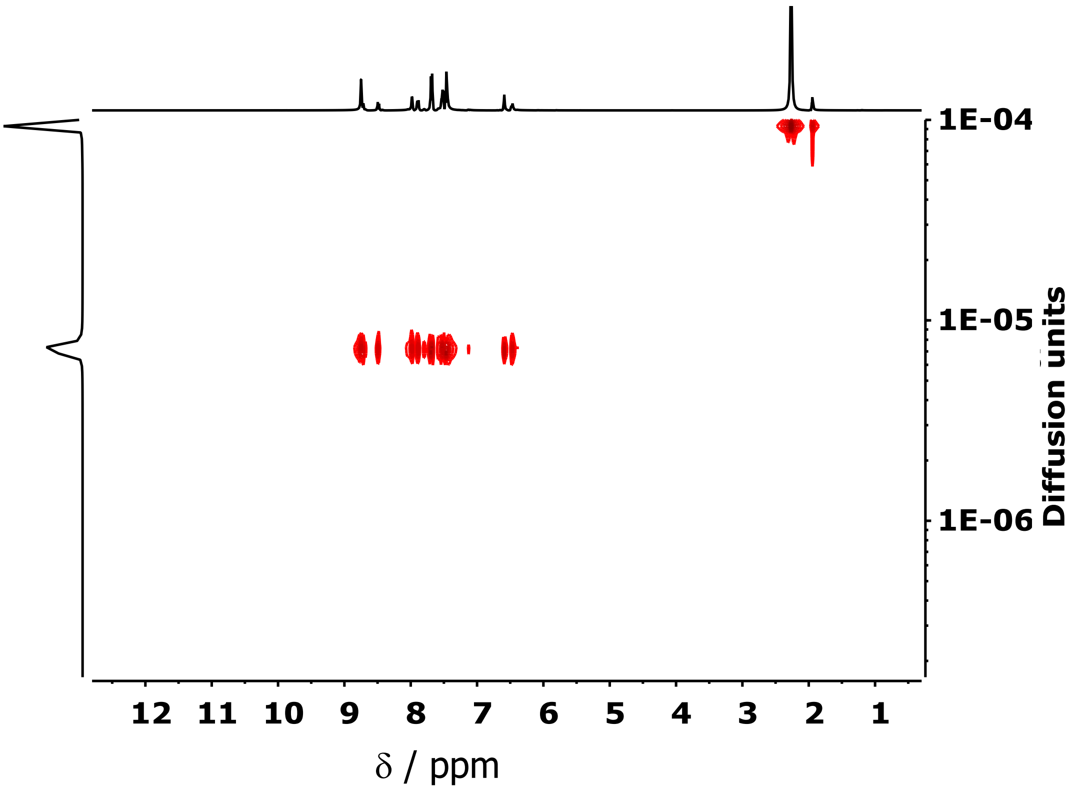

Figure S7. ^{1}H DOSY spectrum of $Tf_2N^{-}\subset$*trans*-**1** (400 MHz, CD_3CN, 25 °C). The diffusion coefficient in CD_3CN was measured to be 3.9×10^{-10} m^2/s.

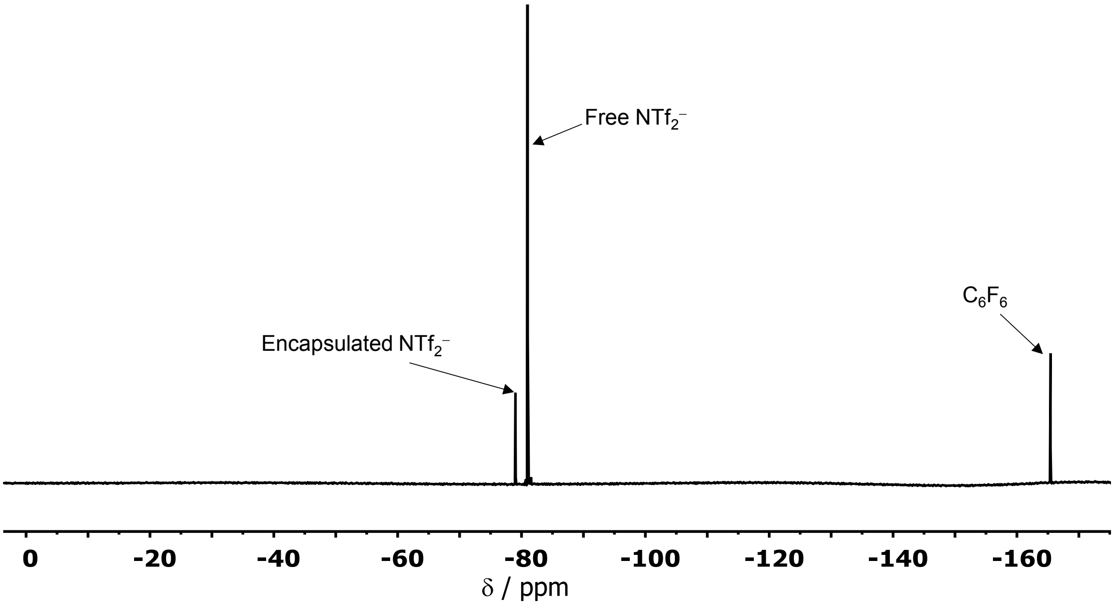

Figure S8. ^{19}F NMR spectrum of $Tf_2N^{-}\subset$*trans*-**1** (376 MHz, CD_3CN, 25 °C).

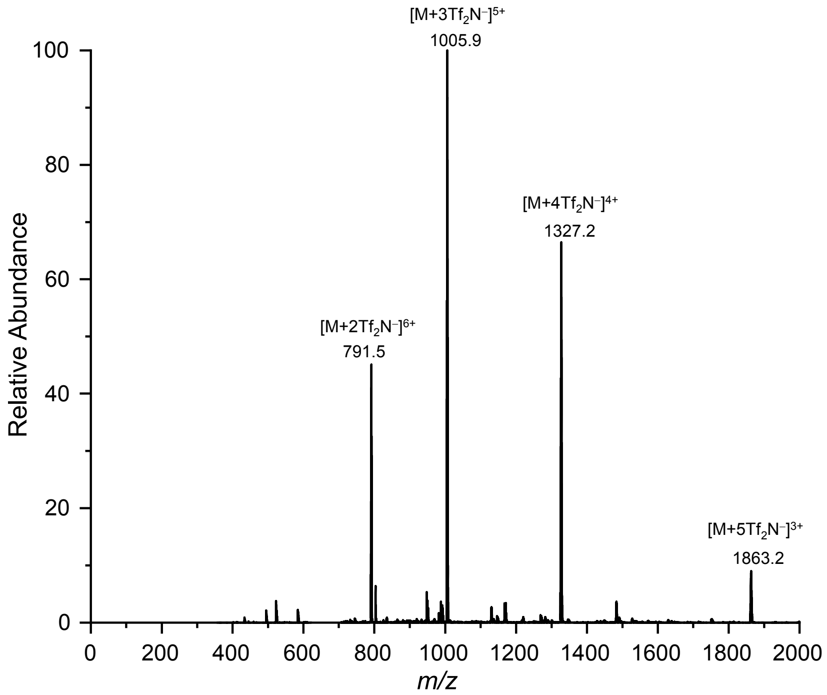

Figure S9. Low-resolution ESI-mass spectrum of Tf_2N^-⊂*trans*-**1** in CH_3CN.

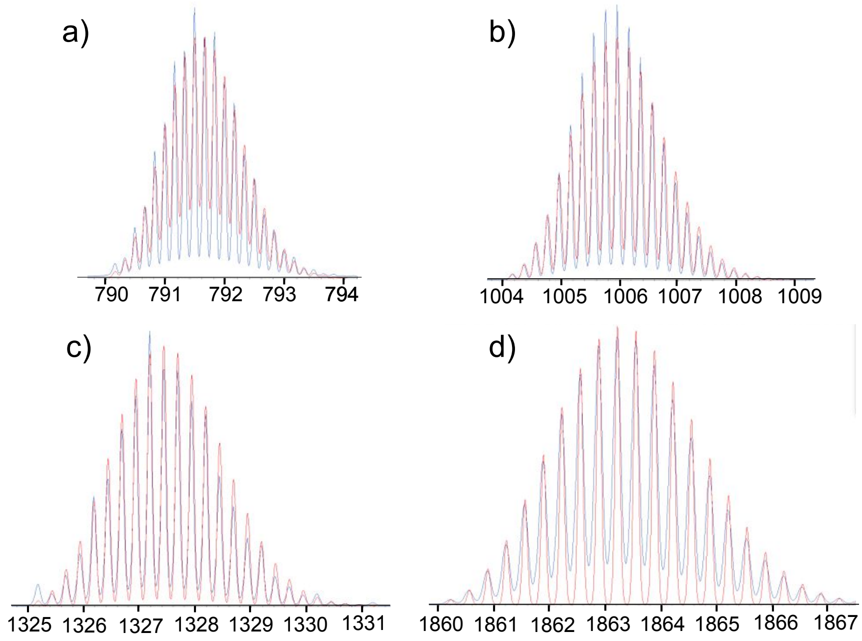

Figure S10. High-resolution ESI-MS spectra of Tf_2N^-⊂*trans*-**1** in CH_3CN. Experimental (blue) and calculated (red) peaks for a) [**1**-2(NTf_2)]$^{6+}$: *m/z* = 791.4812 (calculated *m/z* = 791.4824), b) [**1**-3(NTf_2)]$^{5+}$: *m/z* = 1005.7605 (calculated *m/z* = 1005.7624), c) [**1**-4(NTf_2)]$^{4+}$: *m/z* = 1327.1812 (calculated *m/z* = 1327.1823), d) [**1**-5(NTf_2)]$^{3+}$: *m/z* = 1862.8807 (calculated *m/z* = 1862.8822).

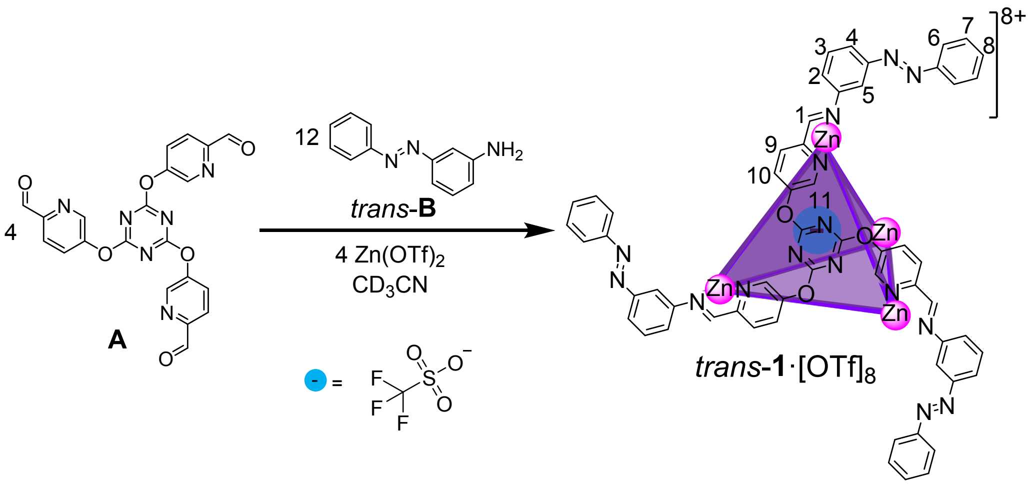

In an NMR tube, **A** (0.80 mg, 1.98 µmol, 4.0 equiv) in 500 µL of CD_3CN was combined with **B** (1.17 mg, 5.94 µmol, 12.0 equiv). To this mixture, $Zn(OTf)_2$ (0.72 mg, 1.98 µmol, 4.0 equiv) was added and subsequently the NMR spectrum was recorded. *Trans*-**1**·[OTf]$_8$ was obtained shown in Figure S11.

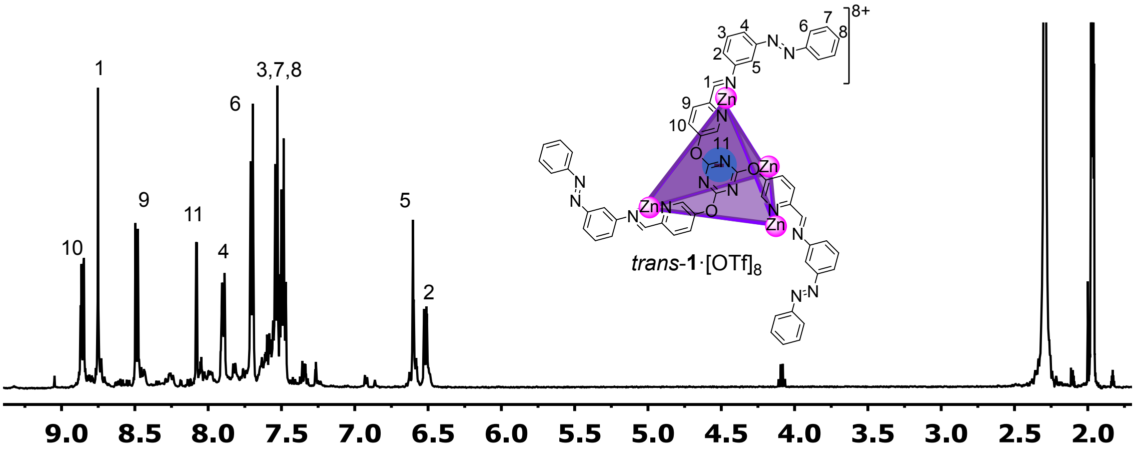

Figure S11. ^{1}H NMR spectrum of *trans*-**1**·[OTf]$_8$ (500 MHz, CD_3CN, 25 °C).

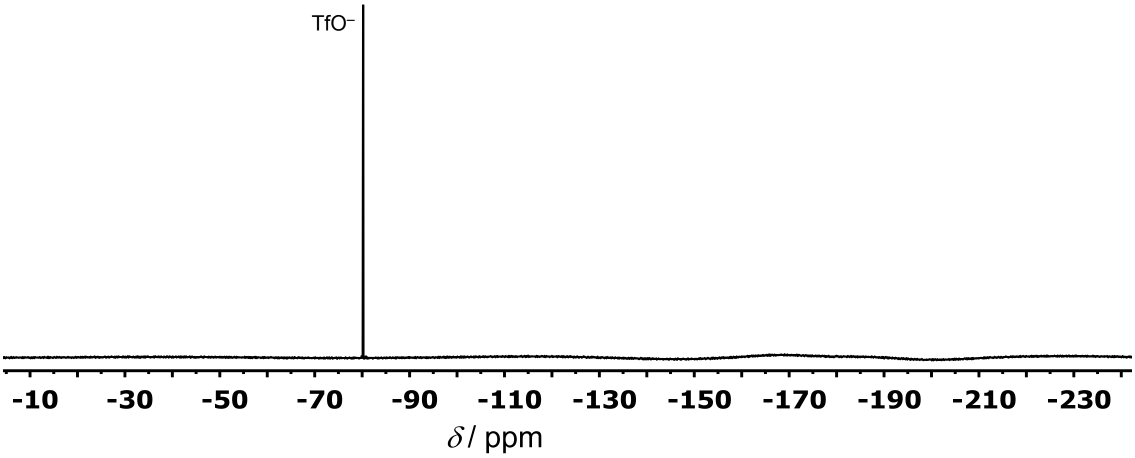

Figure S12. ^{19}F NMR spectrum of trans-**1**·[OTf]$_8$ (376 MHz, CD_3CN, 25 °C).

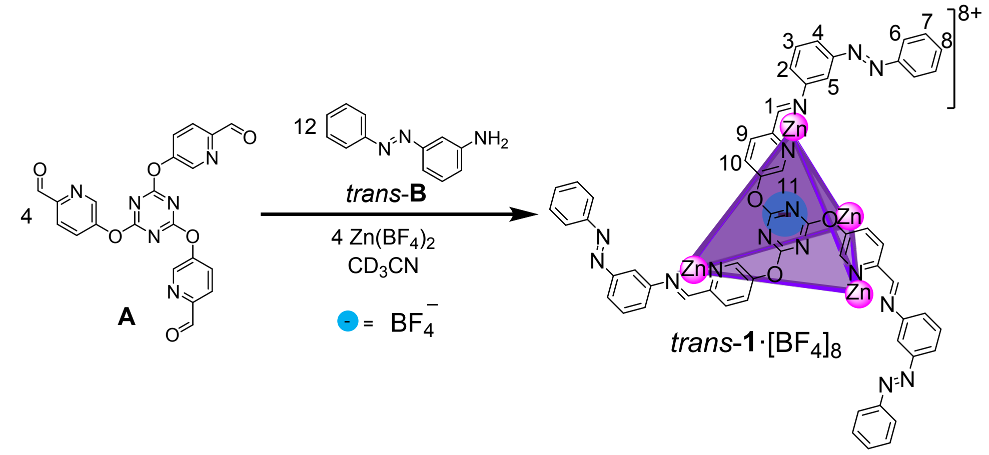

In an NMR tube, **A** (0.80 mg, 1.98 μmol, 4.0 equiv) in 500 μL of CD_3CN was combined with **B** (1.17 mg, 5.94 μmol, 12.0 equiv). To this mixture, $Zn(BF_4)_2$ (0.47 mg, 1.98 μmol, 4.0 equiv) was added and subsequently the NMR spectrum was recorded. *Trans*-**1**·$[BF_4]_8$ was obtained as major product.

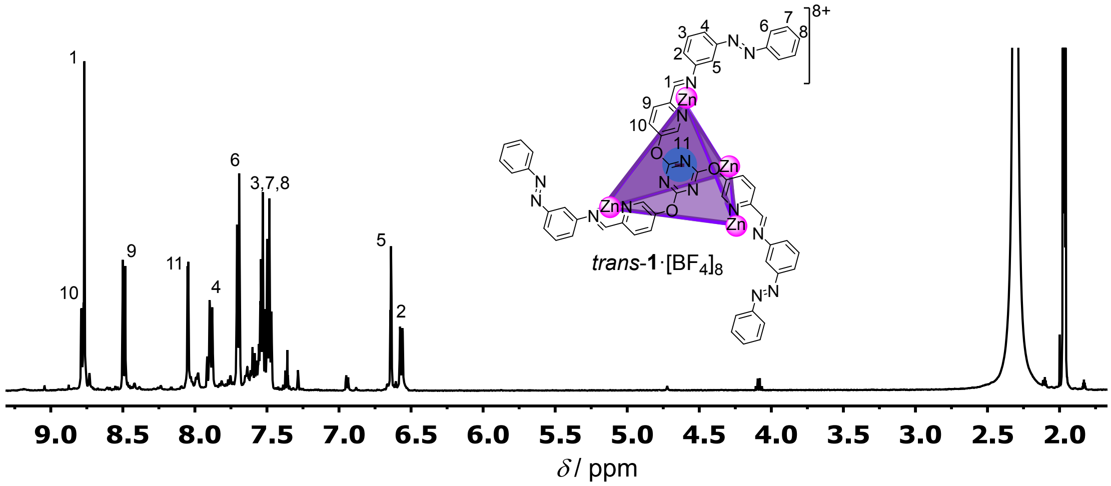

Figure S13. ^1H NMR spectrum of *trans*-**1**·$[BF_4]_8$ (500 MHz, CD_3CN, 25 °C).

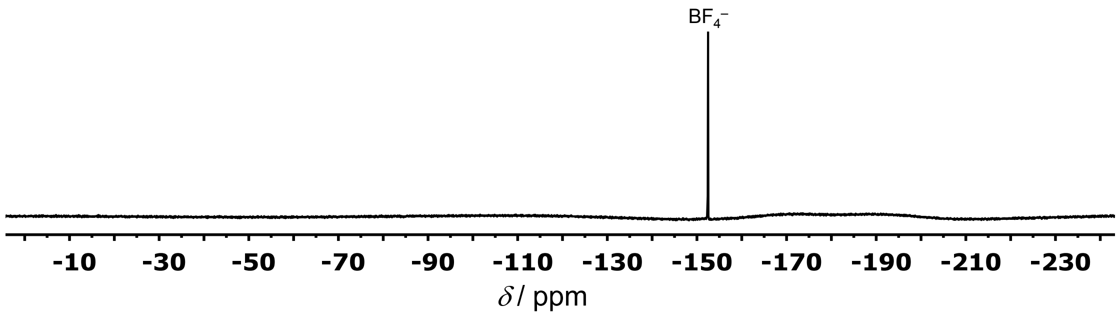

Figure S14. ^{19}F NMR spectrum of trans-**1**·$[BF_4]_8$ (376 MHz, CD_3CN, 25 °C).

S4 Self-Assembly and Characterization of $Tf_2N^- \subset 2$

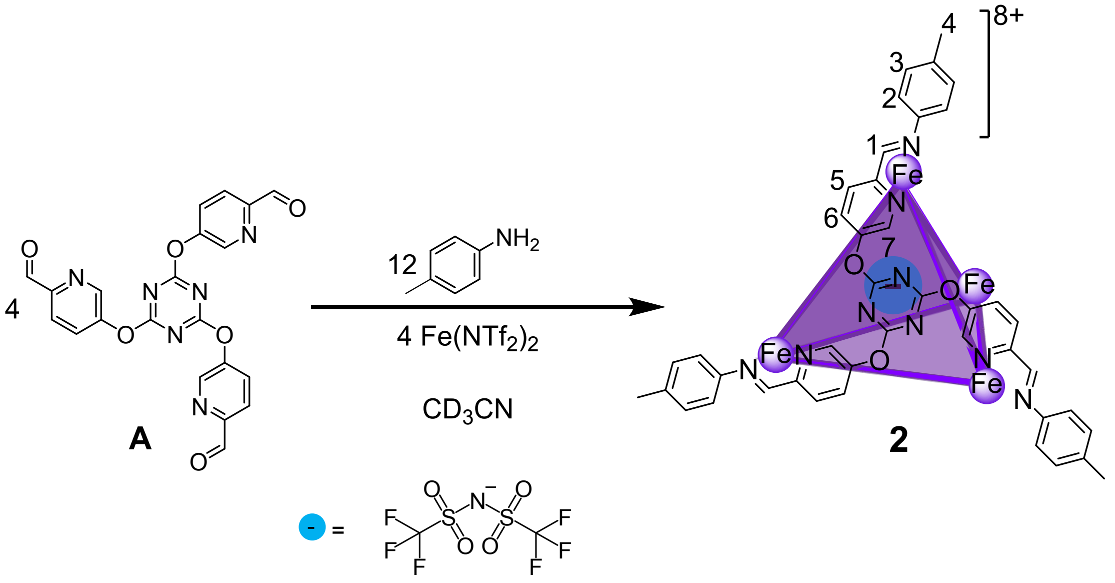

Subcomponent **A** (4.0 mg, 9.0 *μ*mol), *p*-toluidine, (2.9 mg, 27 *μ*mol), and iron(II) triflimide [$Fe(NTf_2)_2$] (5.5 mg, 9.0 *μ*mol) were loaded into a J-Young NMR tube and dissolved in CD_3CN (0.6 mL). The NMR tube was subjected to three evacuation/nitrogen fill cycles to remove oxygen, sealed and then heated for 24 h at 70 °C. The addition of diethyl ether to the resulting solution induced precipitation, the solid was isolated by centrifugation, dried under vacuum and redissolved in CD_3CN.

^{1}H NMR (400 MHz, CD_3CN): δ = 9.06 (s, 12H, 1-H $Tf_2N^- \subset$**2**), 8.86 (s, 12H, 1'-H **2**), 8.69 (d, *J* = 9.0 Hz, 12H, 5-H $Tf_2N^- \subset$**2**), 8.65 – 8.50 (m, 36H, overlapping 6-H $Tf_2N^- \subset$**2** + 5'-H **2** + 6'-H **2**), 7.52 (s, 12 H, 7-H $Tf_2N^- \subset$**2**), 7.38 (s, 12 H, 7'-H **2**), 7.07 (d, *J* = 7.4 Hz, 48H, overlapping 2-H $Tf_2N^- \subset$**2** + 2'-H **2**), 5.32 (d, *J* = 6.9 Hz, 48H, overlapping 3-H $Tf_2N^- \subset$**2** + 3'-H **2**), 2.36 (s, 72H, overlapping 4-H $Tf_2N^- \subset$**2** + 4'-H **2**) ppm. **^{19}F NMR** (376 MHz, CD_3CN): δ = –78.4 (s, *endo* Tf_2N^-), – 80.0 (s, *exo* Tf_2N^-) ppm. **ESI-MS**: *m/z* 478.6 [**2**-$(NTf_2)]^{7+}$, 605.0 [**2**-$2(NTf_2)]^{6+}$, 782.1 [**2**-$3(NTf_2)]^{5+}$, 1047.7 [**2**-$4(NTf_2)]^{4+}$, 1490.2 [**2**-$5(NTf_2)]^{3+}$. Crystals of $Tf_2N^- \subset$**2** were grown from slow diffusion of diethyl ether into a solution of the reaction mixture.

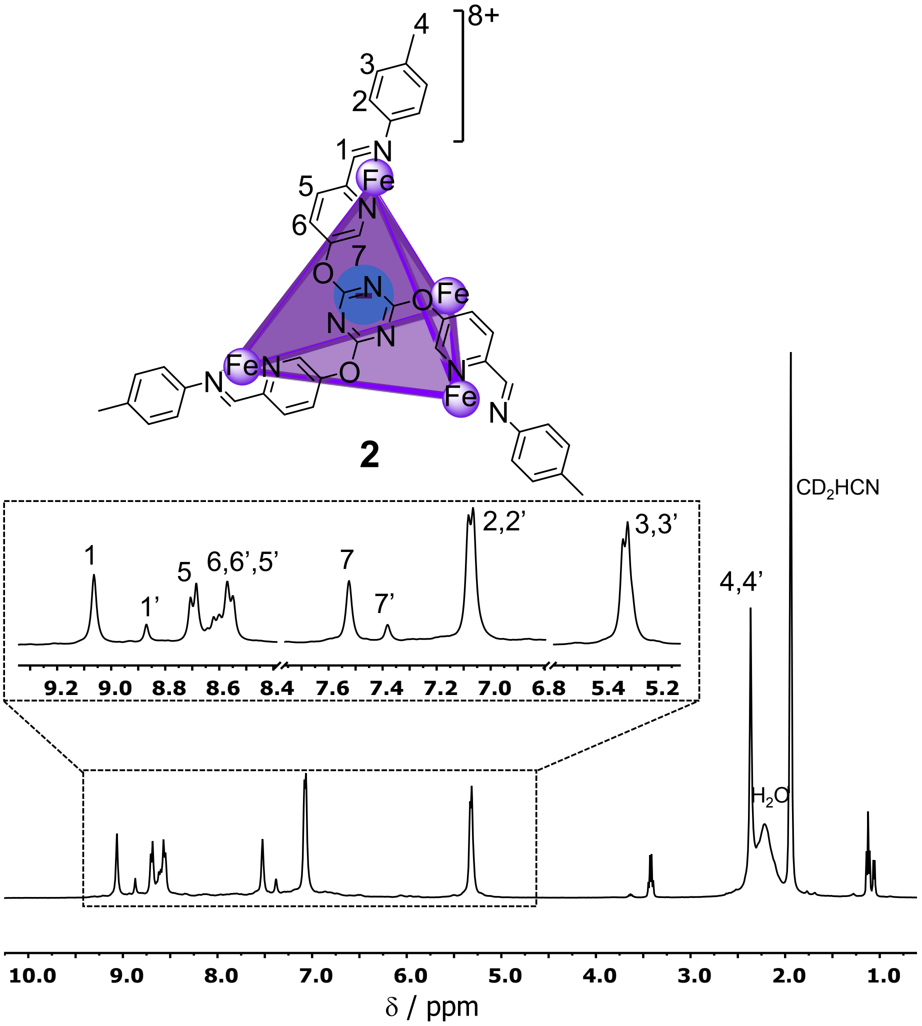

Figure S15. ^{1}H NMR spectrum of $Tf_2N^{-}\subset$**2** (400 MHz, CD_3CN, 25 °C).

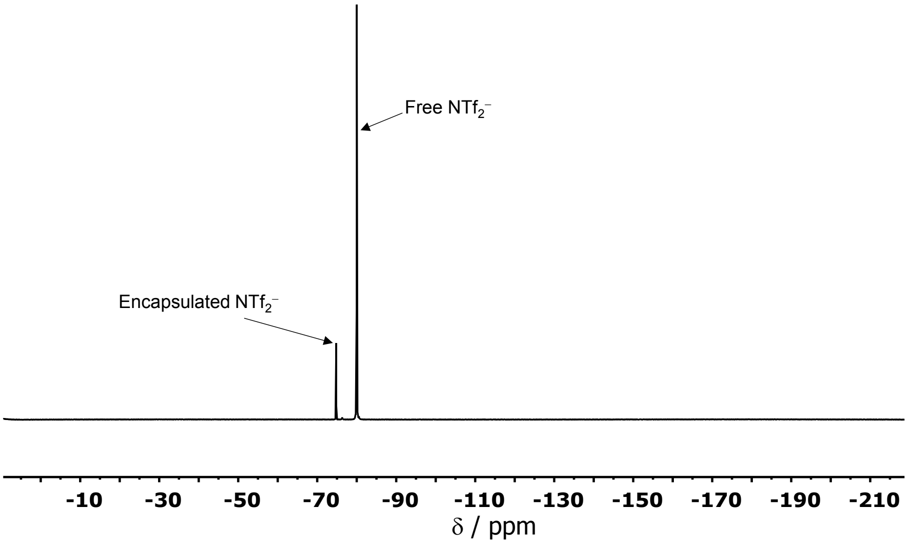

Figure S16. ^{19}F NMR spectrum of $Tf_2N^{-}\subset$**2** (376 MHz, CD_3CN, 25 °C).

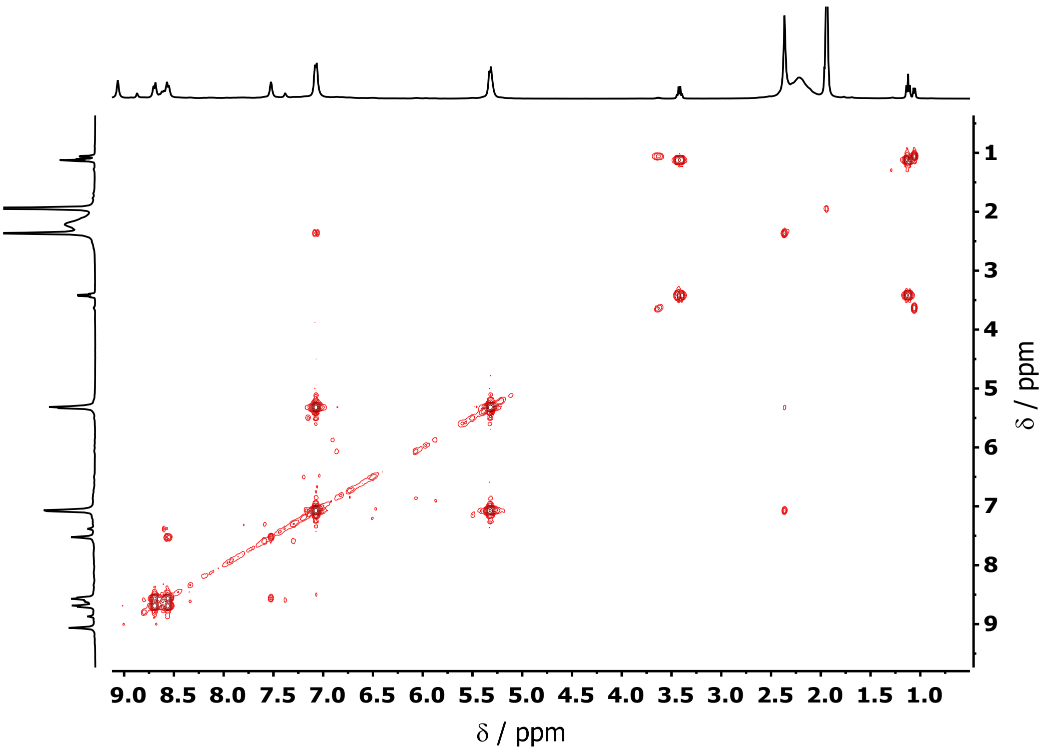

Figure S17. ^{1}H-^{1}H COSY NMR spectrum of $Tf_2N^- \subset$ **2** (400 MHz, CD_3CN, 25 °C).

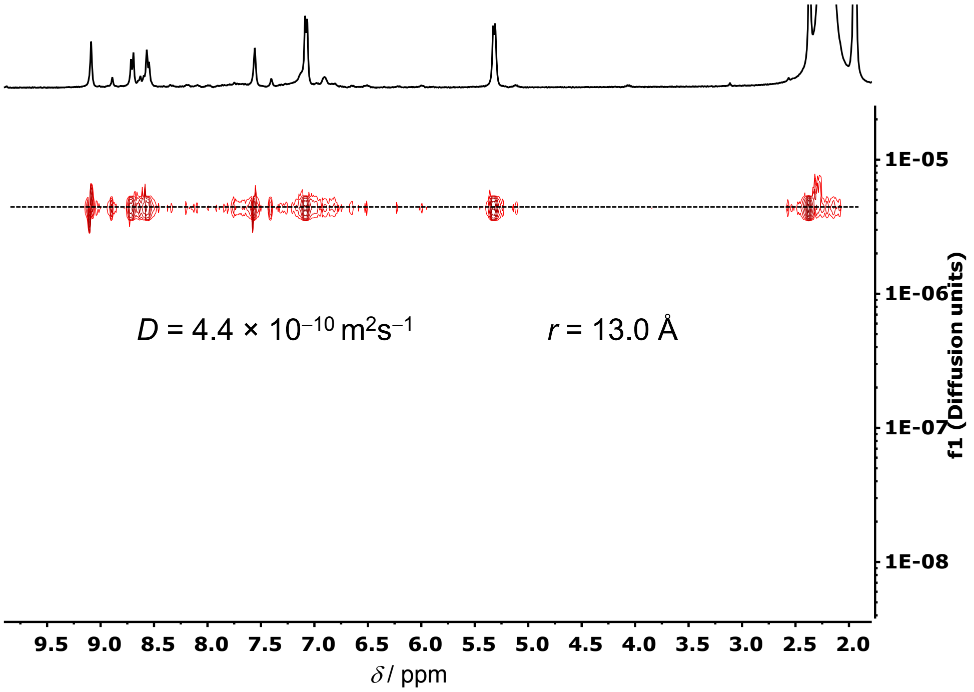

Figure S18. ^{1}H DOSY spectrum of $Tf_2N^- \subset$ **2** (400 MHz, CD_3CN, 25 °C).

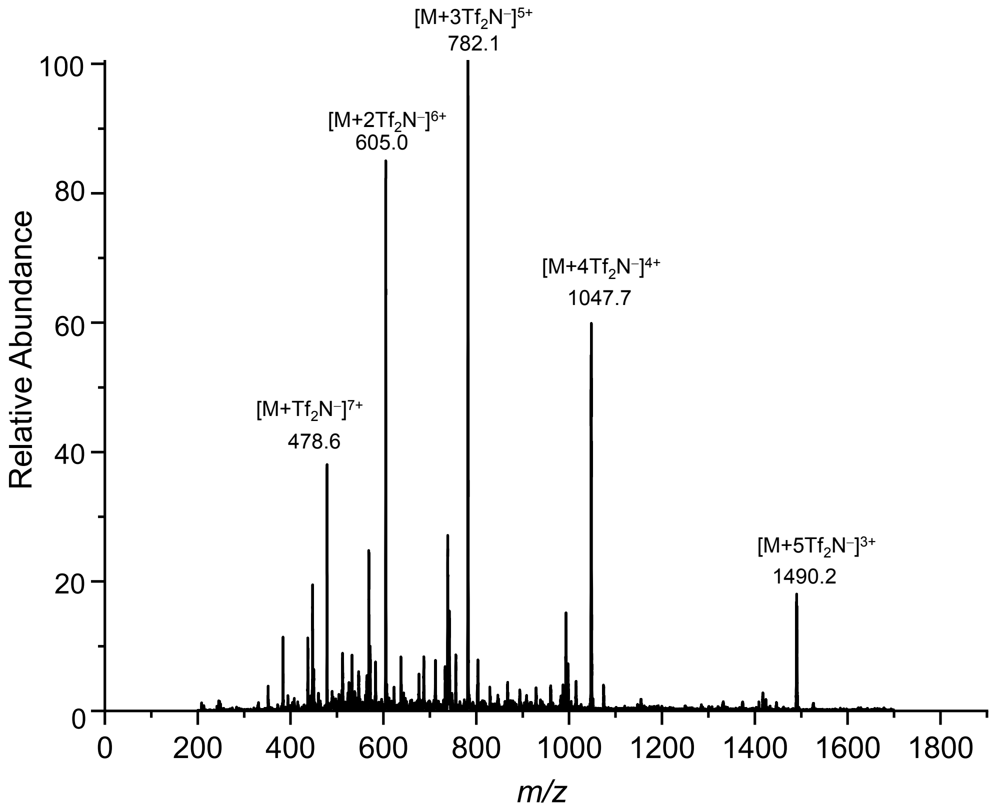

Figure S19. Low-resolution ESI-mass spectrum of $Tf_2N^-{\subset}\mathbf{2}$ in CH_3CN.

S5 Investigation of Photoswitching Properties of Subcomponent B

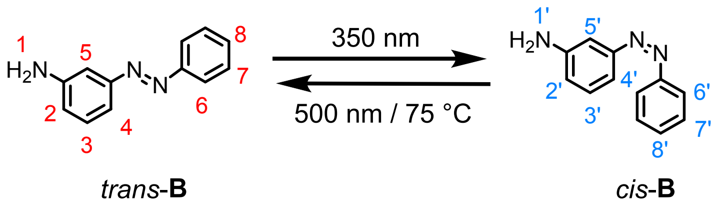

Figure S20. Reversible photoswitching of azobenzene-containing subcomponent **B.**

In an NMR tube, ligand *trans*-**B** (5.60 mg, 28.4 µmol) was dissolved in 500 µL of CD_3CN. An NMR spectrum was measured immediately after irradiation at 350 nm for 10 min. For reversible photoswitching, the same mixture was either irradiated at 500 nm (for 30 min) or heated at 75 °C (for 10 h).

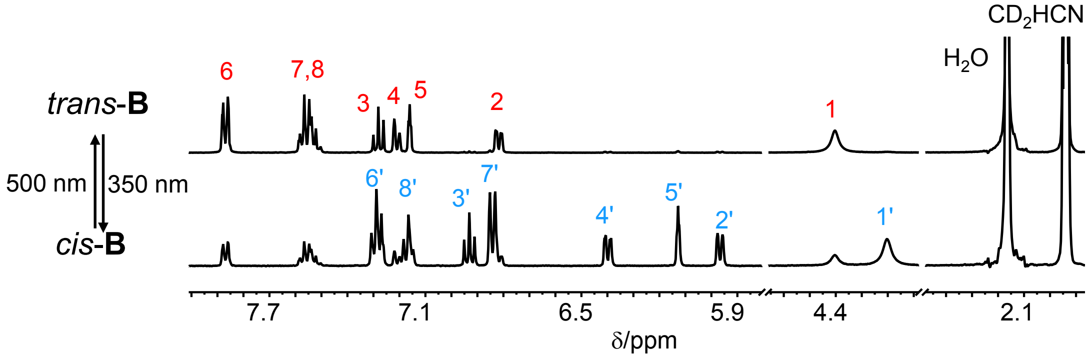

Figure S21. The comparison of ^1H NMR spectra of **B** (c = 56.8 mM) following exposure to light at 350 nm (10 min) and then at 500 nm (30 min) (400 MHz, CD_3CN, 25 °C). The protons of *trans*-**B** and *cis*-**B** have been assigned and are indicated by red and blue labels, respectively.

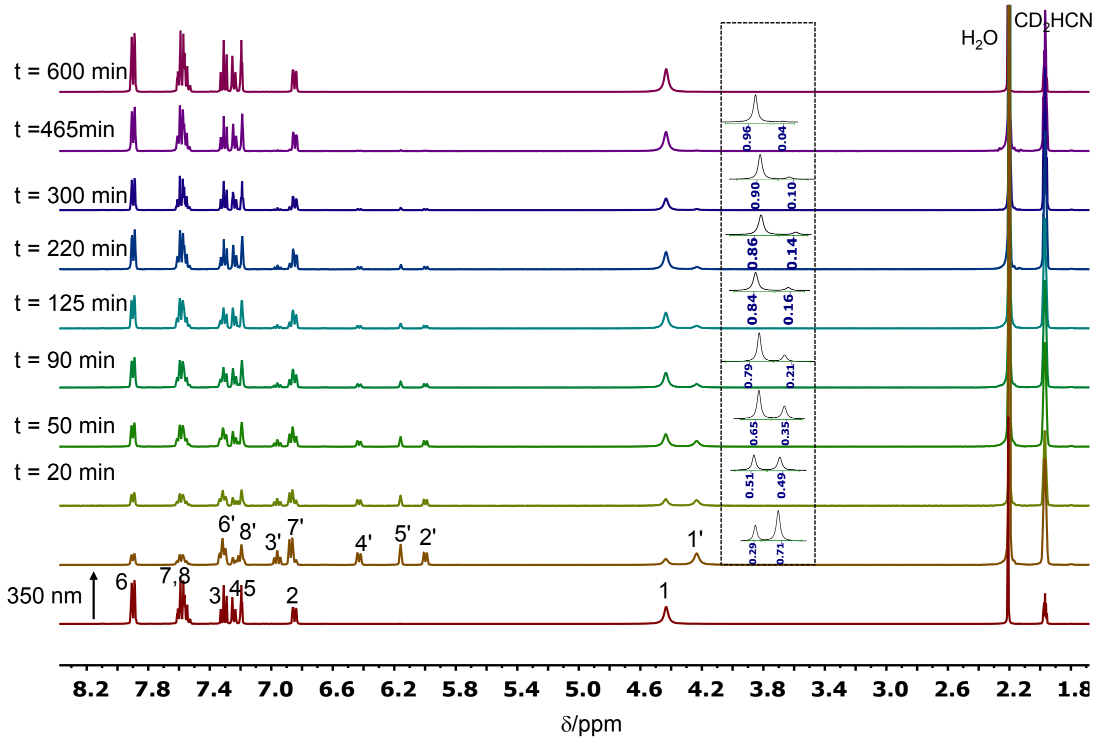

Figure S22. Time-dependent ^{1}H NMR (CD_3CN, 400 MHz, 25 °C) spectrum monitoring interconversion between *cis*-**B** to *trans*-**B** by heating the solution at 75 °C.

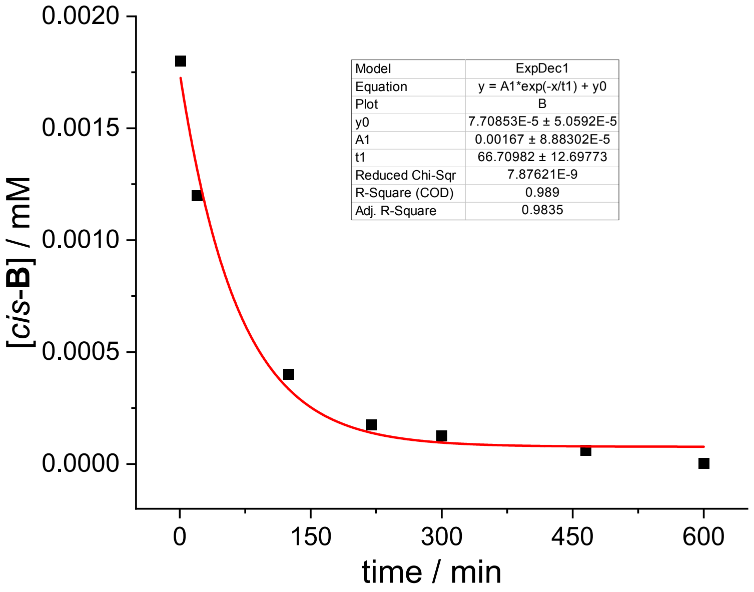

Figure S23. Kinetics for the *cis* to *trans* isomerisation of ligand **B** at 75 °C, monitored by the relative integration of protons 1 and 1' in the ^{1}H NMR (Figure S17). The fitting following a 1st order kinetics (red line), revealing a half-life of $t_{1/2}$ = 46 min and rate constants of k = 2.5 x 10^{-4} s^{-1}.

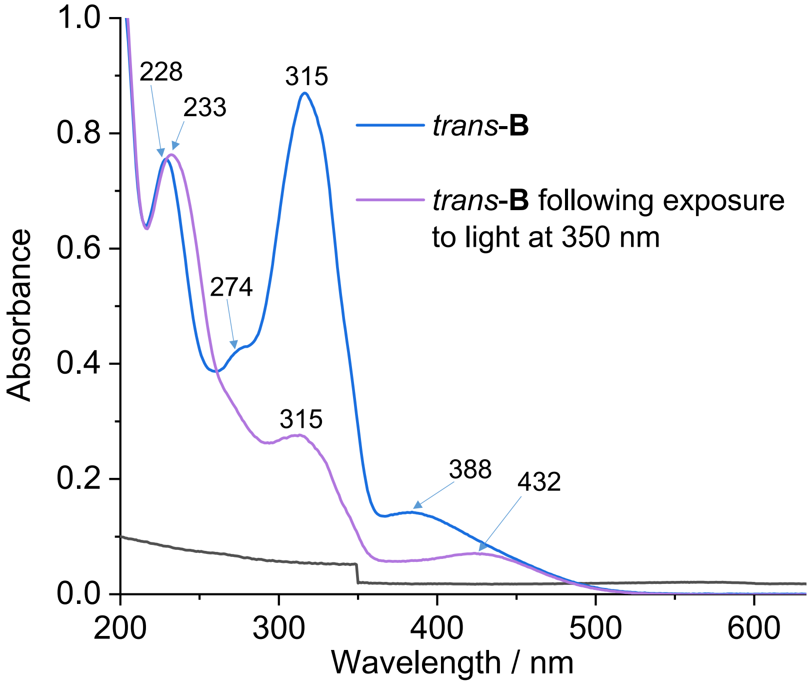

Figure S24. The comparison of the UV-vis spectra of *trans*-**B** (7.2×10^{-6} M, CH_3CN, 25 °C) and then following exposure to light at 350 nm for 10 min.

S6 Investigation of Photoswitching Properties of $Tf_2N^{-}\subset$*trans*-1

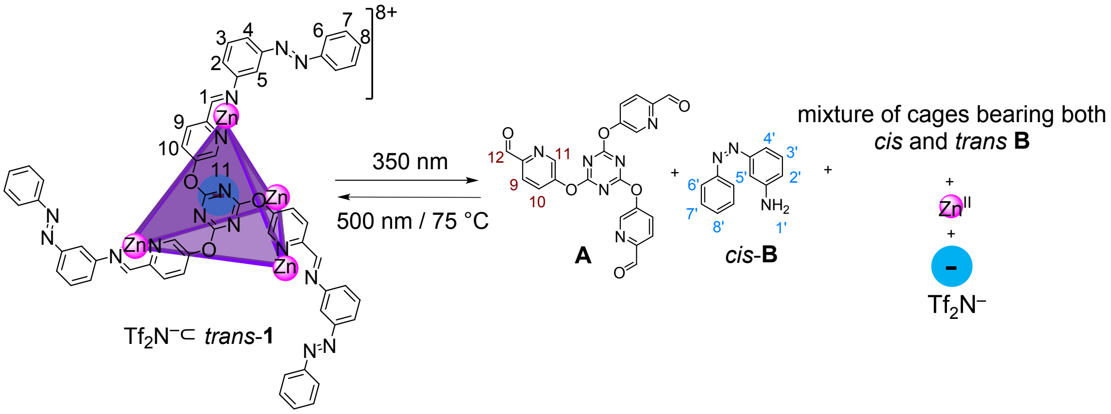

Figure S25. Disassembly and assembly of cage $Tf_2N^{-}\subset$*trans*-**1** and subsequent guest (NTf_2^-) release and uptake. A cartoon representation of the mixture of cages can be found in Figure S21.

In an NMR tube, **A** (8.80 mg, 19.8 μmol, 4.0 equiv) in 500 μL of CD_3CN was combined with *trans*-**B** (11.7 mg, 59.4 μmol, 12.0 equiv). To this mixture, $Zn(NTf_2)_2$ (12.4 mg, 19.8 μmol, 4.0 equiv) was added and the NMR tube was then irradiated at 350 nm. After 10 min the ^1H NMR spectrum was recorded. For the reverse process, the same

solution was irradiated at 500 nm for 30 min or heated to 75 °C for 10 h and the NMR spectrum was recorded subsequently.

S6.1 Characterization of Disassembly and Assembly of Tf_2N^-⊂*trans*-1 Using ^{1}H NMR

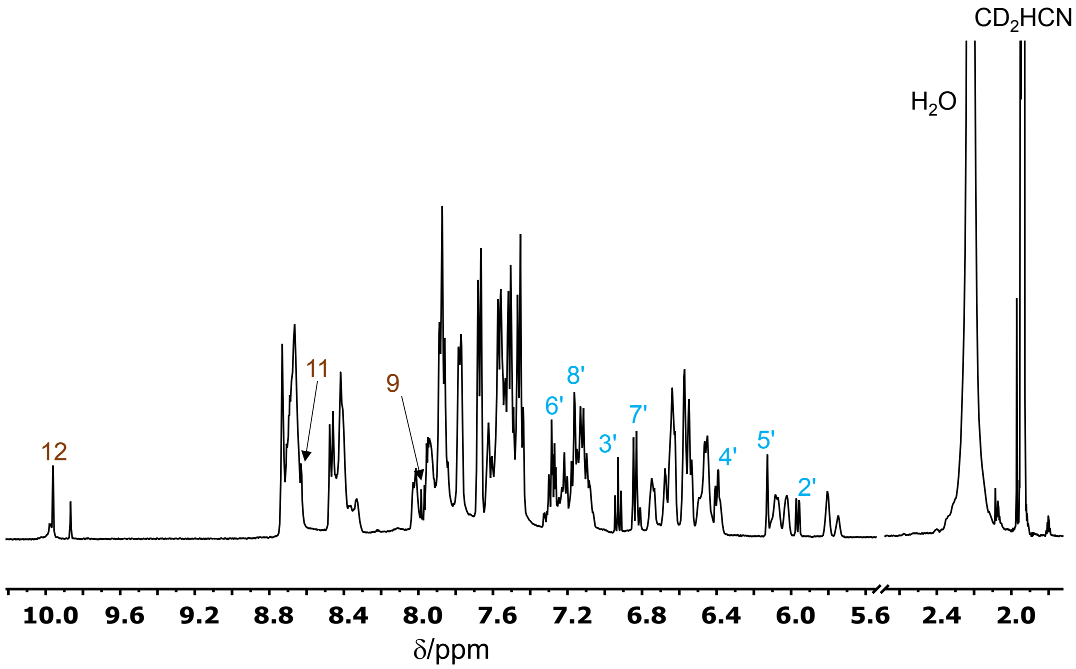

Figure S26. ^{1}H NMR spectra (500 MHz, CD_3CN, 25 °C) of cage Tf_2N^-⊂*trans*-**1** (*c* = 39.6 mM) after irradiation at 350 nm for 10 min. Only the signals for subcomponents **A** (brown) and *cis*-**B** (cyan) are marked.

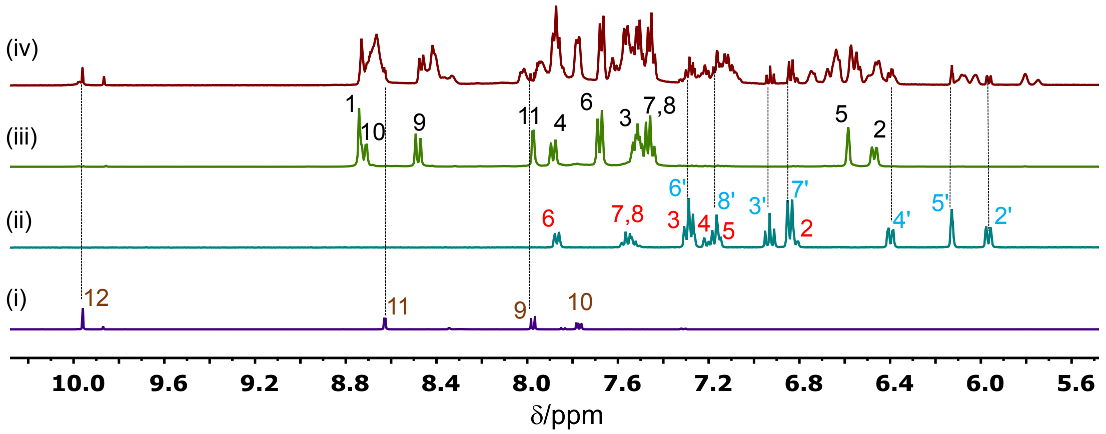

Figure S27. Comparison of ^{1}H NMR spectra (400 MHz, CD_3CN, 25 °C) of (i) subcomponent **A**; (ii) subcomponent **B** after 350 nm irradiation; (iii) pure cage Tf_2N^-⊂*trans*-**1** and (iv) cage Tf_2N^-⊂*trans*-**1** after 350 nm irradiation. The signals of subcomponent **A**, *trans*-**B**, *cis*-**B** and Tf_2N^-⊂*trans*-**1** have been assigned and are indicated by brown, red, cyan and black labels, respectively.

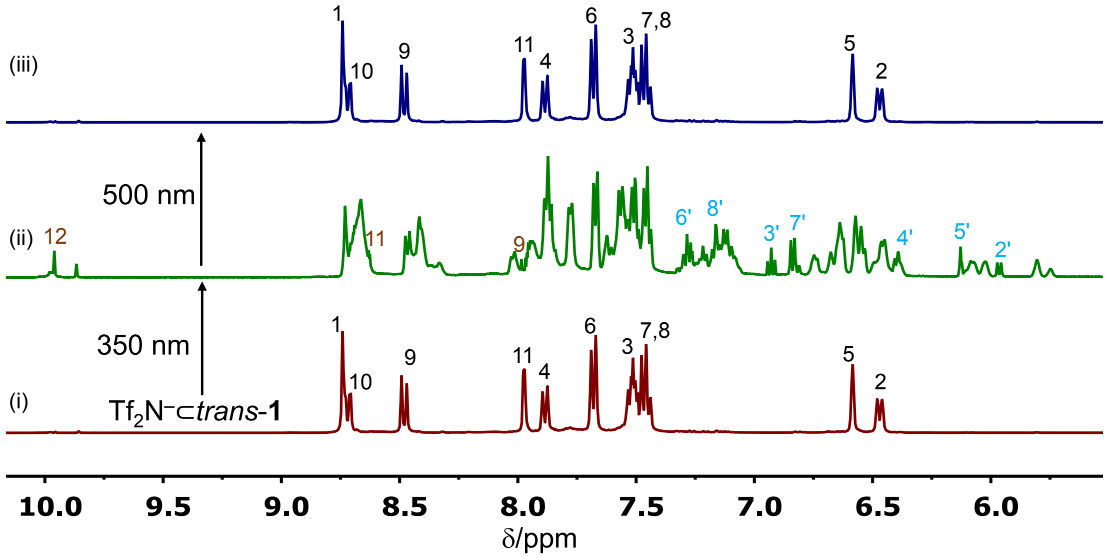

Figure S28. Reversible photoswitching of cage $Tf_2N^-\subset$*trans*-**1** (*c* = **3**9.6 mM). ^{1}H NMR spectra (500 MHz, CD_3CN, 25 °C) of (i) cage $Tf_2N^-\subset$*trans*-**1**; (ii) after 350 nm irradiation for 10 min and (iii) after irradiation at 500 nm for 30 min.

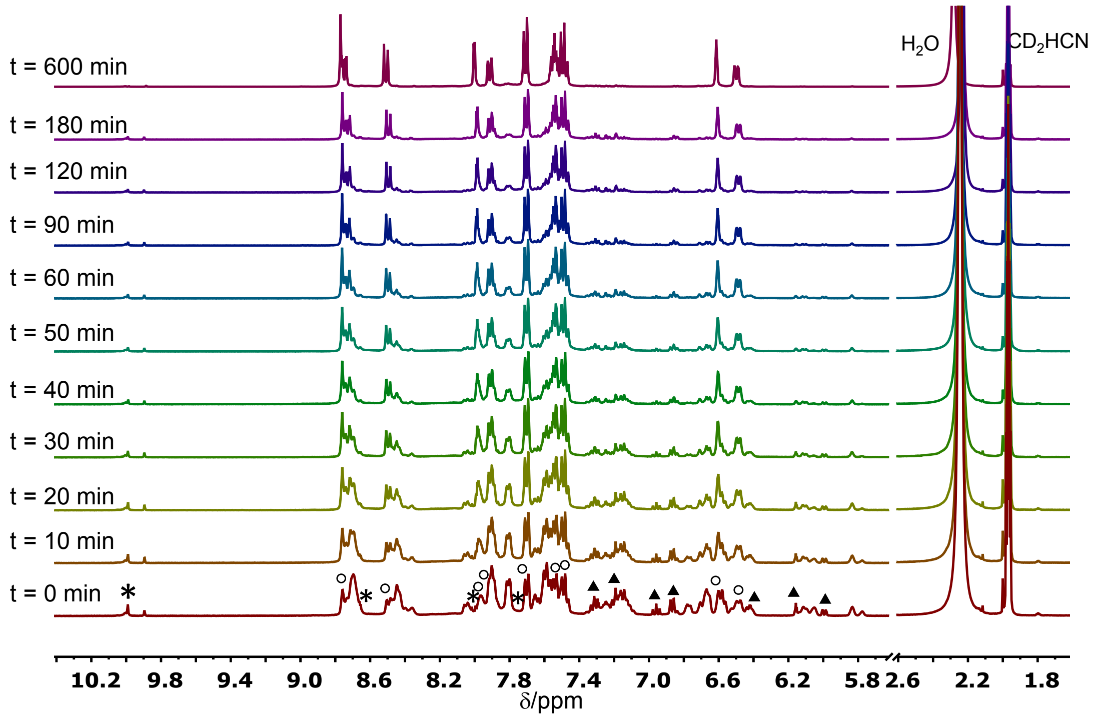

Figure S29. ^{1}H NMR spectra (400 MHz, CD_3CN, 25 °C) of cage $Tf_2N^-\subset$*trans*-**1** (*c* = 39.6 mM) after initial 350 nm irradiation, following the course of cage reassembly at 75 °C. Marked signals are assigned to protons using the following symbols: ○, Tf_2N^- $\subset$*trans*-**1**; ▲, *cis*-**B**; and *, subcomponent **A**.

S6.2 Characterization of Guest (Tf_2N^-) Release and Uptake Using ^{19}F NMR

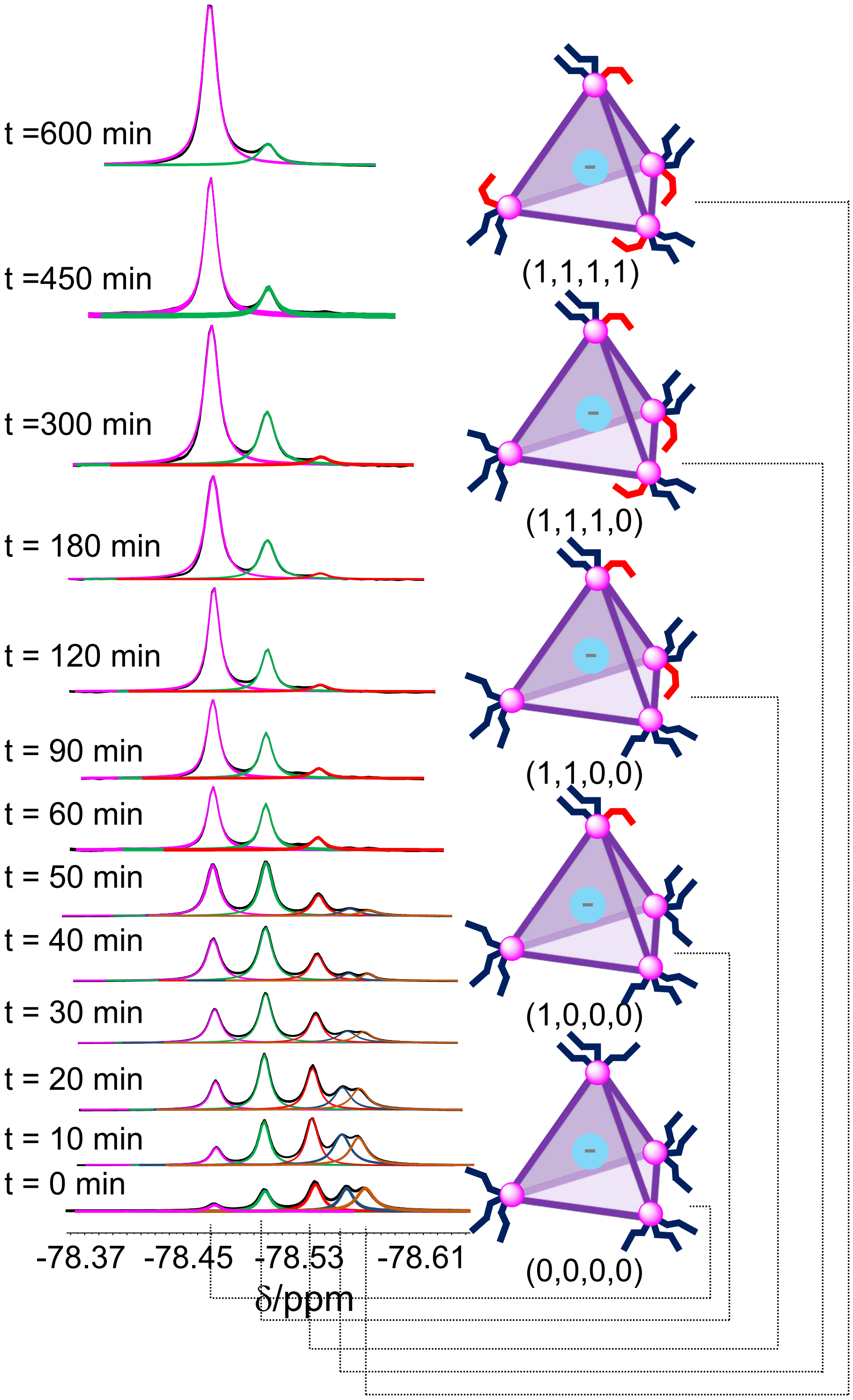

Figure S30. ^{19}F NMR spectra (376 MHz, CD_3CN, 25 °C) of Tf_2N^-⊂*trans*-**1** (39.6 mM) at the indicated times during 75 °C heating after 350 nm UV irradiation. Deconvolution of peak clusters with calculated Lorentzian peaks are color coded. The peak at –78.47 (magenta), -78.51 (green), -78.54 (red), -78.57 (blue), and -78.58 (brown) represent Tf_2N^- encapsulated within cages incorporating 0, 1, 2, 3, and 4 *cis* diazo moieties, respectively. The speciation profile in Figure 5 was extracted from this experiment.

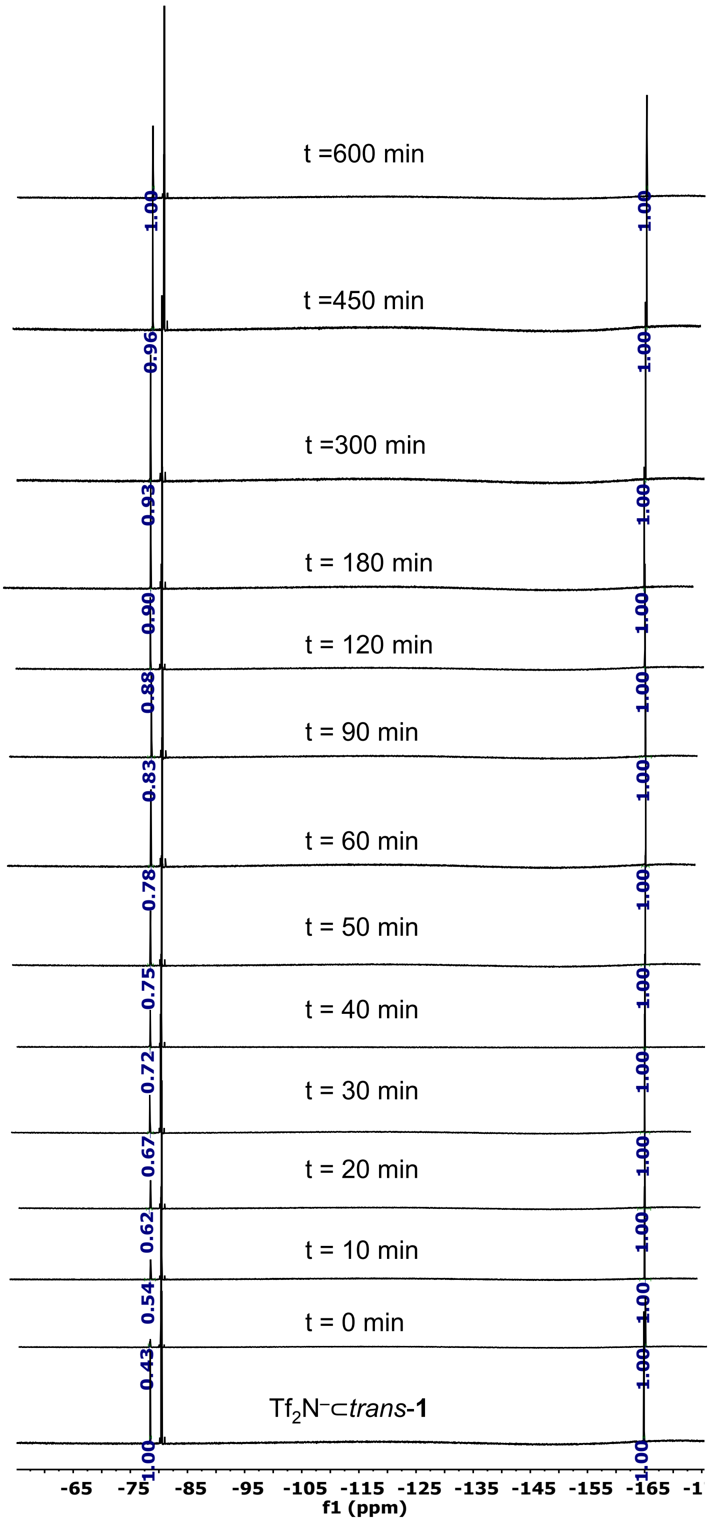

Figure S31. ^{19}F NMR spectra (376 MHz, CD_3CN, 25 °C) of cage Tf_2N^-⊂*trans*-**1** (*c* = 39.6 mM) at various times recorded with heating at 75 °C after 350 nm UV irradiation. t = 0 min is after stopping irradiation (when the guest has been released). The integration of peaks at -78.47 to -78.58 ppm with respect to hexafluorobenzene shows gradual uptake of guest Tf_2N^- by the cage *trans*-**1**. The first cycle of Figure 6c is plotted from this experiment.

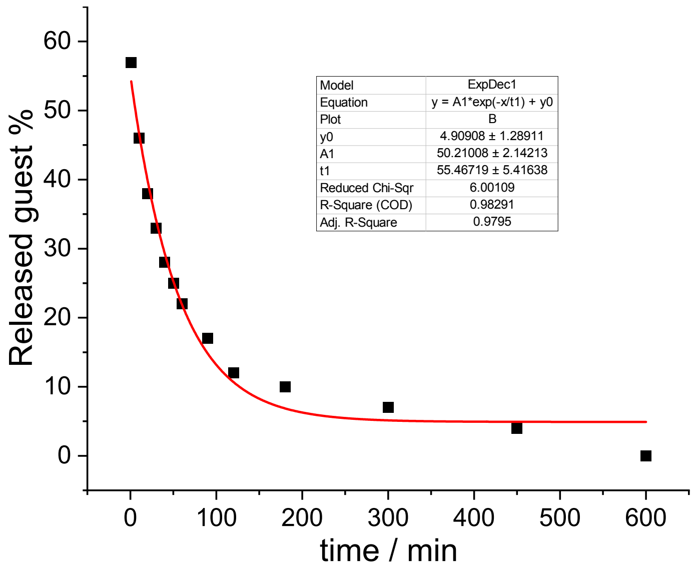

Figure S32. Kinetics for the guest uptake process during reverse photoisomerization towards *trans*-**1** at 75 °C, monitored by integration of encapsulated Tf_2N^- with respect to hexafluorobenzene (Figure S27). The fitting following a 1st order kinetics (red line), revealing a half-life of $t_{1/2}$ = 38 min and rate constants of k = 3.50 x 10^{-4} s^{-1}.

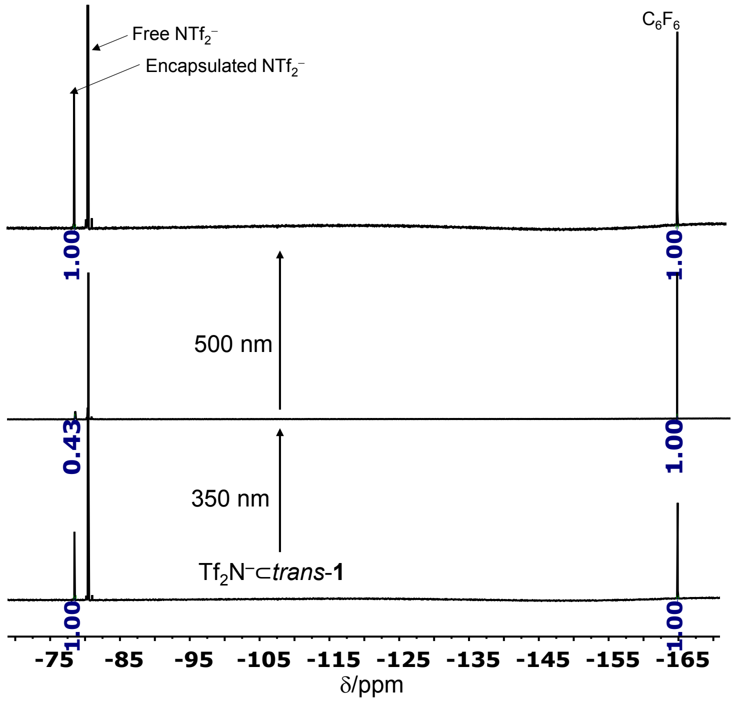

Figure S33. ^{19}F NMR spectra (376 MHz, CD_3CN, 25 °C) of photoswitching of Tf_2N^- ⊂*trans*-**1** (c = 39.6 mM) shows reversible release and uptake of Tf_2N^-; integration of encapsulated Tf_2N^- is measured with respect to hexafluorobenzene as internal standard.

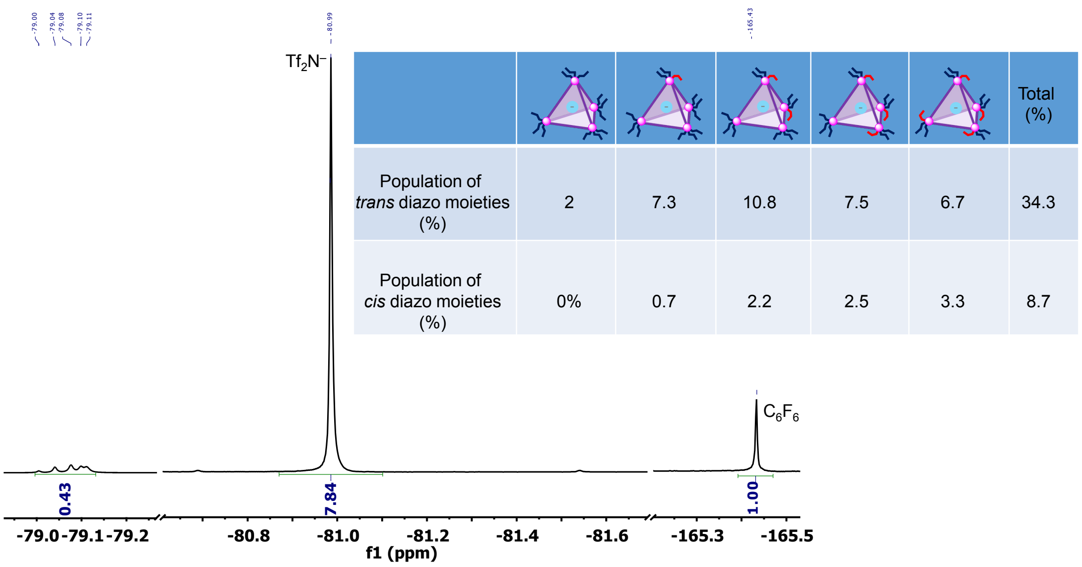

						Total (%)
Population of *trans* diazo moieties (%)	2	7.3	10.8	7.5	6.7	34.3
Population of *cis* diazo moieties (%)	0%	0.7	2.2	2.5	3.3	8.7

Figure S34. Population of *trans* (34.3%) and *cis* (8.7%) diazo moieties in the 43% of intact, triflimide-binding cages following UV irradiation. After UV irradiation, a total of six peaks appear in the ^{19}F NMR, with five corresponding to bound Tf_2N^- (43%) and one corresponding to free Tf_2N^- (57% released from a cage, along with the unbound counter anions). This 43% / 57% split thus corresponds to the proportion of ligand bound up into cages, vs. free ligand corresponding to cages that have opened. From the 43% of ligand incorporated into cages, we can calculate the amount of isomerization (34.3% *trans* and 8.7% *cis*), but we were unable to quantify the degree of isomerization among the other 57%.

S6.3 Characterization of Photo-switching of *trans*-1 Using UV/Vis Spectra

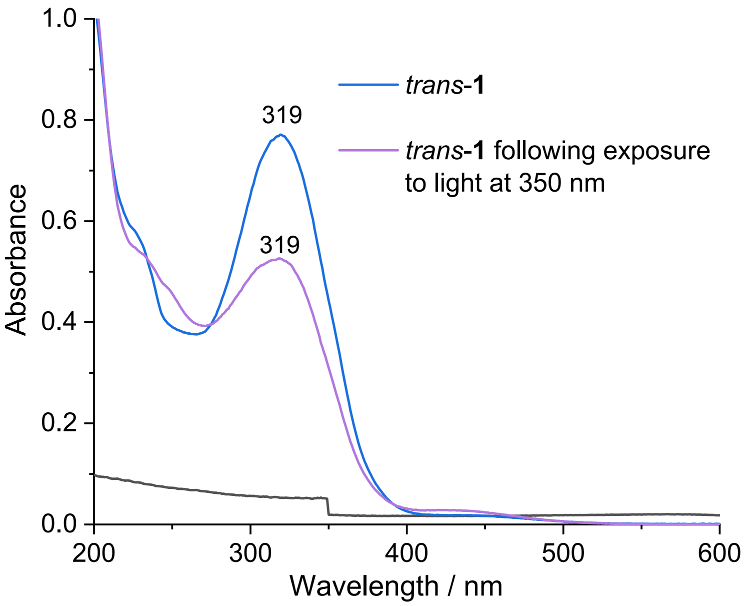

Figure S35. The comparison of the UV-vis spectra of *trans*-**1** (5.6×10^{-6} M, CH_3CN, 25 °C) and then following exposure to light at 350 nm for 10 min.

S7 Host-Guest Studies Using Tf_2N^{-}⊂*trans*-1

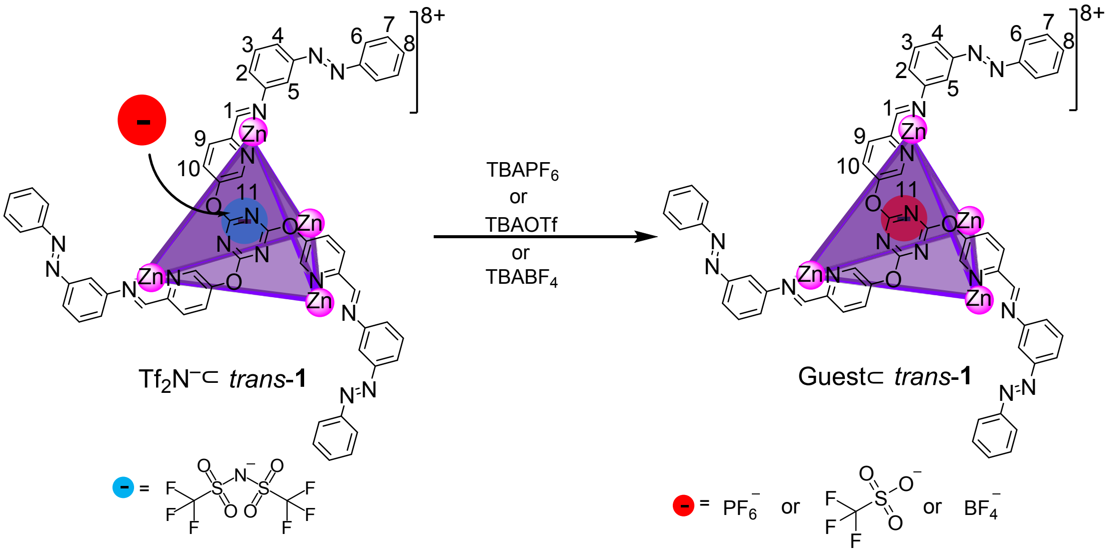

Figure S36. Encapsulation of other anionic guests (PF_6^-, TfO^- and BF_4^-) with subsequent release of Tf_2N^-.

The anions PF_6^- ($TBAPF_6$, where TBA is tetrabutylammonium), TfO^- (TBAOTf), and BF_4^- ($TBABF_4$) were investigated as potential guests for the **1**. Initially Tf_2N^-⊂*trans*-**1** was prepared in a NMR tube as described above. Five equivalents of the TBA salt of the relevant anion were then added progressively, one equivalent at a time, as a solid. ^1H and ^{19}F NMR spectra were recorded 30 minutes after each addition. It was found that all the guests were encapsulated by *trans*-**1**, showing a slow anion exchange on the ^1H NMR chemical shift time scale.

Anion	Volume (Å^3)
Tf_2N^-	131
PF_6^-	90
TfO^-	86
BF_4^-	50

Table S1. Reported anion volumes.[2,3]

S7.1 Encapsulation of PF_6^-

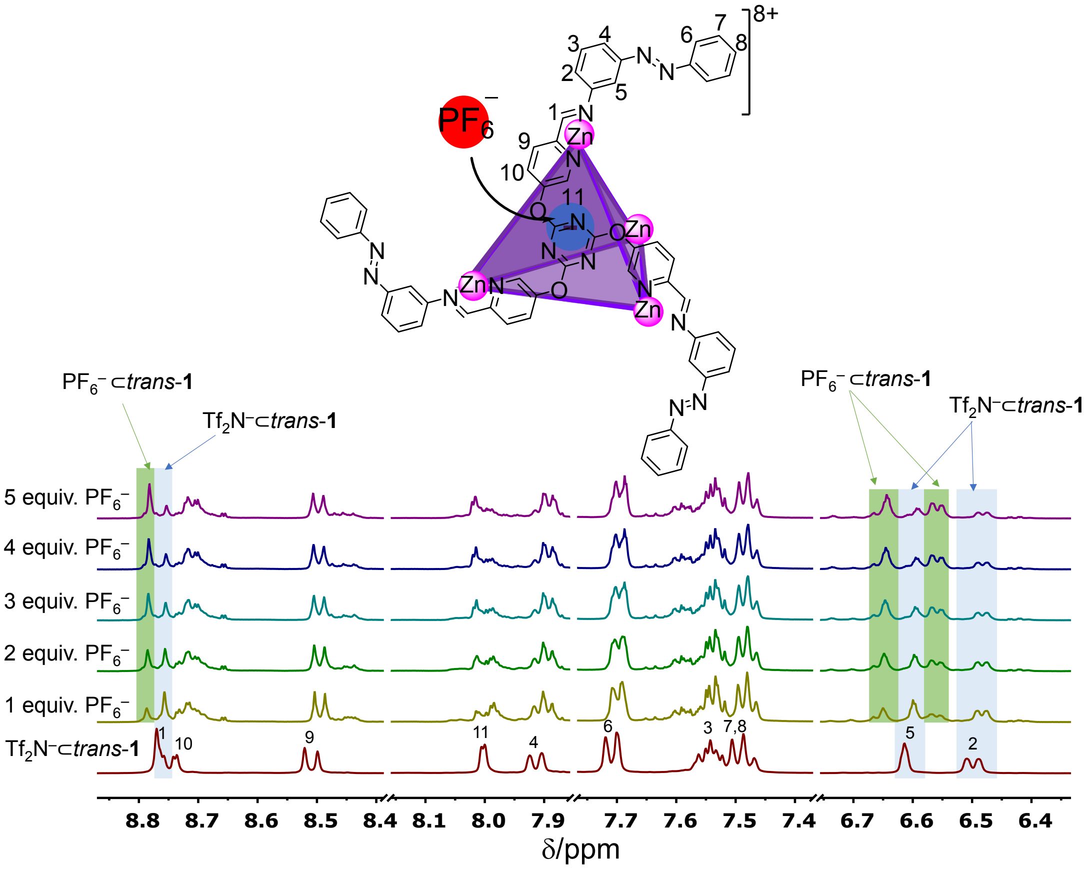

Figure S37. ^{1}H NMR (CD_3CN, 500 MHz, 25 °C) titration of PF_6^-into a solution of Tf_2N^- ⊂*trans*-**1** (9.9 mM).

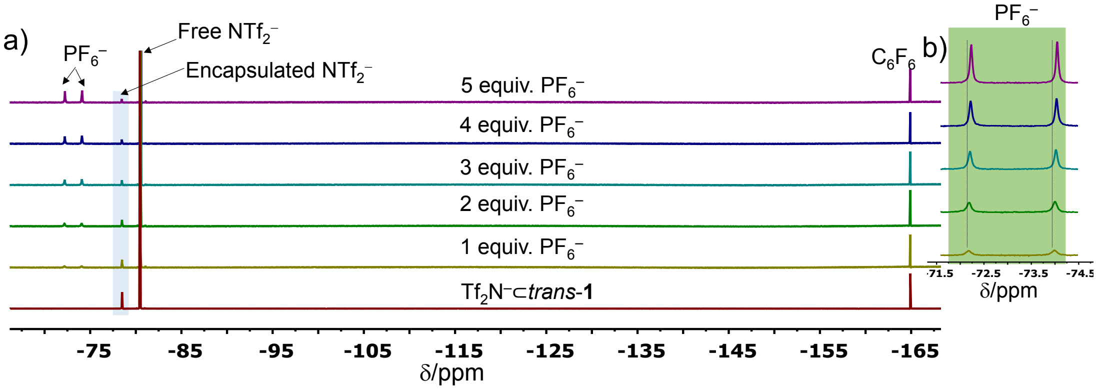

Figure S38. a) ^{19}F NMR (CD_3CN, 376 MHz, 25 °C) titrations of PF_6^- into a solution of Tf_2N^-⊂*trans*-**1** (*c* = 9.9 mM). The peak at -78.5 ppm shows gradual release of Tf_2N^- with increasing amount of PF_6^-. b) Zoom of part of the ^{19}F NMR spectrum shows fast exchange of PF_6^- on the ^{19}F NMR time scale.

S7.2 Encapsulation of Guest TfO⁻

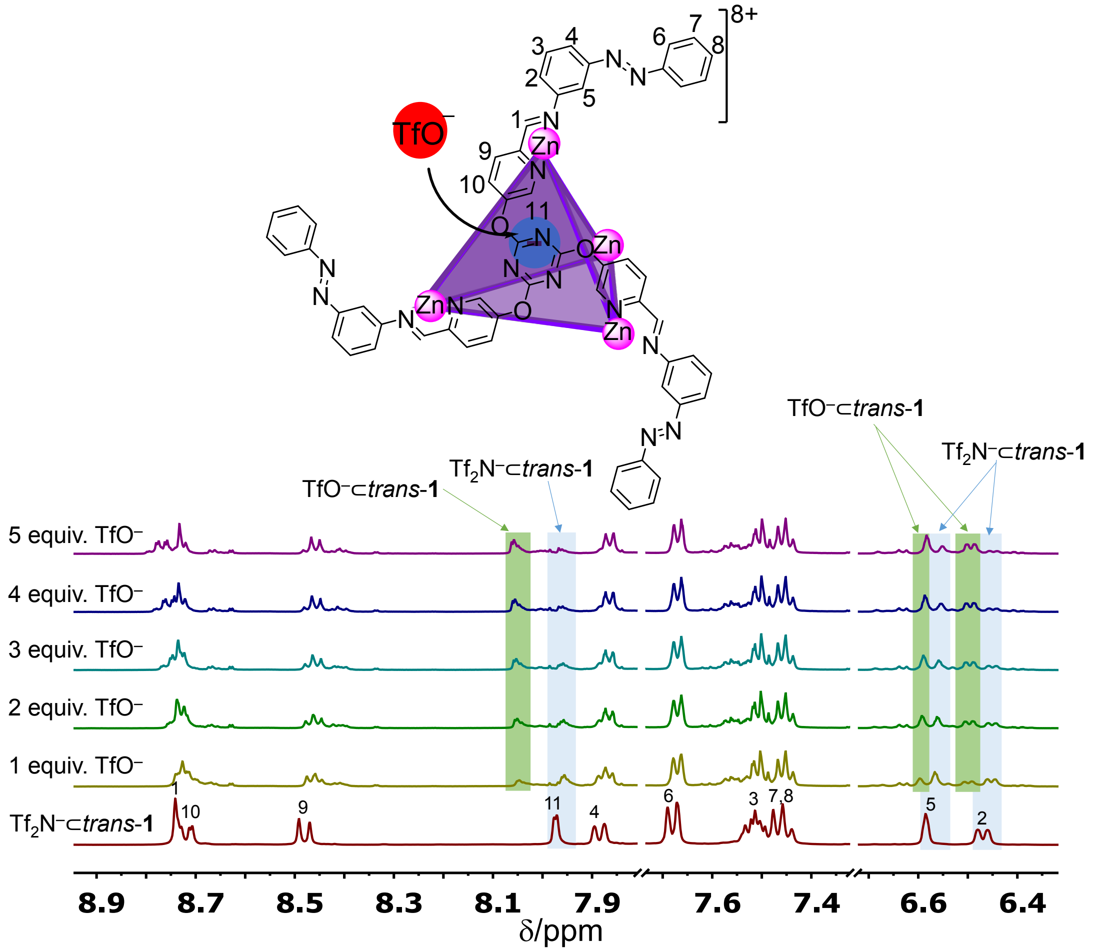

Figure S39. ^{1}H NMR (CD_3CN, 500 MHz, 25 °C) titration of TfO⁻into a solution of Tf_2N^- ⊂*trans*-**1** (9.9 mM).

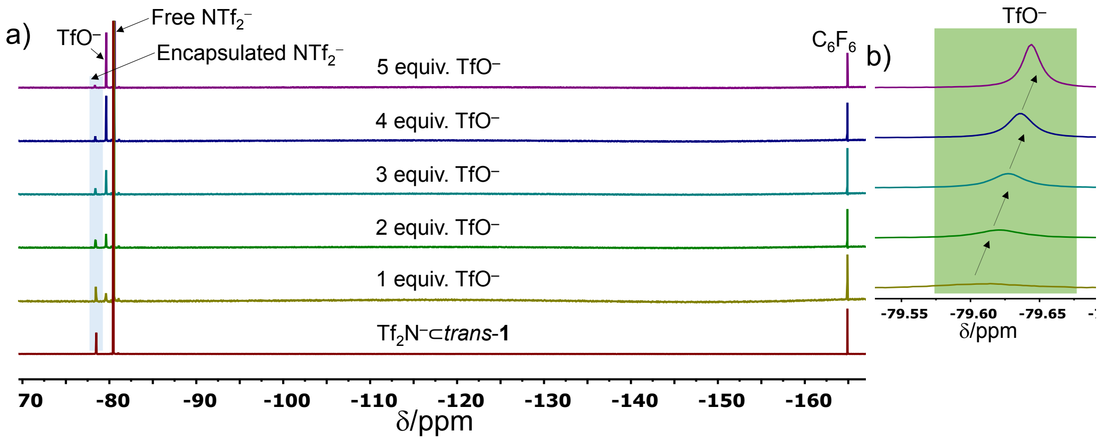

Figure S40. a) ^{19}F NMR (CD_3CN, 376 MHz, 25 °C) titrations of TfO⁻ into a solution of Tf_2N^-⊂*trans*-**1** (9.9 mM). The peak at -78.5 ppm shows gradual release of Tf_2N^- with increasing amount of TfO⁻. b) Zoom of part of the ^{19}F NMR spectrum shows fast exchange of TfO⁻ on the ^{19}F NMR time scale.

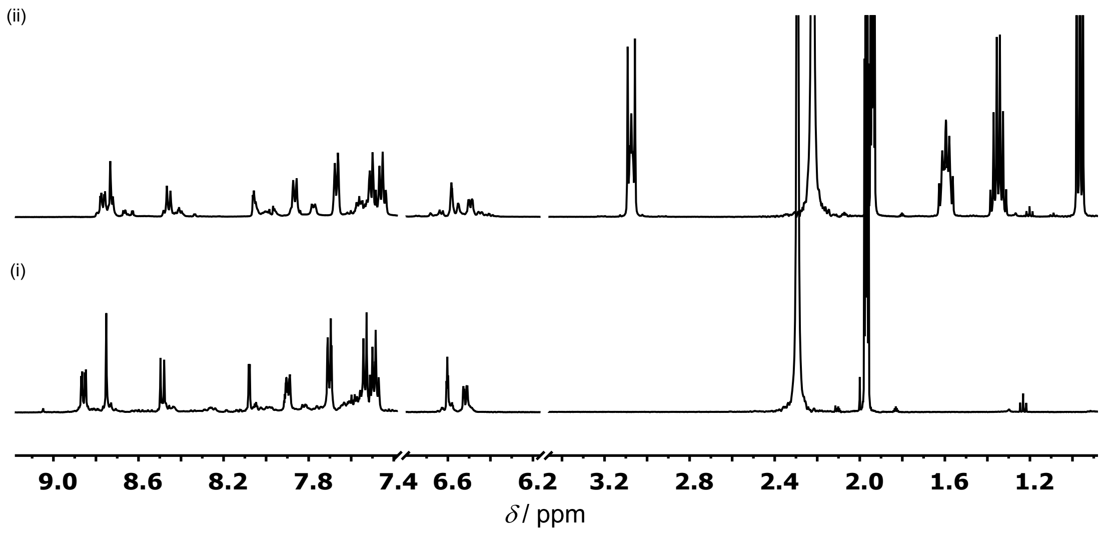

Figure S41. The comparison of ^{1}H NMR (500 MHz, CD_3CN, 25 °C) spectra of (i) *trans*-**1**·$[OTf]_8$ and (ii) $TfO^{-}\subset$*trans*-**1**.

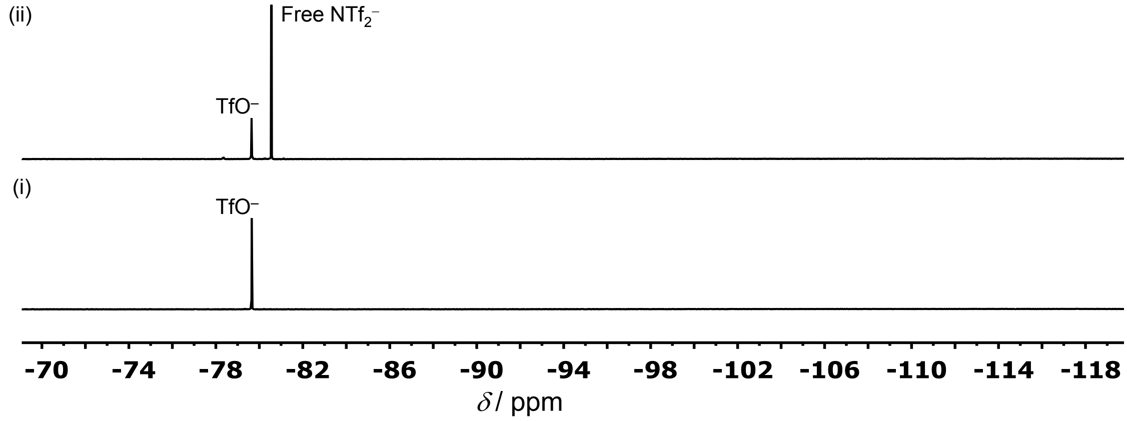

Figure S42. The comparison of ^{19}F NMR (376 MHz, CD_3CN, 25 °C) spectra of (i) *trans*-**1**·$[OTf]_8$ and (ii) $TfO^{-}\subset$*trans*-**1**.

S7.3 Encapsulation of Guest BF_4^-

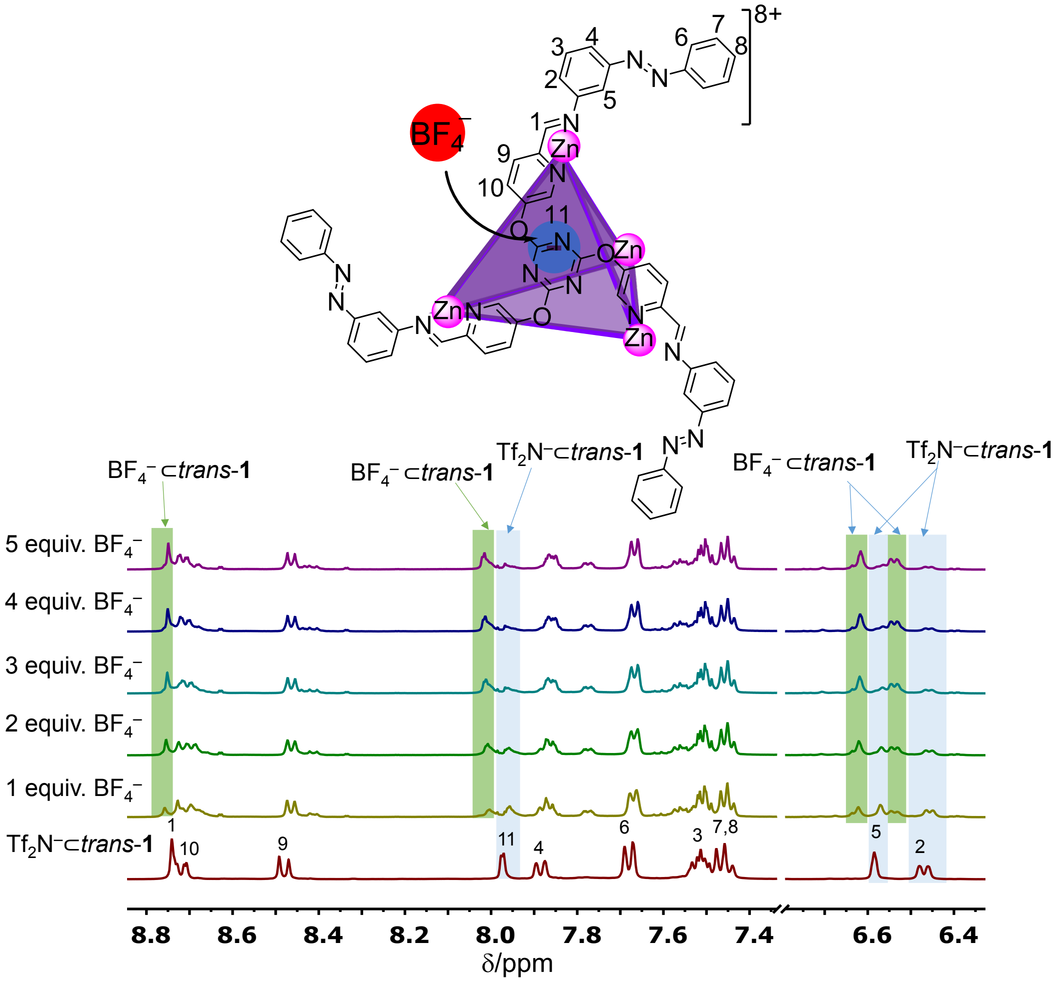

Figure S43. ^{1}H NMR (CD_3CN, 500 MHz, 25 °C) titration of BF_4^- into a solution of Tf_2N^- ⊂*trans*-**1** (9.9 mM).

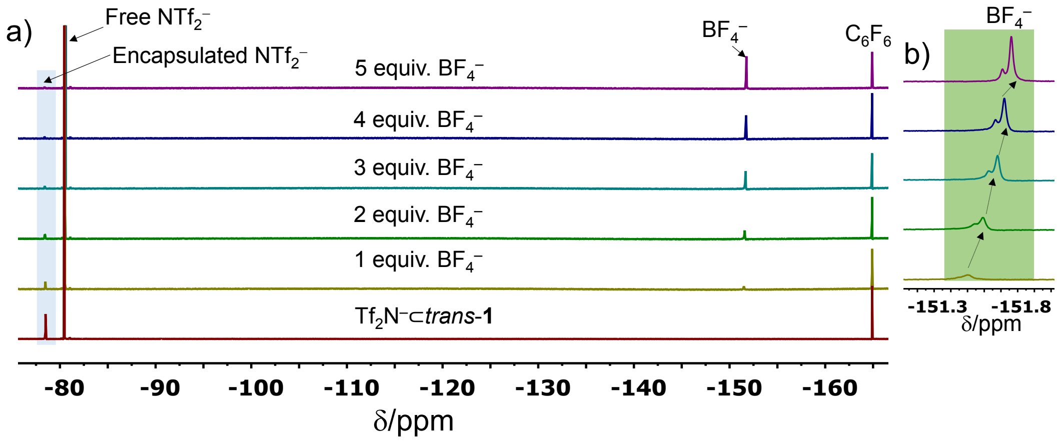

Figure S44. a) ^{19}F NMR (CD_3CN, 376 MHz, 25 °C) titration of BF_4^- into a solution of Tf_2N^-⊂*trans*-**1** (9.9 mM). The peak at -78.5 ppm shows gradual release of anion Tf_2N^- with increasing amount of BF_4^-. b) Zoom of part of the ^{19}F NMR shows fast exchange of BF_4^- on the ^{19}F NMR time scale.

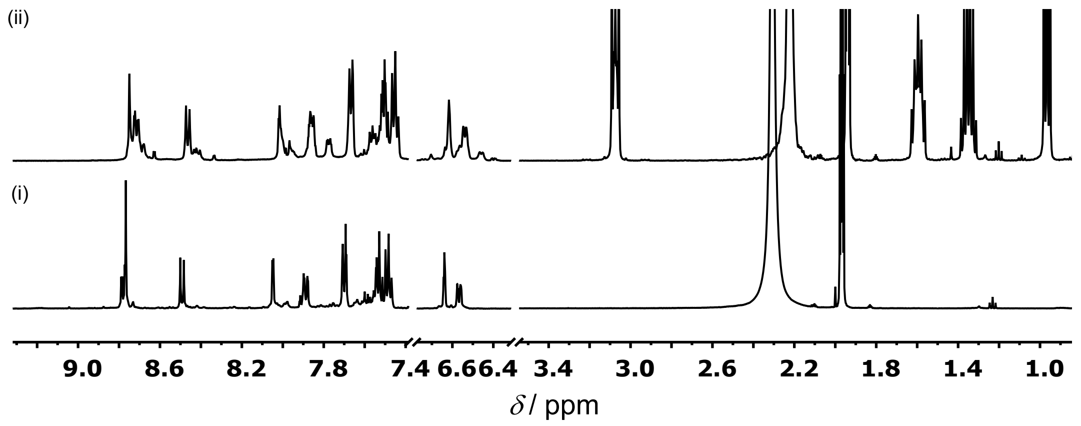

Figure S45. The comparison of ^{1}H NMR (500 MHz, CD_3CN, 25 °C) spectra of (i) *trans*-**1**·$[BF_4]_8$ and (ii) BF_4^-⊂*trans*-**1**.

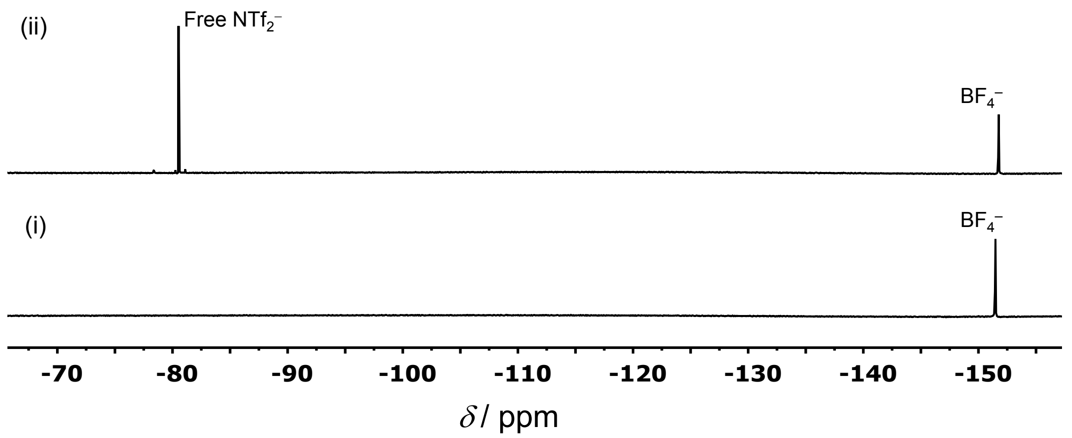

Figure S46. The comparison of ^{19}F NMR (376 MHz, CD_3CN, 25 °C) spectra of (i) *trans*-**1**·[$BF_4]_8$ and (ii) BF_4^-⊂*trans*-**1.**

S7.4 Comparison of ^{1}H and ^{19}F NMR spectra of All of the Host-Guest complexes

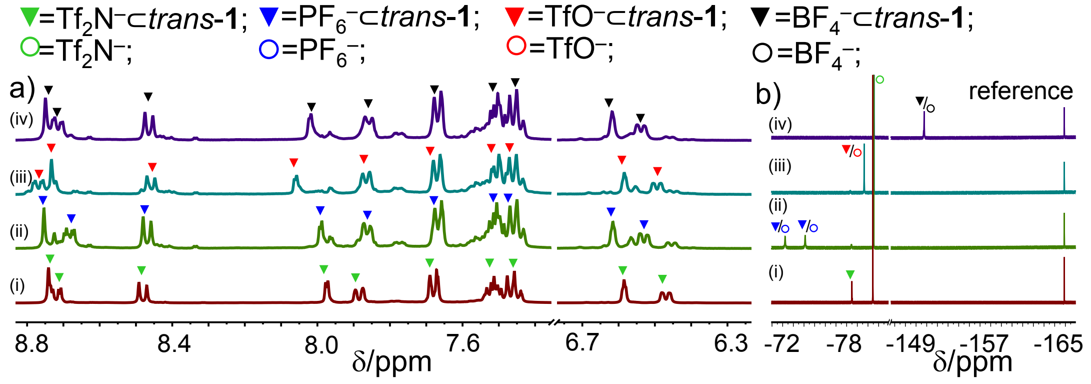

Figure S47. a) ^{1}H NMR spectra (CD_3CN, 400 MHz, 25 °C) and b) ^{19}F NMR spectra (CD_3CN, 376 MHz, 25 °C) of i) Tf_2N^{-}⊂*trans*-**1**, ii) PF_6^{-}⊂*trans*-**1**, iii) TfO^{-}⊂*trans*-**1**, and iv) BF_4^{-}⊂*trans*-**1**, respectively. PF_6^{-}⊂*trans*-**1**, TfO^{-}⊂*trans*-**1** and BF_4^{-}⊂*trans*-**1** are obtained by adding five equivalents of $TBAPF_6$, TBAOTf, or $TBABF_4$, respectively, to a solution of Tf_2N^{-}⊂*trans*-**1**. Green, blue, red and black solid triangles indicate the peaks of the *trans*-**1** bound to Tf_2N^{-}, PF_6^{-}, TfO^{-} and BF_4^{-}, respectively. The peaks of the free guests in (b) are marked with empty circles.

S7.5 Competitive Binding Studies[4]

A 9.9 mM solution of Tf_2N^{-}⊂*trans*-**1** was prepared and used directly for probing the relative binding strength of anions by ^{1}H NMR. Differing amounts of competing anions were added to the solution in order to obtain an accurate relative binding affinity. After each addition of the guest anion to the Tf_2N^{-} bound cage, the mixture was kept at 298 K for at least 2 days prior to recording the spectra to ensure the system had reached equilibrium.

In solution, there is the following equilibrium:

$$[HG\mathbf{1}] + [G\mathbf{2}] \rightleftarrows [HG\mathbf{2}] + [G\mathbf{1}]$$

The relative binding constant (K_{rel}) is calculated by the following equation:

$$K_{rel} = K_a{}^{2}/K_a{}^{1} = [HG\mathbf{2}]/[HG\mathbf{1}] \times [G\mathbf{1}]/[G\mathbf{2}]$$

Here K_{rel} is the relative binding constant; $K_a{}^{2}$ is the binding constant for guest 2 and $K_a{}^{1}$ is the binding constant for guest 1. $[G_1]_{total}$ is the total concentration of $\mathbf{G_1}$ prior to the addition of a competitive anion $\mathbf{G_2}$. After the addition of $\mathbf{G_2}$, $[HG_1]$ and $[HG_2]$ could be determined by integration of the corresponding ^{1}H NMR cage peaks. Then $[G_1]$, the

concentration of the free **G₁**, is equal to the difference between $[G_1]_{total}$ and $[HG_1]$. The concentration of the added **G₂**, $[G_2]_{add}$, could also be determined by integration of the TBA$^+$ resonances, and then $[G_2]$, the concentration of free **G₂**, is equal to the difference between $[G_2]_{add}$ and $[HG_2]$. Hence based on the above four concentrations, the relative binding constant K_{rel} was determined by ^{1}H NMR spectroscopy.

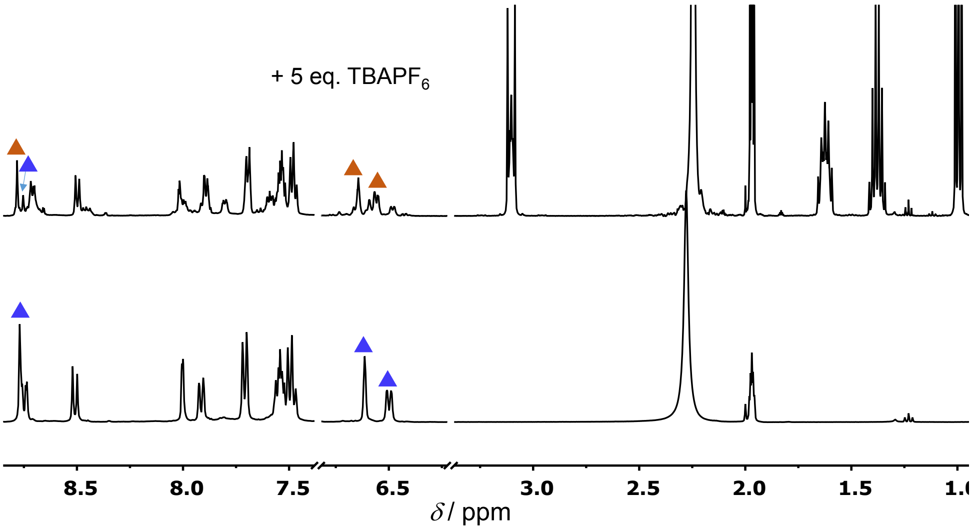

Figure S48. Addition of PF_6^- to a solution of Tf_2N^-⊂*trans*-**1** in CD_3CN monitored by ^{1}H NMR spectroscopy (500 MHz, 25 °C). The solution was left to equilibrate for 2 days following addition of $TBAPF_6$ prior to acquisition of the new spectrum. These results show that $K_{Tf2N-} = 6.8K_{PF6-}$.

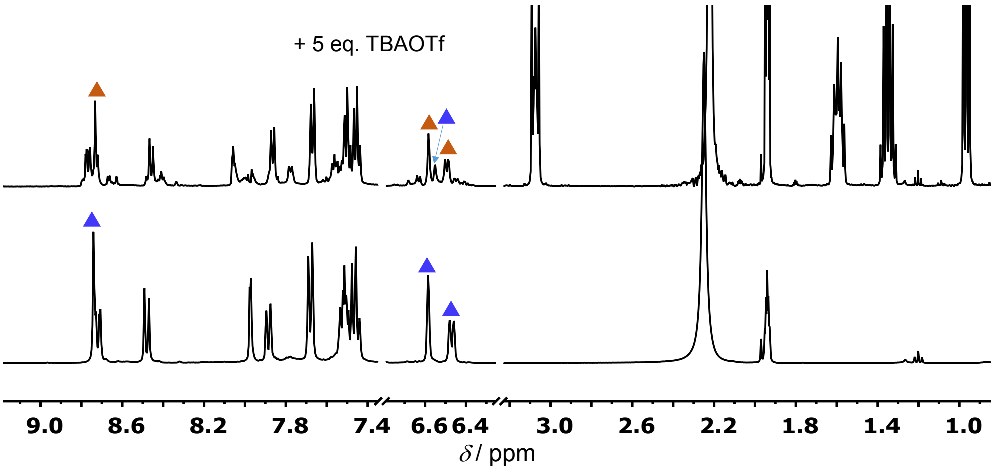

Figure S49. Addition of TfO⁻ to a solution of Tf_2N^-⊂trans-**1** in CD_3CN monitored by ^{1}H NMR spectroscopy (500 MHz, 25 °C). The solution was left to equilibrate for 2 days following addition of TBAOTf prior to acquisition of the new spectrum. The results show $K_{Tf2N-} = 9.6K_{TfO-}$.

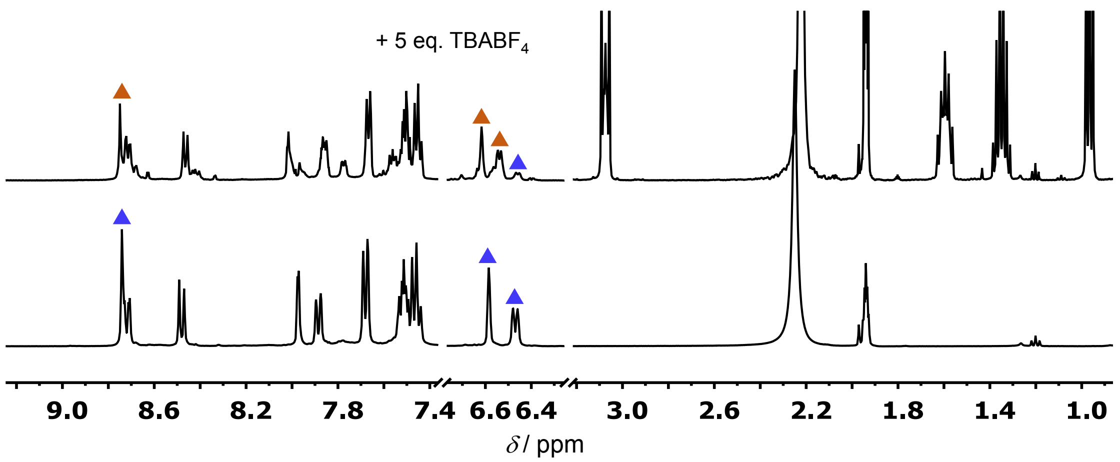

Figure S50. Addition of BF_4^- to a solution of $Tf_2N^- \subset$*trans*-**1** in CD_3CN monitored by ^1H NMR spectroscopy (500 MHz, 25 °C). The solution was left to equilibrate for 2 days following addition of $TBABF_4$ prior to acquisition of the new spectrum. The results show $K_{Tf2N-} = 21.3K_{BF4-}$.

From Figure S48-Figure S50, a hierarchy of binding affinities, Tf_2N^-> PF_6^- > TfO^- > BF_4^-, was obtained. These results support the inference that larger anions bind more strongly within **1**.

Anion	Volume ($Å^3$)	K_{rel} ($K_a{}^2/K_{Tf2N-}$)
Tf_2N^-	131	-
PF_6^-	90	0.15
TfO^-	86	0.11
BF_4^-	50	0.04

Table S2. Reported volumes[2,3] and relative binding affinities of the anions.

S7.6 Variable Temperature Studies

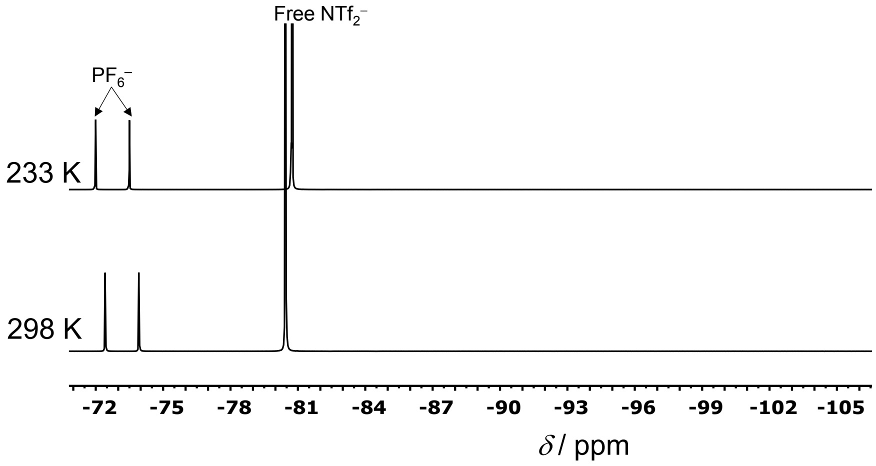

Figure S51. ^{19}F VT-NMR (CD_3CN, 376 MHz) of PF_6^-⊂*trans*-**1**. With lowering temperature the signals broadened and shifted, suggesting fast exchange of PF_6^- within cage *trans*-**1** on the NMR time scale.

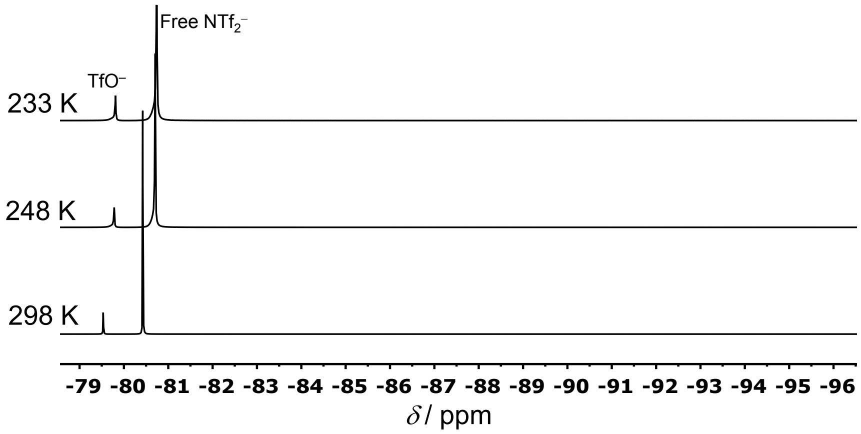

Figure S52. ^{19}F VT-NMR (CD_3CN, 376 MHz) of TfO^-⊂*trans*-**1**. With lowering temperature the signals broadened and shifted, suggesting fast exchange of TfO^- within cage *trans*-**1** on the NMR time scale.

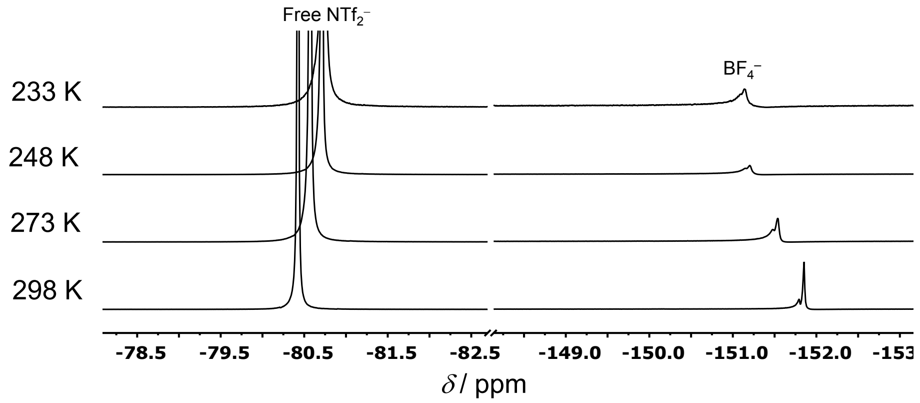

Figure S53. ^{19}F VT-NMR (CD_3CN, 376 MHz) of $BF_4^-\subset$*trans*-**1**. With lowering temperature the signals broadened and shifted, suggesting fast exchange of BF_4^- within cage *trans*-**1** on the NMR time scale.

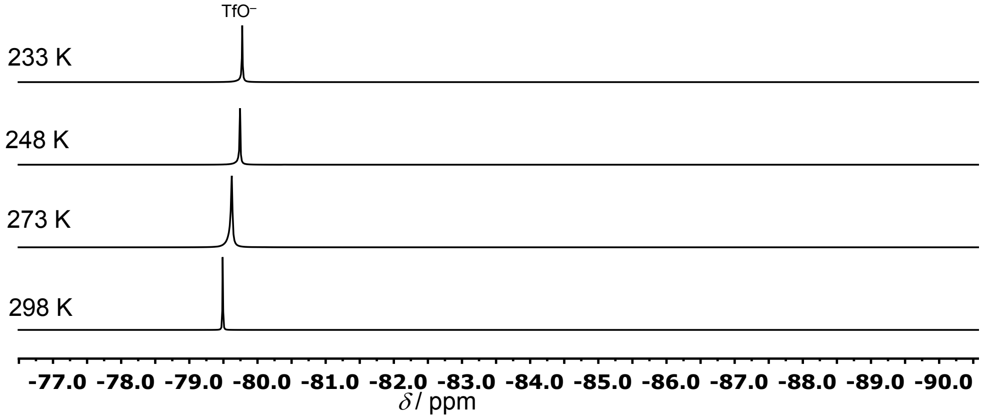

Figure S54. ^{19}F VT-NMR (CD_3CN, 376 MHz) of *trans*-**1**·$[OTf]_8$. With lowering temperature the signals broadened and shifted, suggesting fast exchange of TfO^- within cage *trans*-**1** on the NMR time scale.

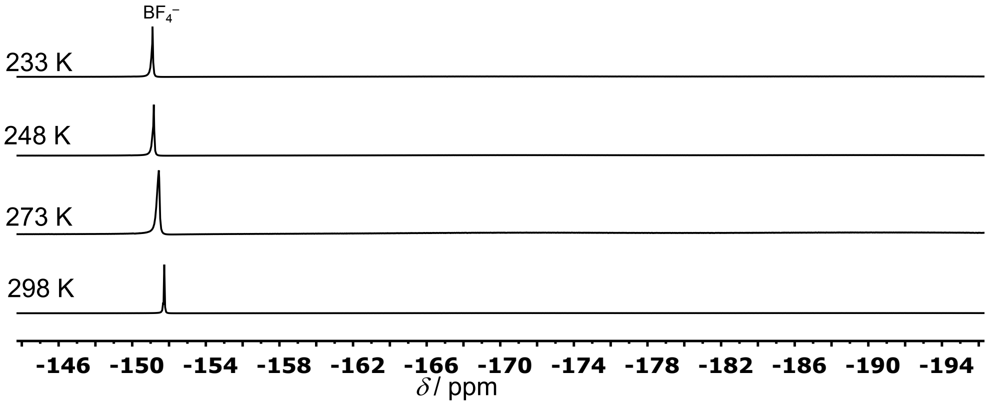

Figure S55. ^{19}F VT-NMR (CD_3CN, 376 MHz) of *trans*-**1**·$[BF_4]_8$. With lowering temperature the signals broadened and shifted, suggesting fast exchange of BF_4^- within cage *trans*-**1** on the NMR time scale.

S7.7 Investigation with Neutral Guests

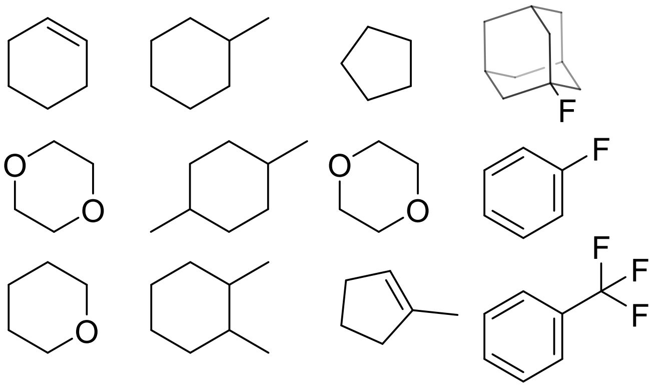

Figure S56. Neutral guests investigated.

A series of neutral molecules (Figure S56), cyclohexene, methylcyclohexane, cyclopentane, 1,4-dioxane, 1,4-dimethylcyclohexane, 1,3-dioxane, tetrahydropyran, 1,2-dimethylcyclohexane, 1-methylcyclopentene, trifluorotoluene, fluorobenzene, and 1-fluoroadamantane, were investigated as potential guests for *trans*-**1**. A solution of *trans*-**1** was prepared at a concentration of 3.3 mM in CD_3CN and all prospective guest molecules (10 equivalents of each guest relative to *trans*-**1**) were added directly to the NMR tube. ^{1}H NMR and ^{19}F NMR spectra were recorded 2 hours after the addition. No evidence of guest encapsulation was observed by NMR.

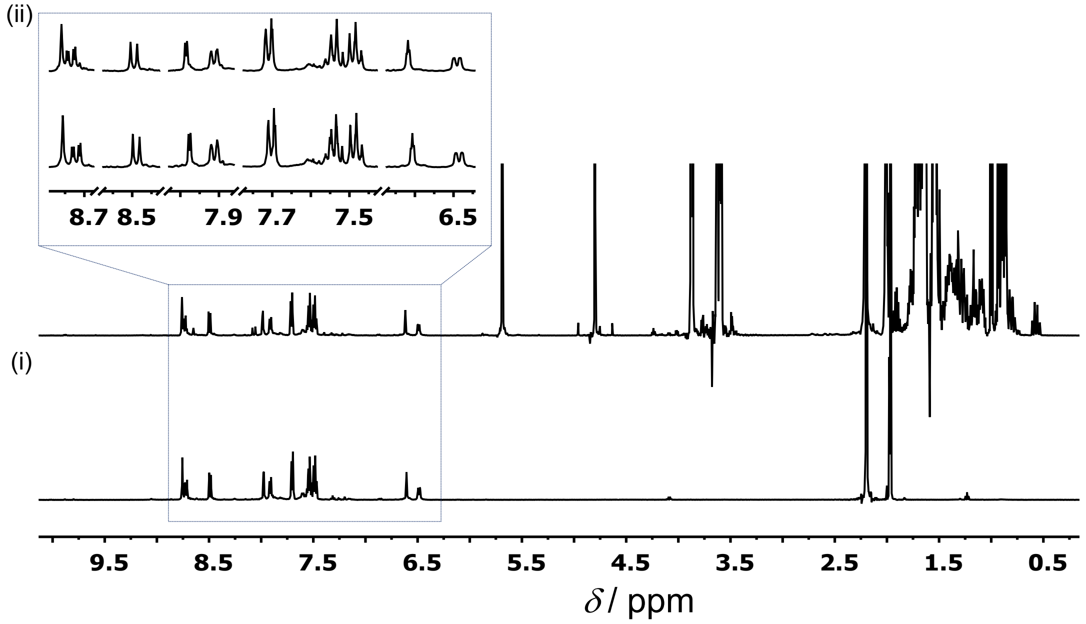

Figure S57. ^{1}H NMR (500 MHz, CD_3CN, 25 °C) of (i) *trans*-**1** and (ii) *trans*-**1** with a mixture of cyclohexene, methylcyclohexane, cyclopentane, 1,4-dioxane, 1,4-dimethylcyclohexane, 1,3-dioxane, tetrahydropyran, 1,2-dimethylcyclohexane and 1-methylcyclopentene in excess.

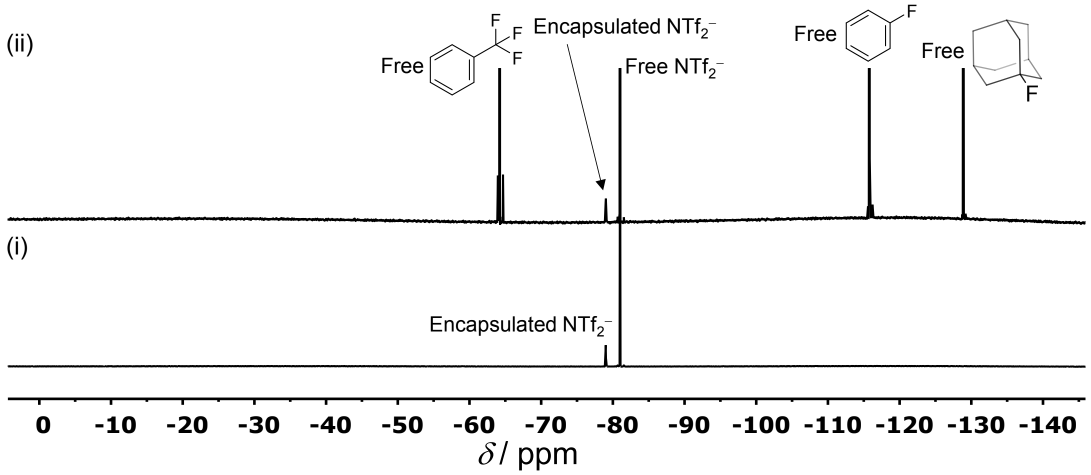

Figure S58. ^{19}F NMR (376 MHz, CD_3CN, 25 °C) of (i) *trans*-**1** and (ii) *trans*-**1** with a mixture trifluorotoluene, fluorobenzene, and 1-fluoroadamantane in excess. No displacement of Tf_2N^- was observed by any of these prospective guests.

S8 Investigation of Photoswitching Properties of *Trans*-1 Encapsulating Different Guests

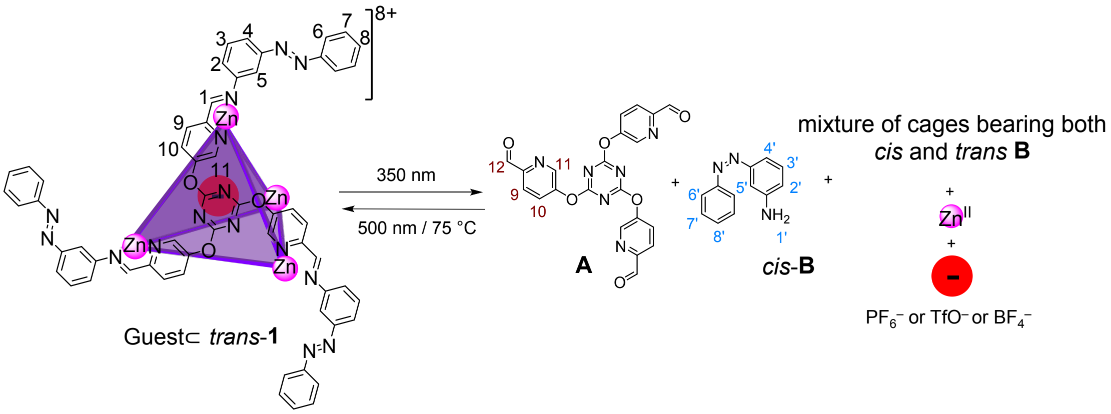

Figure S59. Disassembly and assembly of cage Guest⊂*trans*-**1** and subsequent guest (PF_6^-/TfO⁻/ BF_4^-) release and uptake.

In an NMR tube, **A** (2.20 mg, 4.95 µmol, 4.0 equiv) in 500 µL of CD_3CN was combined with **B** (2.92 mg, 14.8 µmol, 12.0 equiv) and $Zn(NTf_2)_2$ (3.10 mg, 4.95 µmol, 4.0 equiv). To this mixture, five equivalents of $TBAPF_6$ (or TBAOTf or $TBABF_4$) were added and the NMR tube was irradiated at 350 nm. After 10 minutes the ^{1}H NMR spectrum was recorded. For the reverse process, the same solution was irradiated at 500 nm for 30 min or heated to 75 °C for 10 h and subsequently the ^{1}H NMR spectrum was recorded.

S8.1 Reversible photoswitching of cage $PF_6^- \subset$trans-1

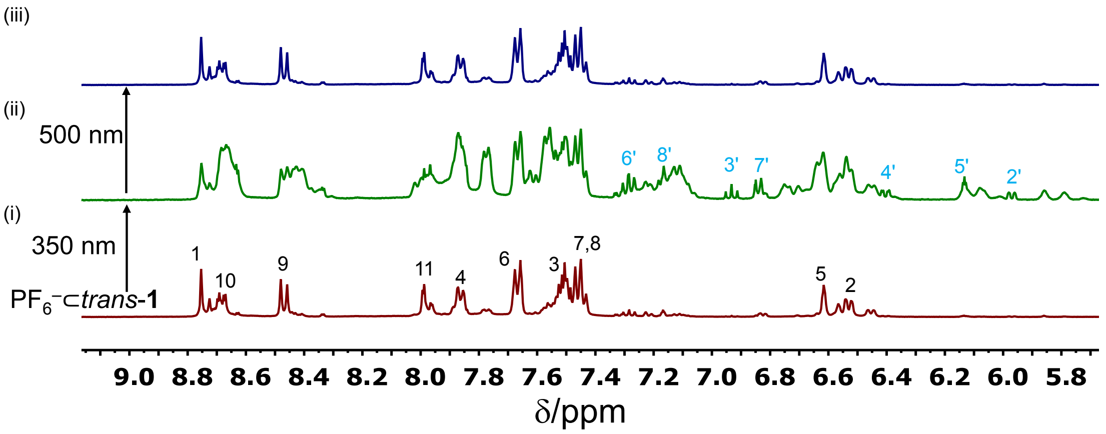

Figure S60. ^{1}H NMR spectra (500 MHz, CD_3CN, 25 °C) of (i) $PF_6^- \subset$*trans*-**1**, (ii) the same sample after irradiation at 350 nm for 10 min (labeled peaks correspond to free subcomponent *cis*-**B**) and (iii) the same sample after irradiation at 500 nm for 30 min.

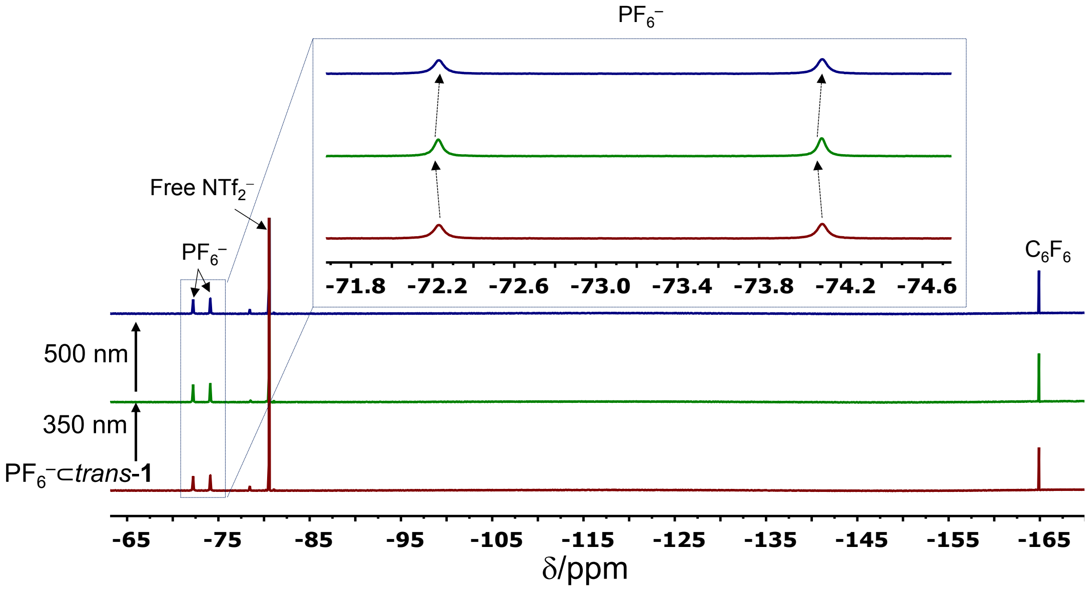

Figure S61. ^{19}F NMR spectra (376 MHz, CD_3CN, 25 °C) of $PF_6^- \subset$*trans*-**1** during photoisomerisation, as per Figure S32.

S8.2 Reversible photoswitching of cage TfO⁻⊂*trans*-1

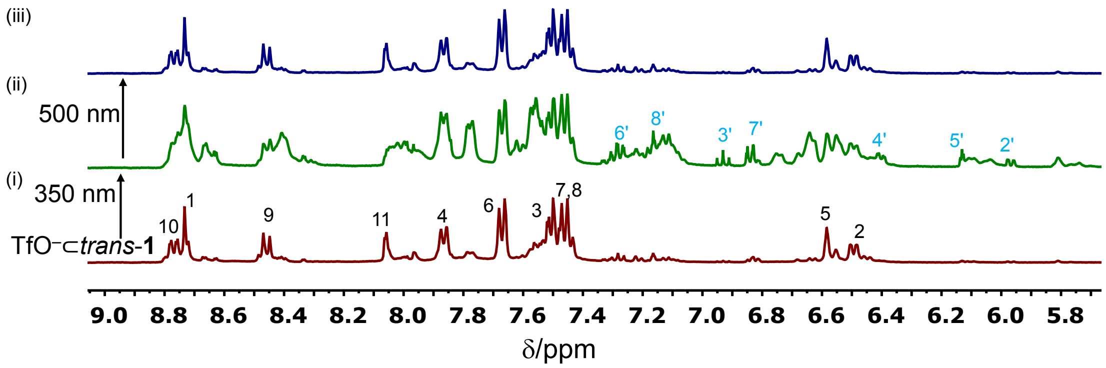

Figure S62. ^{1}H NMR spectra (500 MHz, CD_3CN, 25 °C) of (i) TfO⁻⊂*trans*-**1**, (ii) after irradiation at 350 nm for 10 min (labeled peaks correspond to free subcomponent *cis*-**B**), and (iii) after irradiation at 500 nm for 30 min.

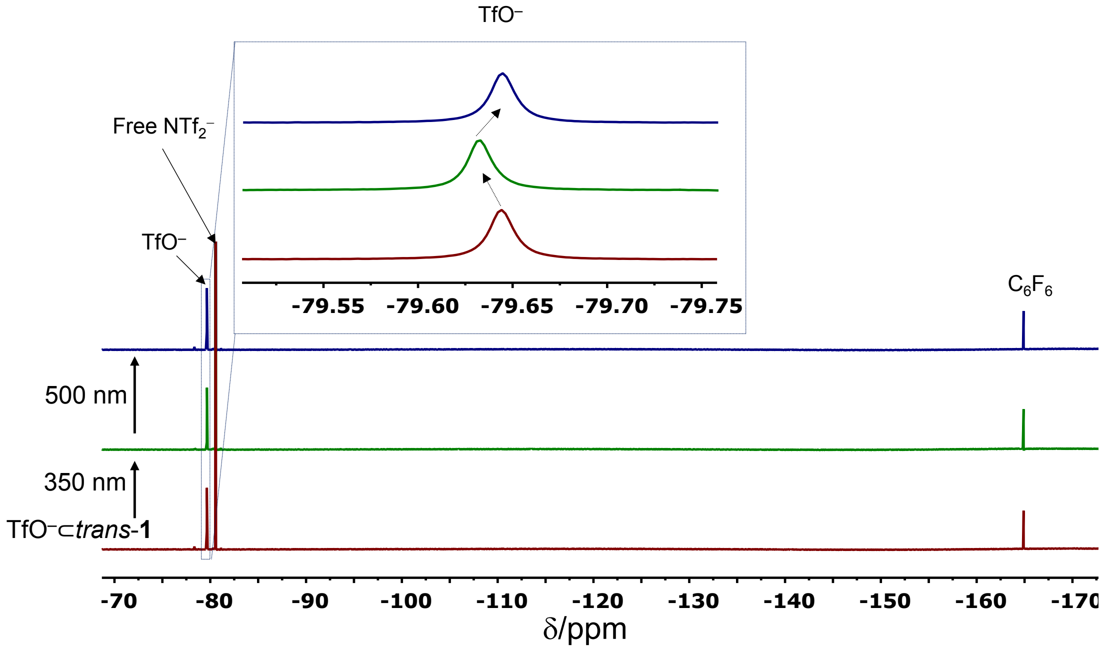

Figure S63. ^{19}F NMR spectra (376 MHz, CD_3CN, 25 °C) of photoswitching of TfO⁻⊂*trans*-**1**, as per Figure S34.

S8.3 Reversible photoswitching of cage BF_4^-⊂***trans*-1**

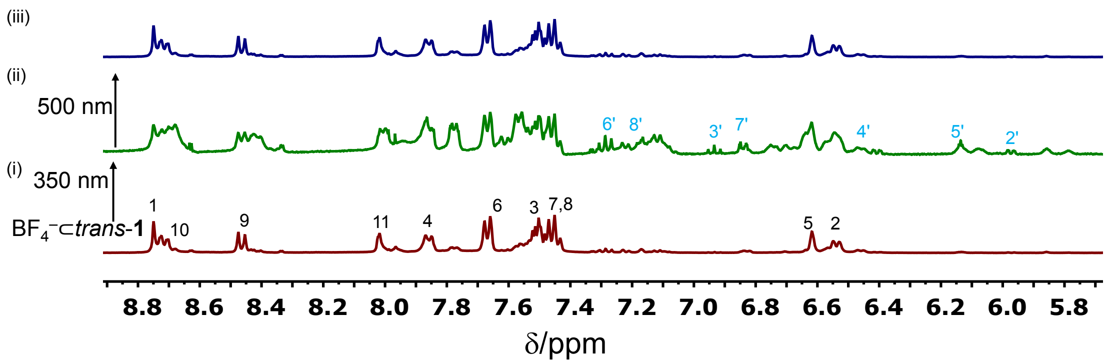

Figure S64. ^{1}H NMR spectra (500 MHz, CD_3CN, 25 °C) of (i) BF_4^-⊂*trans*-**1**, (ii) after UV irradiation at 350 nm for 10 min (labeled peaks correspond to free subcomponent *cis*-**B**), and (iii) after irradiation at 500 nm for 30 min.

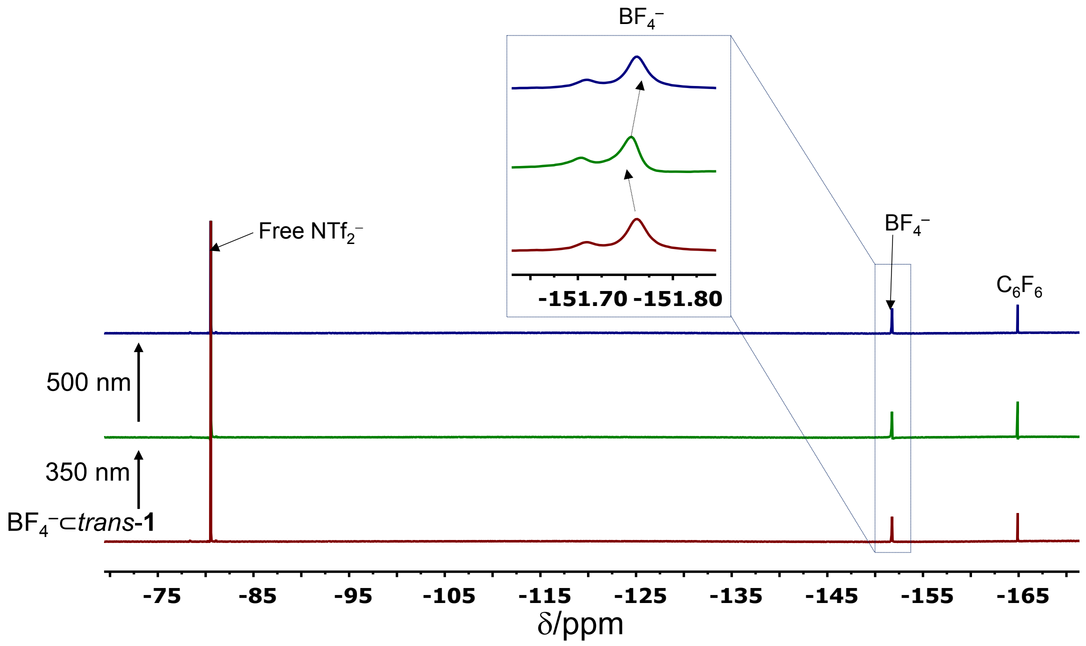

Figure S65. ^{19}F NMR spectra (376 MHz, CD_3CN, 25 °C) of photoswitching of BF_4^-⊂*trans*-**1**.

S8.4 Reversible Photoswitching of Cage *Trans*-1·[OTf]$_8$

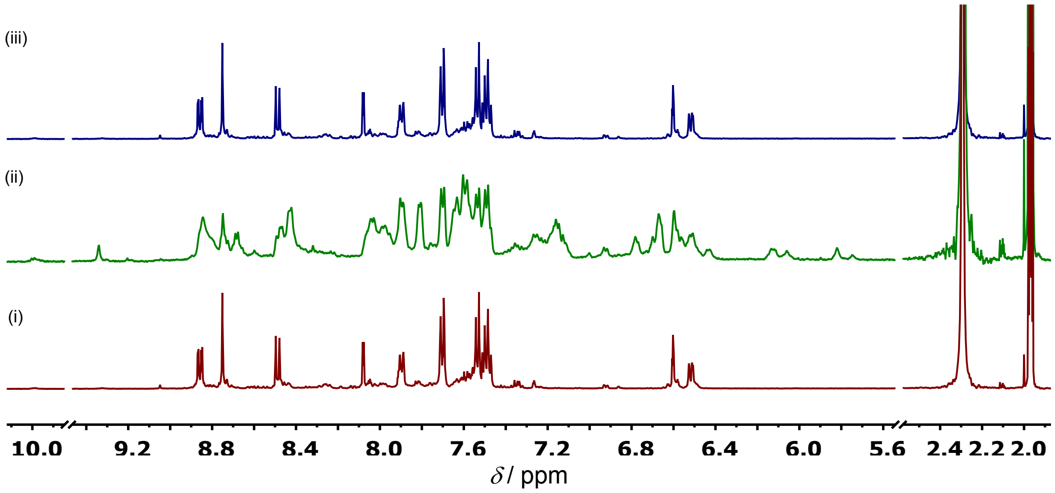

Figure S66. Reversible photoswitching of cage *trans*-**1**·[OTf]$_8$. ^{1}H NMR spectra (500 MHz, CD_3CN, 25 °C) of (i) cage *trans*-**1**·[OTf]$_8$; (ii) after 350 nm irradiation for 10 min and (iii) after irradiation at 500 nm for 30 min.

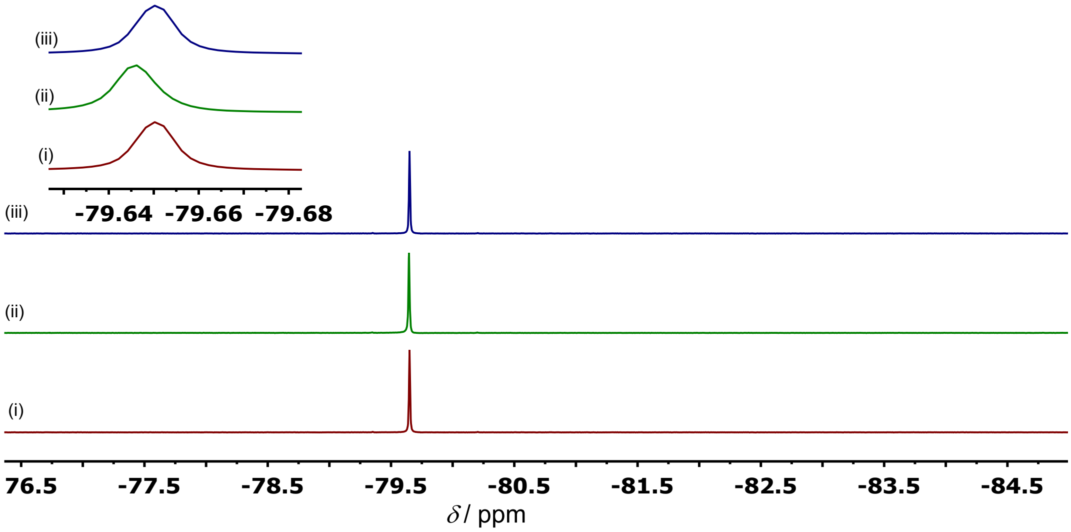

Figure S67. Reversible photoswitching of cage *trans*-**1**·[OTf]$_8$. ^{19}F NMR spectra (376 MHz, CD_3CN, 25 °C) of (i) cage *trans*-**1**·[OTf]$_8$; (ii) after 350 nm irradiation for 10 min and (iii) after irradiation at 500 nm for 30 min.

S8.5 Reversible Photoswitching of Cage *Trans*-1·$[BF_4]_8$

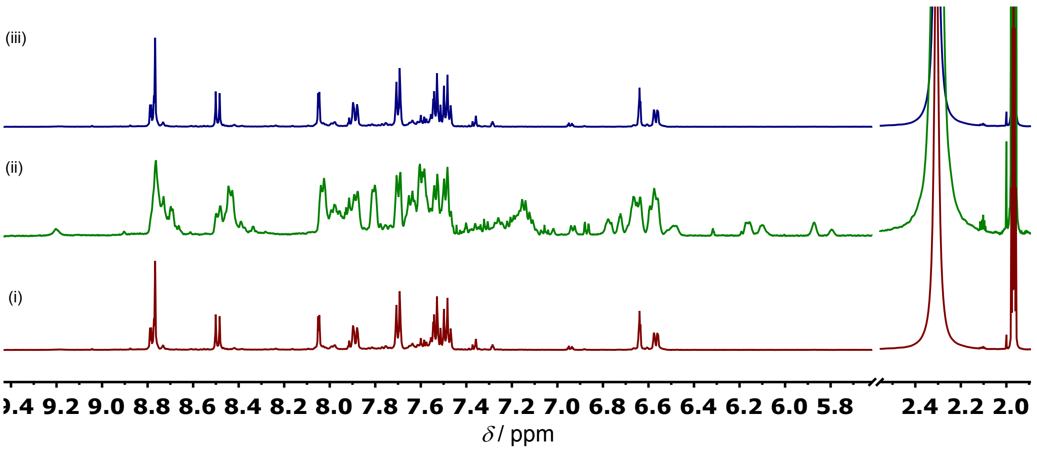

Figure S68. Reversible photoswitching of cage *trans*-**1**·$[BF_4]_8$. ^1H NMR spectra (500 MHz, CD_3CN, 25 °C) of (i) cage *trans*-**1**·$[BF_4]_8$; (ii) after 350 nm irradiation for 10 min and (iii) after irradiation at 500 nm for 30 min.

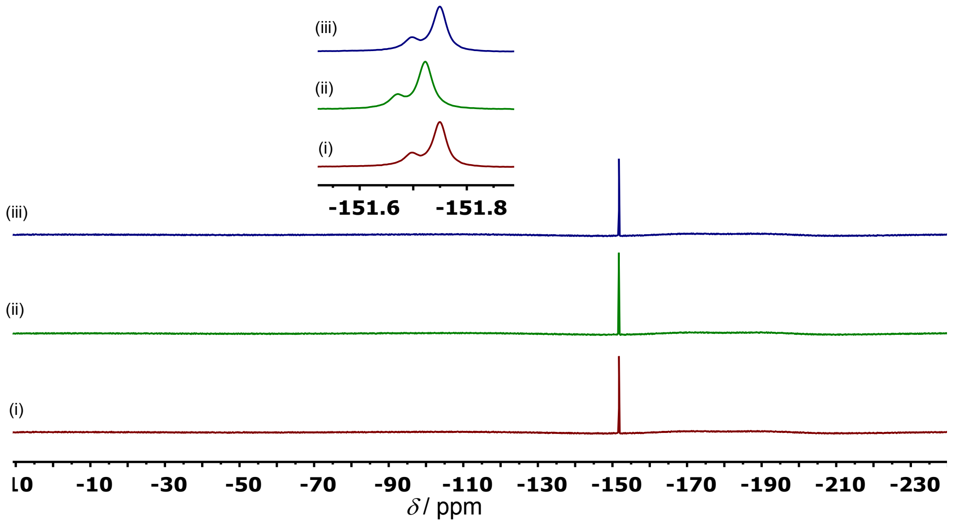

Figure S69. Reversible photoswitching of cage *trans*-**1**·$[BF_4]_8$. ^{19}F NMR spectra (376 MHz, CD_3CN, 25 °C) of (i) cage *trans*-**1**·$[BF_4]_8$; (ii) after 350 nm irradiation for 10 min and (iii) after irradiation at 500 nm for 30 min.

S9 X-ray crystallography

Crystals of cage **2** [$[Fe_4L_4]\cdot 8NTf_2$] were grown by diffusion of diethyl ether into a acetonitrile solution of the complex. Data were collected at Beamline I19 of Diamond Light Source employing silicon double crystal monochromated synchrotron radiation (0.6889 Å) with ω and *ψ* scans at 100(2) K.[5] Data integration and reduction were undertaken with Rigaku CrystalClear.[6] Subsequent computations were carried out using the WinGX-32 graphical user interface.[7] A multi-scan empirical absorption correction was applied to the data using Rigaku CrystalClear.[6] The structure was solved by charge flipping using Superflip[8] then refined and extended with SHELXL.[9] Carbon-bound hydrogen atoms were included in idealised positions and refined using a riding model. Disorder was modelled using standard crystallographic methods including constraints and restraints where necessary.

The crystals employed immediately lost solvent after removal from the mother liquor and rapid handling prior to flash cooling in the cryostream was required to collect data. Despite these measures and the use of synchrotron radiation few reflections at greater than 1.15 Å resolution were observed and the data were trimmed accordingly. Nevertheless, the quality of the data is far more than sufficient to establish the connectivity of the structure. The asymmetric unit was found to contain one complete Fe_4L_4 assembly as well as associated counterions and solvent molecules.

Due to the limited resolution of the data, bond length restraints were applied to some areas of the structure showing higher levels of thermal motion and some aromatic rings were modelled as rigid groups (AFIX 66). Thermal parameter restraints (SIMU, RIGU) were applied to all atoms except for iron.

The anions within the structure show evidence of disorder. The encapsulated triflimide was modelled as disordered over two locations and the fluorine atoms of another were also modelled over two locations. Two triflimides were modelled with partial occupancy and their occupancies allowed to freely refine. Several others show evidence of further disorder which could not be resolved due to the limited resolution of the data. Bond length and angle restraints were applied to all anions to achieve a reasonable

geometry. One complete anion and some additional minor occupancy positions of the anions could not be located in the electron density map and were not included in the model resulting in a discrepancy of ca. 1.9 counterions per Fe_4L_4 assembly.
Consequently the SQUEEZE[10] function of PLATON[11] was employed to remove the contribution of the electron density associated with the remaining anions and further highly disordered solvent, which gave a potential solvent accessible void of 7761 Å^3 per unit cell (a total of approximately 2055 electrons). Diffuse solvent molecules could not be assigned to acetonitrile or diethyl ether and were therefore not included in the formula. Consequently, the molecular weight and density given above are underestimated.
CheckCIF gives one A and two B level alerts, all resulting from the limited resolution of the data.

Crystallographic data have been deposited with the CCDC (2194876).

Formula $C_{192}H_{148}F_{48}Fe_4N_{46}O_{45}S_{16}$, *M* 5467.91, Monoclinic, space group P 21/n (#14), *a* 25.840(6), *b* 30.214(6), *c* 37.200(8) Å, *β* 93.828(3), *V* 28979(11) Å^3, D_c 1.253 g cm^{-3}, *Z* 4, crystal size 0.030 by 0.010 by 0.010 mm, colour purple, habit prism, temperature 100(2) Kelvin, *λ*(synchrotron) 0.6889 Å, *μ*(synchrotron) 0.372 mm^{-1}, T(CRYSTALCLEAR)$_{min,max}$ 0.4858, 0.7448, $2\theta_{max}$ 34.86, *hkl* range -22 22, -26 26, -32 31, *N* 104228, N_{ind} 19951(R_{merge} 0.1244), N_{obs} 12517(I > 2σ(I)), N_{var} 2963, residuals* $R1(F)$ 0.1041, $wR2(F^2)$ 0.3197, GoF(all) 1.039, $\Delta\rho_{min,max}$ -0.631, 1.078 e$^-$ Å^{-3}.

*$R1 = \Sigma||F_o| - |F_c||/\Sigma|F_o|$ for $F_o > 2\sigma(F_o)$; $wR2 = (\Sigma w(F_o^2 - F_c^2)^2/\Sigma (wF_c^2)^2)^{1/2}$ all reflections
$w=1/[\sigma^2(F_o^2)+(0.1170P)^2+460.7421P]$ where $P=(F_o^2+2F_c^2)/3$

S10 Volume Calculations

In order to determine the available void spaces within the structure of cage **2**, Molovol[12] calculations based on the crystal structures obtained in this study were performed with anions, disorder and solvent molecules removed. A probe with a radius of 2.4 Å was employed in all cases. The standard parameters are tabulated below, and the results are shown in Figure S70.

Probe mode: one probe

Probe radius: 2.4 Å

Grid resolution: 0.1 Å

Optimization depth: 4

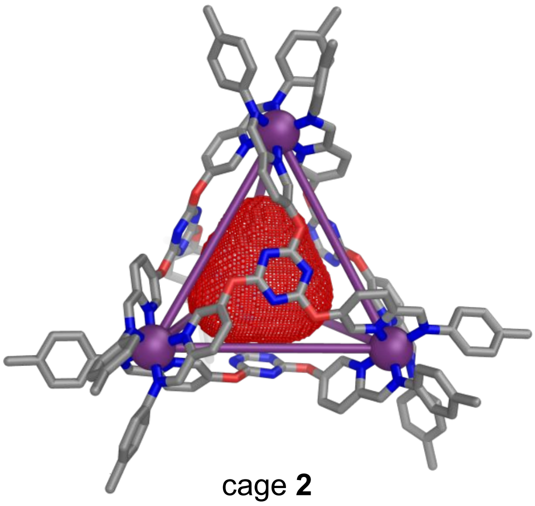

cage **2**

volume = 205 Å^3

Figure S70. MoloVol-calculated void space (red mesh) within the crystal structures of cage **2**.

S11 MM3 Models and Calculated Energies

Structures and energies were calculated using SCIGRESS,[13] running MM3 force field.

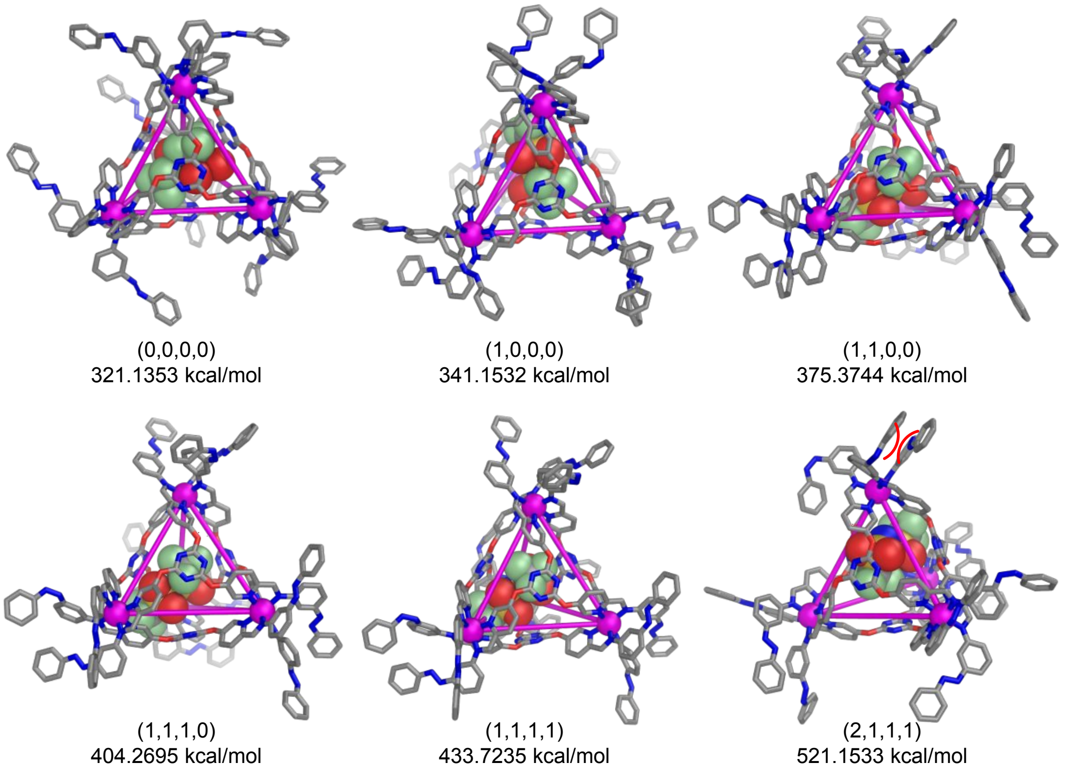

Figure S71. MM3-optimized molecular model of *trans*-**1**, and cages with 1, 2, 3, 4 and 5 *cis*-diazo moieties. The switching up to 4 *cis* isomers in each step increases the energy by about 25–35 kcal mol^{-1} but switching from 4 *cis* to 5 *cis* increases the energy by about 88 kcal mol^{-1}. These results thus support the inference that only 4 *cis* isomers can be tolerated by the cage before decomposition.

S12 Control Experiments

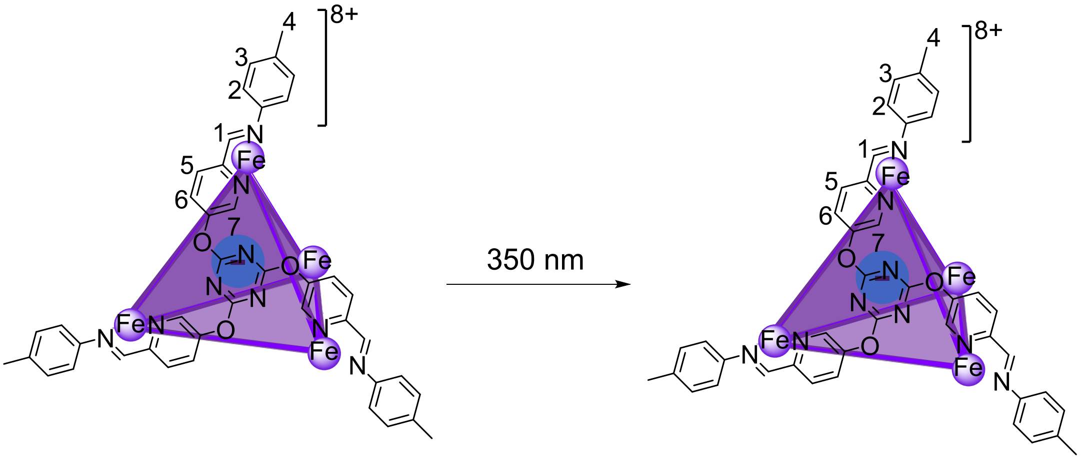

Figure S72. A control experiment with model cage **2** subjected to UV light at 350 nm for 10 min.

In an NMR tube, ligand **A** (4.0 mg, 9.0 µmol), *p*-toluidine, (2.9 mg, 27 µmol), and iron(II) triflimide [Fe(NTf$_2$)$_2$] (5.5 mg, 9.0 µmol) were dissolved in 500 µL of CD$_3$CN. An NMR spectrum was measured immediately after irradiation at 350 nm for 10 min.

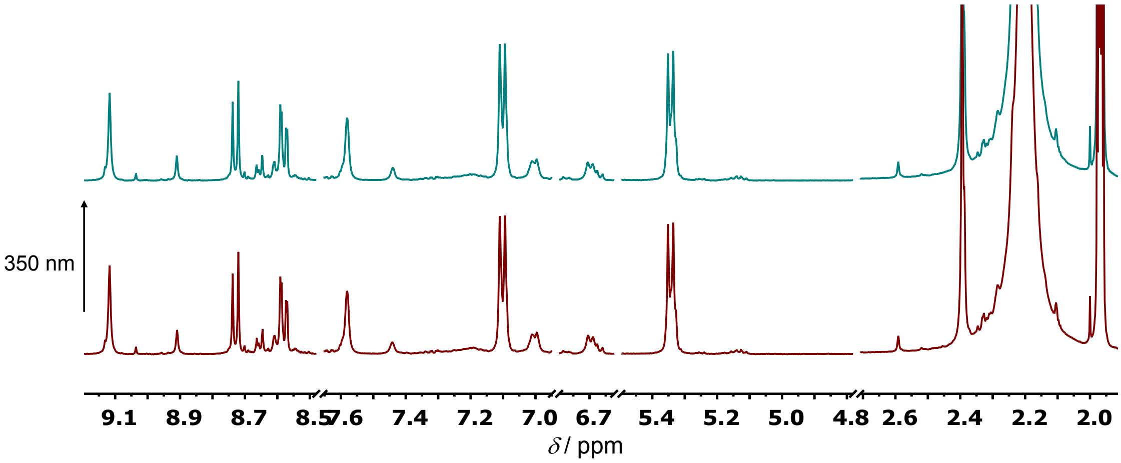

Figure S73. ^{1}H NMR (500 MHz, CD$_3$CN, 25 °C) of a model cage (cage **2**) before and after UV light irradiation for 10 min. No disassembly was observed, in contrast with cage *trans*-**1**.

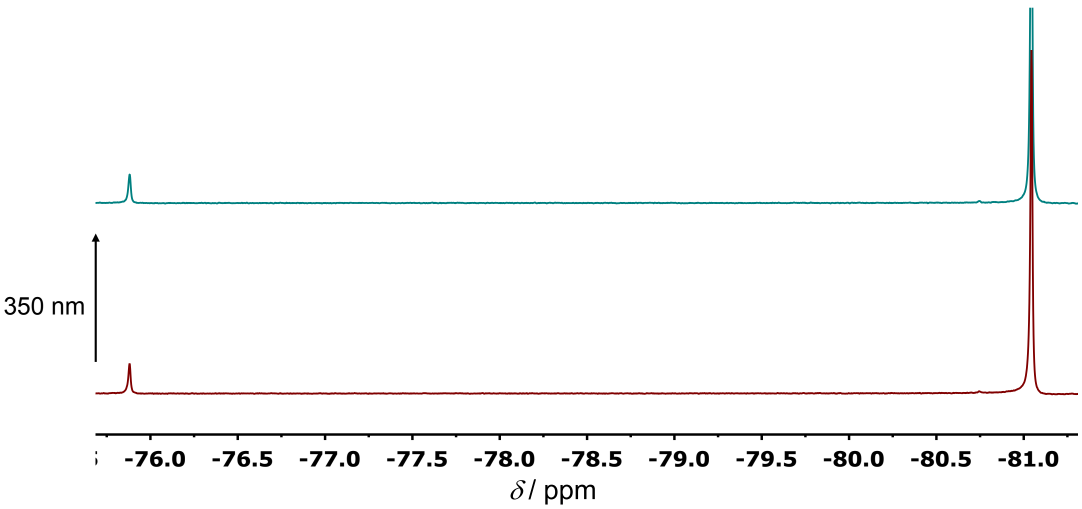

Figure S74. ^{19}F NMR spectra (376 MHz, CD_3CN, 25 °C) of a model cage (cage **2**) before and after UV light irradiation. No Tf_2N^- guest release was observed, in contrast with cage *trans*-**1**.

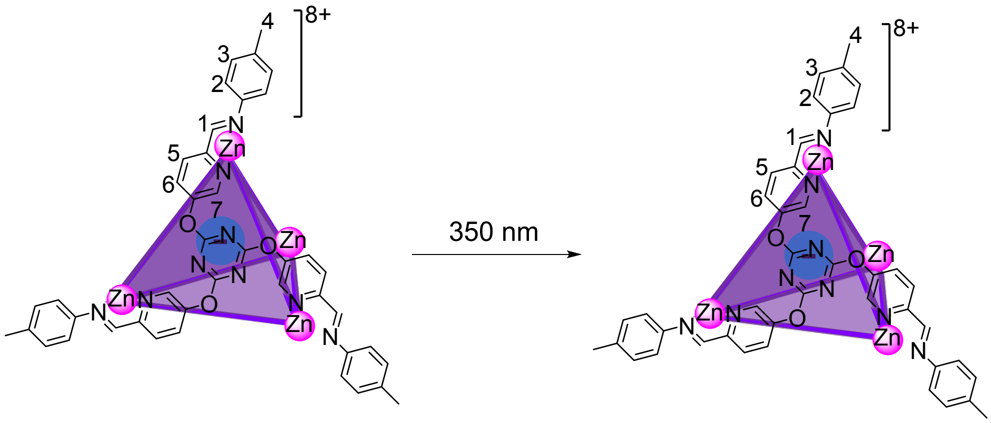

Figure S75. A control experiment with a model cage having the framework of cage **2** but with Zn^{II} instead of Fe^{II} subjected to UV light at 350 nm for 10 min.

In an NMR tube, ligand **A** (4.0 mg, 9.0 μmol), *p*-toluidine, (2.9 mg, 27 μmol), and iron(II) triflimide [$Zn(NTf_2)_2$] (5.6 mg, 9.0 μmol) were dissolved in 500 μL of CD_3CN. NMR spectra were measured immediately after irradiation at 350 nm for 10 min.

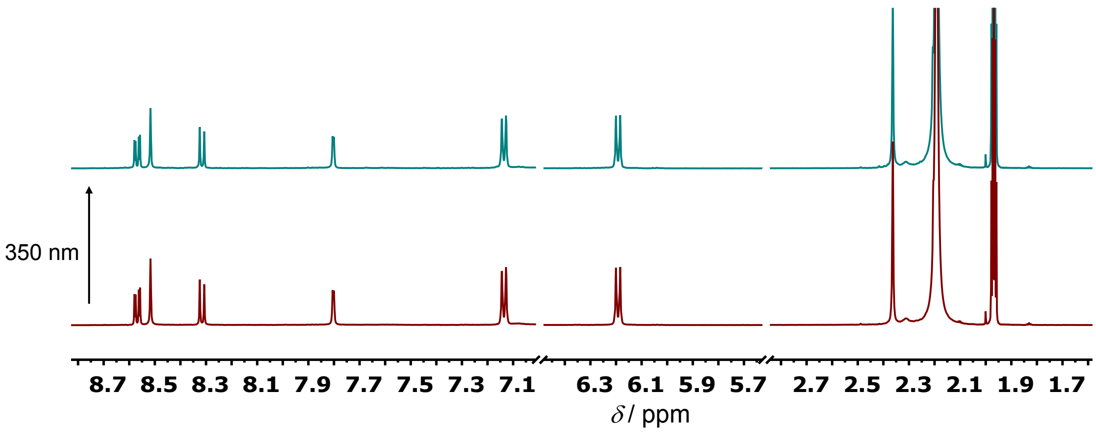

Figure S76. ^{1}H NMR (500 MHz, CD_3CN, 25 °C) of a model cage (cage **2** with Zn^{II} instead of Fe^{II}) before and after UV light irradiation. No disassembly was observed, in contrast with cage *trans*-**1**.

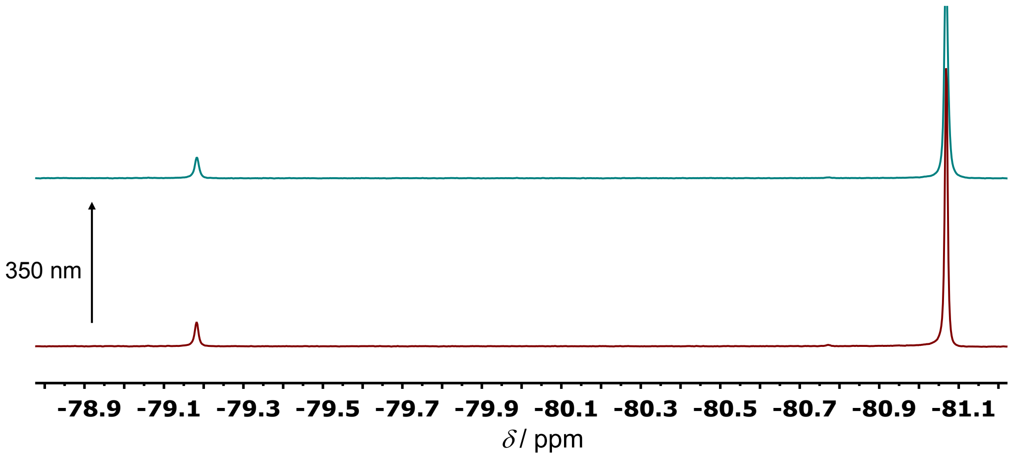

Figure S77. ^{19}F NMR spectra (376 MHz, CD_3CN, 25 °C) of a model cage (cage **2** with Zn(II) instead of Fe(II)) before and after UV light irradiation. No Tf_2N^- guest release was observed, in contrast with cage *trans*-**1**.

S13 References

1. Vapaavuori, J.; Goulet-Hanssens, A.; Heikkinen, I. T. S.; Barrett, C. J.; Priimagi, A. Are Two Azo Groups Better than One? Investigating the Photoresponse of Polymer-Bisazobenzene Complexes. *Chem. Mater.* **2014**, *26*, 5089−5096.

2. Gayton, J. N.; Autry, S.; Fortenberry, R. C.; Hammer, N. I.; Delcamp, J. H. Counter Anion Effect on the Photophysical Properties of Emissive Indolizine-Cyanine Dyes in Solution and Solid State. *Molecules* **2018**, *23*, 3051.

3. Lewis, J. E.M.; Crowley, J. D. Exo- and Endo-Hedral Interactions of Counteranions with Tetracationic Pd_2L_4 Metallosupramolecular Architectures. *Supramolecular Chemistry*, **2014**, *26*, 173–181.

4. Zhang, D.; Ronson, T. K.; Mosquera, J.; Martinez, A.; Nitschke, J. R. Selective Anion Extraction and Recovery Using a $Fe^{II}_4L_4$ Cage. *Angew. Chem., Int. Ed.* **2018**, *57*, 3717−3721.

5. Allan, D.; Nowell, H.; Barnett, S.; Warren, M.; Wilcox, A.; Christensen, J.; Saunders, L.; Peach, A.; Hooper, M.; Zaja, L.; Patel, S.; Cahill, L.; Marshall, R.; Trimnell, S.; Foster, A.; Bates, T.; Lay, S.; Williams, M.; Hathaway, P.; Winter, G.; Gerstel, M.; Wooley, R., A Novel Dual Air-Bearing Fixed-χ Diffractometer for Small-Molecule Single-Crystal X-ray Diffraction on Beamline I19 at Diamond Light Source. *Crystals* **2017,** *7*, 336.

6. Rigaku *CrystalClear*, 2.0; Rigaku Americas and Rigaku Corporation.: 9009 TX, USA 1997-2009.

7. Farrugia, L., WinGX and ORTEP for Windows: an update. *J. Appl. Crystallogr.* **2012,** *45*, 849-854.

8. Palatinus, L.; Chapuis, G., SUPERFLIP - a computer program for the solution of crystal structures by charge flipping in arbitrary dimensions. *J. Appl. Crystallogr.* **2007,** *40*, 786-790.

9. Sheldrick, G. M., Crystal structure refinement with SHELXL. *Acta. Cryst.* **2015,** *C71*, 3-8.

10. van der Sluis, P.; Spek, A. L., BYPASS: an effective method for the refinement of crystal structures containing disordered solvent regions. *Acta Cryst.* **1990,** *A46*, 194-201.

11. Spek, A. L., *PLATON: A Multipurpose Crystallographic Tool*. Utrecht University: Utrecht, The Netherlands, 2008.

12. Maglic, J. B.; Lavendomme, R., An Easy-to-Use Program to Calculate Various Volumes and Surface Areas of Chemical Structures and Identify Cavities. *J. Appl. Cryst.* **2022**, *55*, 1033-1044.

13. SCIGRESS software version FJ 2.6 (EU 3.1.9) Build 5996.8255.20141202, Fujitsu Limited, Tokyo, Japan, 2013.
